# Correction: Evaluation of the impact of COVID-19 pandemic on hospital admission related to common infections: Risk prediction models to tackle antimicrobial resistance in primary care

**DOI:** 10.1371/journal.pone.0321408

**Published:** 2025-03-28

**Authors:** Ali Fahmi, Victoria Palin, Xiaomin Zhong, Ya-Ting Yang, Simon Watts, Darren M. Ashcroft, Ben Goldacre, Brian MacKenna, Louis Fisher, Jon Massey, Amir Mehrkar, Seb Bacon, the OpenS.A.F.E.L.Y. Collaborative, Kieran Hand, Tjeerd Pieter van Staa

In Fig 2, the legend that describes the meaning of each color used in the lines should have been included. Please see the correct Fig 2 here.

**Fig 2:**
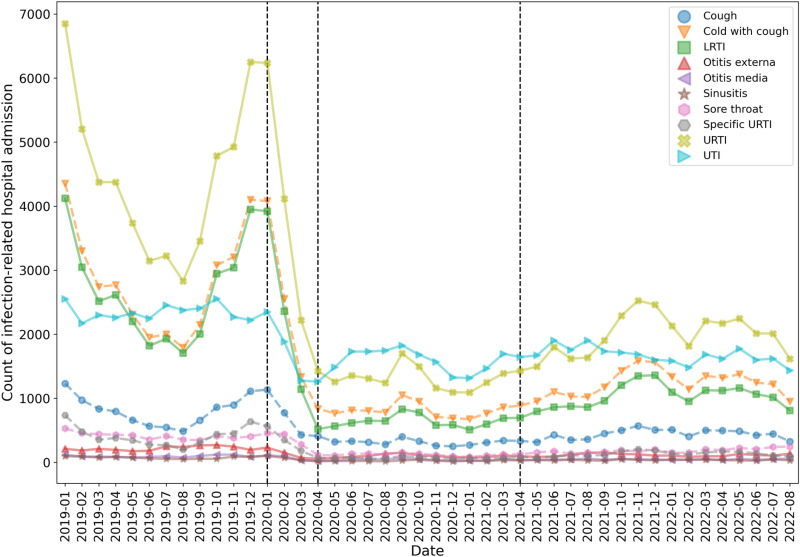
Count of hospital admissions related to upper respiratory tract infections (URTI; including specific URTI, cough, cold with cough, and sore throat), lower respiratory tract infection (LRTI), urinary tract infection (UTI), sinusitis, otitis media, and otitis externa.

## References

[1] Fahmi A, Palin V, Zhong X, Yang Y-T, Watts S, Ashcroft DM, et al. Evaluation of the impact of COVID-19 pandemic on hospital admission related to common infections: Risk prediction models to tackle antimicrobial resistance in primary care. PLoS One. 2024;19(12): e0311515. doi: 10.1371/journal.pone.0311515 39739781 PMC11687718

