# Supplementary material for: Evaluation of the impact of COVID-19 pandemic on hospital admission related to common infections: Risk prediction models to tackle antimicrobial resistance in primary care
Source: PLoS One. 2024 Dec 31;19(12):e0311515. doi: 10.1371/journal.pone.0311515 (PMC11687718; doi:10.1371/journal.pone.0311515)
Supplement: S2 Appendix — (DOCX) [file pone.0311515.s002.docx]

# Counts and rates of hospital admission cases

**S5 Table. Further counts and rates of hospital admission of patients with incident common infections, including lower respiratory tract infection (LRTI), upper respiratory tract infection (URTI), and urinary tract infection (UTI), with no prescribed antibiotics.**

|  | **Incident LRTI**  **with no ABs^1^** | **Incident URTI**  **with no ABs** | **Incident UTI**  **with no ABs** |
| --- | --- | --- | --- |
| **BMI^2^, N (rate)** |  |  |  |
| Underweight | 625 (85.7) | 1,335 (23.5) | 425 (64.4) |
| Healthy weight | 4,605 (74.1) | 9,930 (15.9) | 4,475 (57.1) |
| Overweight | 4,655 (70.6) | 10,360 (14.6) | 4,850 (63.9) |
| Obese | 5,010 (71.4) | 11,730 (16.1) | 5,170 (70.1) |
| Unknown | 3,020 (63.8) | 6,680 (14.9) | 3,215 (53.8) |
| **Smoking status, N (rate)** |  |  |  |
| Smoker | 2,515 (59.1) | 6,665 (12.0) | 1,825 (51.4) |
| Ex-smoker | 10,285 (80.9) | 22,145 (18.2) | 9,960 (74.1) |
| Never smoked | 5,075 (61.7) | 11,075 (14.1) | 6,305 (51.3) |
| Unknown | 40 (39.0) | 150 (11.4) | 45 (30.1) |
| **IMD^3^, N (rate)** |  |  |  |
| 1 (most deprived) | 4,210 (70.1) | 9,935 (15.7) | 3,830 (63.7) |
| 2 | 3,580 (71.6) | 8,205 (15.8) | 3,715 (65.0) |
| 3 | 3,620 (70.3) | 8,130 (15.6) | 3,825 (62.2) |
| 4 | 3,400 (73.6) | 7,275 (15.9) | 3,545 (61.9) |
| 5 (most affluent) | 2,780 (67.3) | 5,860 (14.8) | 2,890 (54.1) |
| Unknown | 325 (80.5) | 625 (15.0) | 330 (69.0) |
| **Season, N (rate)** |  |  |  |
| Spring | 4,335 (66.7) | 10,065 (14.8) | 4,600 (60.2) |
| Summer | 4,125 (78.2) | 9,180 (16.0) | 5,110 (62.6) |
| Autumn | 3,710 (69.8) | 8,335 (15.9) | 3,920 (61.9) |
| Winter | 5,745 (70.0) | 12,460 (15.8) | 4,510 (61.8) |
| **Region, N (rate)** |  |  |  |
| London | 470 (48.2) | 1,200 (11.6) | 585 (46.2) |
| North East | 1,190 (75.7) | 2,780 (16.5) | 1,060 (64.9) |
| North West | 1,670 (58.0) | 4,035 (13.0) | 1,565 (49.2) |
| East | 4,075 (70.2) | 9,050 (16.2) | 4,395 (63.8) |
| West Midlands | 830 (73.6) | 1,840 (18.5) | 760 (65.0) |
| Yorkshire and The Humber | 2,960 (72.3) | 6,440 (15.5) | 3,005 (69.5) |
| South East | 1,165 (73.1) | 2,470 (15.6) | 1,245 (60.5) |
| East Midlands | 3,475 (80.5) | 7,915 (17.6) | 3,330 (66.7) |
| South West | 2,085 (71.1) | 4,310 (14.3) | 2,190 (55.9) |
| ^1^ ABs, antibiotics prescribed or not.  ^2^ BMI, Body Mass Index recorded in the last 5 years.  ^3^ IMD, Multiple Deprivation Index, quintile measured from patient-level address. | | | |

**S6 Table. Counts and rates of hospital admission of patients with incident common infections with prescribed antibiotics, prevalent common infections with no prescribed antibiotics, and prevalent common infections with prescribed antibiotics. Common infections include lower respiratory tract infection (LRTI), upper respiratory tract infection (URTI), and urinary tract infection (UTI).**

|  | **LRTI** | | | **URTI** | | | **UTI** | | |
| --- | --- | --- | --- | --- | --- | --- | --- | --- | --- |
|  | **Incident** | **Prevalent** | | **Incident** | **Prevalent** | | **Incident** | **Prevalent** | |
|  | **With ABs^1^** | **No ABs** | **With ABs** | **With ABs** | **No ABs** | **With ABs** | **With ABs** | **No ABs** | **With ABs** |
| **Total, N cases** | 35,890 | 4,270 | 4,270 | 58,320 | 7,620 | 8,885 | 47,570 | 5,380 | 9,790 |
| **Age, N (rate^2^)** |  |  |  |  |  |  |  |  |  |
| 18-24 | 530 (8.5) | 60 (43.8) | 60 (20.6) | 2,640 (10.6) | 435 (28.2) | 360 (23.4) | 1,010 (7.8) | 100 (22.4) | 185 (15.6) |
| 25-34 | 1,320 (9.0) | 115 (32.7) | 115 (12.8) | 3,800 (8.9) | 500 (18.8) | 445 (16.6) | 1,790 (8.2) | 180 (28.1) | 290 (14.9) |
| 35-44 | 1,555 (8.7) | 155 (31.5) | 155 (11.2) | 3,385 (8.1) | 360 (12.2) | 390 (13.0) | 1,755 (8.4) | 160 (27.9) | 350 (17.4) |
| 45-54 | 2,735 (10.8) | 280 (36.7) | 280 (12.4) | 4,930 (9.8) | 650 (15.0) | 715 (16.9) | 2,850 (10.9) | 280 (36.4) | 555 (18.7) |
| 55-64 | 4,320 (14.4) | 450 (46.6) | 450 (14.8) | 7,125 (12.7) | 890 (16.5) | 1,100 (20.5) | 4,560 (16.0) | 475 (46.7) | 940 (24.2) |
| 65-74 | 7,245 (23.4) | 940 (78.8) | 940 (26.1) | 11,145 (20.1) | 1,540 (27.0) | 1,915 (31.9) | 9,340 (25.8) | 1,065 (68.7) | 2,025 (33.8) |
| 75+ | 18,185 (52.3) | 2,270 (110.9) | 2,270 (48.6) | 25,300 (46.0) | 3,245 (57.1) | 3,955 (58.5) | 26,265 (56.0) | 3,120 (114.3) | 5,455 (63.2) |
| **Sex, N (rate)** |  |  |  |  |  |  |  |  |  |
| Male | 15,765 (24.9) | 1,955 (82.5) | 1,955 (31.5) | 25,320 (20.5) | 3,435 (29.9) | 3,900 (35.1) | 18,065 (54.6) | 2,320 (105.0) | 3,465 (65.5) |
| Female | 20,125 (20.9) | 2,315 (64.7) | 2,315 (23.3) | 33,000 (16.3) | 4,185 (25.0) | 4,985 (27.0) | 29,505 (18.4) | 3,060 (55.5) | 6,325 (29.6) |
| **BMI^3^, N (rate)** |  |  |  |  |  |  |  |  |  |
| Underweight | 1,195 (40.4) | 170 (103.7) | 170 (48.9) | 1,795 (30.4) | 240 (44.4) | 280 (46.9) | 1,160 (31.7) | 120 (74.3) | 200 (40.1) |
| Healthy weight | 8,750 (26.2) | 1,140 (77.7) | 1,140 (32.8) | 13,855 (20.0) | 1,830 (28.4) | 2,175 (34.1) | 11,690 (22.9) | 1,275 (61.5) | 2,380 (34.4) |
| Overweight | 9,545 (22.4) | 1,175 (74.1) | 1,175 (26.9) | 15,050 (17.9) | 1,975 (25.3) | 2,305 (29.0) | 12,860 (25.3) | 1,470 (69.8) | 2,795 (38.0) |
| Obese | 10,570 (21.0) | 1,125 (64.8) | 1,125 (20.7) | 17,280 (17.5) | 2,225 (26.3) | 2,740 (28.3) | 13,560 (27.2) | 1,635 (82.1) | 2,810 (39.1) |
| Unknown | 5,830 (19.2) | 660 (66.2) | 660 (26.2) | 10,340 (15.3) | 1,350 (27.1) | 1,385 (27.8) | 8,295 (21.9) | 880 (63.2) | 1,610 (34.4) |
| **Ethnicity, N (rate)** |  |  |  |  |  |  |  |  |  |
| White | 20,175 (21.9) | 2,505 (68.2) | 2,505 (25.6) | 32,445 (17.8) | 4,365 (25.2) | 5,140 (29.1) | 27,055 (24.0) | 3,100 (65.7) | 5,785 (35.6) |
| Non-White | 1,770 (15.6) | 175 (59.1) | 175 (21.5) | 3,330 (12.0) | 400 (18.8) | 365 (18.8) | 1,910 (16.1) | 210 (57.6) | 355 (30.5) |
| Unknown | 13,945 (24.7) | 1,590 (80.3) | 1,590 (28.6) | 22,550 (19.5) | 2,855 (32.6) | 3,375 (33.7) | 18,605 (27.1) | 2,070 (78.4) | 3,650 (39.6) |
| **CCI^4^, N (rate)** |  |  |  |  |  |  |  |  |  |
| Very low | 13,125 (15.3) | 1,335 (50.4) | 1,335 (18.5) | 23,960 (12.6) | 2,930 (18.5) | 3,145 (21.2) | 19,785 (16.2) | 1,990 (47.3) | 3,830 (25.5) |
| Low | 15,105 (25.9) | 1,875 (78.2) | 1,875 (27.8) | 23,070 (21.3) | 3,115 (32.8) | 3,700 (32.8) | 18,125 (33.4) | 2,075 (84.3) | 3,825 (45.3) |
| Medium | 5,670 (44.7) | 785 (112.7) | 785 (45.1) | 8,415 (38.3) | 1,185 (52.4) | 1,495 (54.1) | 7,185 (53.6) | 940 (117.9) | 1,540 (62.4) |
| High | 1,510 (63.8) | 195 (126.2) | 195 (56.5) | 2,135 (52.9) | 290 (63.7) | 410 (73.9) | 1,895 (72.1) | 285 (144.7) | 455 (85.0) |
| Very high | 480 (68.0) | 75 (138.9) | 75 (71.4) | 740 (62.1) | 100 (70.4) | 140 (87.2) | 580 (79.7) | 90 (145.2) | 145 (87.9) |
| **Smoking status, N (rate)** |  |  |  |  |  |  |  |  |  |
| Smoker | 4,640 (14.6) | 460 (54.0) | 460 (15.9) | 8,535 (13.1) | 1,030 (21.8) | 1,160 (21.0) | 4,760 (21.1) | 480 (63.7) | 905 (34.6) |
| Ex-smoker | 20,445 (27.3) | 2,605 (82.8) | 2,605 (31.2) | 31,725 (22.0) | 4,365 (32.3) | 5,180 (35.9) | 25,935 (29.8) | 3,040 (81.0) | 5,410 (41.7) |
| Never smoked | 10,730 (20.4) | 1,195 (61.6) | 1,195 (24.5) | 17,795 (15.5) | 2,190 (22.2) | 2,510 (26.3) | 16,765 (20.2) | 1,855 (58.1) | 3,475 (31.6) |
| Unknown | 75 (15.0) | 10 (76.9) | 10 (44.4) | 270 (12.8) | 40 (38.5) | 35 (34.3) | 110 (17.2) | SN^5^ | 10 (17.7) |
| **IMD^6^, N (rate)** |  |  |  |  |  |  |  |  |  |
| 1 (most deprived) | 8,305 (21.0) | 940 (73.2) | 940 (23.7) | 13,835 (17.2) | 1,815 (28.8) | 2,060 (28.7) | 9,485 (25.5) | 1,105 (81.1) | 1,865 (39.7) |
| 2 | 7,110 (21.9) | 820 (72.2) | 820 (25.3) | 11,875 (17.4) | 1,520 (27.7) | 1,785 (29.5) | 9,190 (25.3) | 1,055 (75.0) | 1,925 (40.1) |
| 3 | 7,375 (23.2) | 880 (71.5) | 880 (27.4) | 11,840 (18.2) | 1,530 (25.9) | 1,875 (31.0) | 10,150 (25.2) | 1,175 (71.8) | 2,095 (37.3) |
| 4 | 6,865 (23.8) | 825 (71.3) | 825 (27.8) | 10,990 (19.1) | 1,475 (28.0) | 1,650 (31.2) | 9,535 (24.3) | 1,030 (63.6) | 2,010 (35.9) |
| 5 (most affluent) | 5,640 (23.0) | 725 (69.2) | 725 (29.2) | 8,840 (18.0) | 1,140 (23.8) | 1,360 (30.1) | 8,450 (22.6) | 925 (58.6) | 1,745 (31.7) |
| Unknown | 595 (21.8) | 80 (86.0) | 80 (28.3) | 940 (17.8) | 140 (30.5) | 150 (29.6) | 760 (25.1) | 95 (77.2) | 150 (34.8) |
| **Season, N (rate)** |  |  |  |  |  |  |  |  |  |
| Spring | 8,385 (21.9) | 1,005 (66.8) | 1,005 (25.9) | 14,190 (17.2) | 1,940 (24.1) | 2,135 (27.1) | 12,470 (24.9) | 1,390 (69.8) | 2,530 (36.4) |
| Summer | 6,855 (24.5) | 865 (81.1) | 865 (32.6) | 11,395 (18.4) | 1,600 (27.3) | 1,630 (31.2) | 13,210 (24.8) | 1,505 (72.6) | 2,680 (37.6) |
| Autumn | 7,825 (22.4) | 825 (70.9) | 825 (25.5) | 12,600 (18.1) | 1,500 (27.7) | 1,810 (31.1) | 10,205 (24.3) | 1,150 (66.9) | 2,170 (37.5) |
| Winter | 12,825 (21.9) | 1,575 (71.1) | 1,575 (24.7) | 20,140 (18.0) | 2,580 (29.0) | 3,310 (31.0) | 11,685 (24.3) | 1,340 (69.0) | 2,420 (35.7) |
| **Region, N (rate)** |  |  |  |  |  |  |  |  |  |
| London | 845 (17.5) | 100 (63.7) | 100 (35.0) | 1,880 (12.9) | 255 (19.0) | 190 (18.4) | 1,320 (22.0) | 190 (70.2) | 185 (31.5) |
| North East | 1,925 (22.2) | 280 (76.7) | 280 (31.8) | 3,055 (17.9) | 500 (28.9) | 470 (28.8) | 2,200 (25.7) | 280 (74.2) | 470 (40.4) |
| North West | 3,990 (22.4) | 480 (63.2) | 480 (23.4) | 6,455 (18.4) | 810 (22.8) | 1,060 (28.7) | 5,100 (24.3) | 535 (60.1) | 1,155 (35.3) |
| East | 8,025 (21.8) | 925 (72.7) | 925 (26.0) | 13,335 (16.8) | 1,690 (28.4) | 1,950 (28.7) | 11,550 (25.0) | 1,260 (74.9) | 2,235 (36.4) |
| West Midlands | 1,700 (22.6) | 185 (74.6) | 185 (26.9) | 2,930 (17.9) | 355 (31.0) | 405 (31.2) | 2,030 (25.0) | 240 (85.4) | 415 (39.2) |
| Yorkshire and The Humber | 6,480 (21.0) | 805 (70.0) | 805 (24.4) | 9,960 (17.7) | 1,350 (27.4) | 1,555 (29.7) | 7,945 (25.4) | 1,000 (70.1) | 1,840 (40.4) |
| South East | 2,040 (23.9) | 245 (73.7) | 245 (30.4) | 3,460 (19.0) | 480 (27.8) | 560 (32.9) | 2,915 (24.5) | 290 (59.1) | 550 (35.0) |
| East Midlands | 7,170 (23.8) | 830 (76.9) | 830 (24.7) | 11,270 (19.1) | 1,485 (29.2) | 1,855 (32.6) | 9,255 (24.3) | 995 (71.4) | 2,040 (36.3) |
| South West | 3,720 (25.6) | 420 (71.6) | 420 (34.4) | 5,985 (20.1) | 695 (25.0) | 845 (33.5) | 5,250 (23.6) | 590 (64.8) | 900 (33.8) |
| **Flu vaccination, N (rate)** |  |  |  |  |  |  |  |  |  |
| Yes | 23,865 (30.7) | 2,925 (86.0) | 2,925 (31.3) | 35,715 (25.6) | 4,645 (34.3) | 5,815 (37.9) | 31,140 (35.6) | 3,600 (87.0) | 6,670 (45.6) |
| No | 12,025 (14.6) | 1,345 (52.8) | 1,345 (19.8) | 22,610 (12.1) | 2,975 (20.3) | 3,070 (21.6) | 16,425 (15.5) | 1,780 (49.6) | 3,125 (26.0) |
| **Period** |  |  |  |  |  |  |  |  |  |
| Pre-pandemic | 18,865 (24.6) | 2,140 (72.0) | 2,140 (24.9) | 28,740 (20.3) | 3,595 (31.2) | 4,660 (34.2) | 16,395 (27.4) | 1,995 (77.9) | 3,615 (41.9) |
| During pandemic | 7,905 (23.1) | 1,110 (72.9) | 1,110 (28.9) | 13,710 (18.0) | 1,970 (23.6) | 2,205 (28.3) | 14,615 (22.0) | 1,580 (61.9) | 2,970 (32.3) |
| After 2^nd^ lockdown | 9,125 (18.7) | 1,020 (70.1) | 1,020 (27.5) | 15,870 (14.7) | 2,055 (24.7) | 2,020 (24.8) | 16,555 (24.7) | 1,805 (69.2) | 3,210 (36.3) |
| ^1^ ABs, antibiotics prescribed or not.  ^2^ Rate is the number of cases per 1000 patients with common infection, calculated by dividing the count of infection-related hospital admission cases (numerator) by the count of infection diagnosis (denominator) and then multiplied by 1000.  ^3^ BMI, Body Mass Index recorded in the last 5 years.  ^4^ CCI, Charlson Comorbidities Index, measured from 17 weighted conditions, including myocardial infarction, congestive heart failure, peripheral vascular disease, cerebrovascular disease, dementia, chronic pulmonary disease, Connective tissue disease, ulcer disease, mild liver disease, diabetes, hemiplegia, moderate or severe renal disease, diabetes with complications, any malignancy (including leukaemia and lymphoma), moderate or severe liver disease, metastatic solid tumour, and AIDS.  ^5^ SN, small numbers, equal or less than 5.  ^6^ IMD, Multiple Deprivation Index, quintile measured from patient-level address. | | | | | | | | | |

**S7 Table. Counts and rate of hospital admission of patients with other common infections, including sinusitis, otitis media, and otitis externa. The other common infections include incident infections with no prescribed antibiotics, incident infections with prescribed antibiotics, prevalent infections with no prescribed antibiotics, and prevalent infections with prescribed antibiotics.**

|  | **Sinusitis** | | | | **Otitis media** | | | | **Otitis externa** | | | |
| --- | --- | --- | --- | --- | --- | --- | --- | --- | --- | --- | --- | --- |
|  | **Incident** | | **Prevalent** | | **Incident** | | **Prevalent** | | **Incident** | | **Prevalent** | |
|  | **No** **ABs^1^** | **With ABs** | **No ABs** | **With ABs** | **No ABs** | **With ABs** | **No ABs** | **With ABs** | **No ABs** | **With ABs** | **No ABs** | **With ABs** |
| **Total, N cases** | 535 | 1,205 | 75 | 130 | 755 | 1,545 | 155 | 170 | 3,015 | 1,660 | 1,010 | 465 |
| **Age, N (rate^2^)** |  |  |  |  |  |  |  |  |  |  |  |  |
| 18-24 | 40 (4.3) | 75 (3.9) | SN^3^ | SN | 50 (6.0) | 130 (5.4) | 10 (11.6) | 15 (11.5) | 205 (4.2) | 115 (8.7) | 45 (10.3) | 25 (14.8) |
| 25-34 | 75 (3.1) | 175 (2.9) | 15 (6.5) | 15 (3.9) | 115 (7.6) | 290 (6.1) | 20 (11.0) | 35 (10.9) | 375 (4.1) | 255 (9.3) | 75 (9.0) | 45 (11.8) |
| 35-44 | 80 (3.2) | 175 (2.4) | 10 (3.5) | 25 (4.6) | 95 (7.2) | 225 (5.2) | 20 (11.8) | 25  (8.3) | 315 (3.4) | 210 (7.7) | 105 (12.7) | 45 (11.7) |
| 45-54 | 80 (3.1) | 185 (2.4) | 15 (4.8) | 25 (4.2) | 95 (6.8) | 225 (5.4) | 25 (13.6) | 20  (6.7) | 395 (3.7) | 245 (8.0) | 105 (10.4) | 40 (9.3) |
| 55-64 | 85 (3.9) | 220 (3.3) | 15 (5.8) | 15 (3.0) | 110 (8.8) | 190 (5.6) | 10 (6.4) | 20  (8.4) | 365 (3.7) | 220 (8.4) | 105 (11.0) | 60 (15.1) |
| 65-74 | 70 (4.3) | 195 (4.1) | 10 (5.3) | 20 (5.5) | 120 (11.5) | 205 (8.3) | 20 (16.3) | 20 (11.3) | 515 (5.8) | 245 (12.2) | 155 (16.8) | 80 (23.8) |
| 75+ | 105 (14.5) | 185 (10.0) | 15 (21.0) | 20 (15.9) | 175 (23.2) | 285 (19.6) | 45 (50.0) | 35 (34.0) | 845 (13.6) | 370 (28.9) | 415 (53.5) | 170 (64.3) |
| **Sex, N (rate)** |  |  |  |  |  |  |  |  |  |  |  |  |
| Male | 205 (4.5) | 350 (3.6) | 25 (5.6) | 35 (5.2) | 350 (10.6) | 535 (6.7) | 60 (15.4) | 60 (10.5) | 1,305 (5.3) | 640 (10.9) | 540 (22.2) | 225 (24.1) |
| Female | 330 (3.9) | 855 (3.2) | 50 (5.1) | 95 (4.9) | 405 (8.4) | 1,010 (6.8) | 95 (15.8) | 110 (11.0) | 1,710 (4.9) | 1,020 (10.3) | 470 (14.1) | 240 (16.8) |
| **BMI^4^, N (rate)** |  |  |  |  |  |  |  |  |  |  |  |  |
| Underweight | 15 (8.6) | 15 (3.2) | - | SN | 15 (14.2) | 20  (7.0) | SN | SN | 45 (6.2) | 15 (8.3) | SN | SN |
| Healthy weight | 125 (3.9) | 285 (3.3) | 20 (5.7) | 30 (4.9) | 155 (9.0) | 300 (6.7) | 25 (12.4) | 30 (10.6) | 585 (4.8) | 320 (10.8) | 195 (16.6) | 80 (17.9) |
| Overweight | 130 (3.8) | 295 (3.2) | 20 (5.3) | 30 (4.4) | 210 (10.6) | 365 (6.6) | 35 (14.4) | 45 (11.7) | 785 (5.2) | 440 (11.5) | 325 (21.1) | 165 (27.3) |
| Obese | 150 (4.9) | 385 (4.0) | 25 (7.1) | 45 (6.2) | 215 (10.0) | 550 (8.3) | 50 (17.7) | 65 (13.5) | 1,035 (6.4) | 595 (12.2) | 345 (20.6) | 150 (20.1) |
| Unknown | 115 (3.7) | 225 (2.7) | 15 (78.9) | 25 (4.5) | 165 (7.8) | 310 (5.2) | 35 (13.8) | 30  (7.5) | 565 (3.8) | 290 (7.4) | 135 (10.3) | 60 (11.1) |
| **Ethnicity, N (rate)** |  |  |  |  |  |  |  |  |  |  |  |  |
| White | 290 (3.8) | 705 (3.3) | 55 (12.9) | 90 (5.5) | 455 (10.0) | 865 (6.8) | 80 (14.0) | 105 (11.8) | 1,750 (5.2) | 920 (10.1) | 585 (16.9) | 270 (19.3) |
| Non-White | 50 (4.9) | 85 (3.6) | SN | SN | 55 (7.8) | 135 (6.8) | 20 (23.8) | 10  (8.1) | 225 (4.9) | 105 (9.5) | 60 (15.6) | 25 (18.1) |
| Unknown | 200 (4.7) | 420 (3.4) | 20 (22.3) | 35 (4.1) | 250 (8.9) | 550 (6.7) | 50 (14.9) | 55 (9.9) | 1,040 (5.1) | 630 (11.3) | 370 (19.2) | 165 (20.0) |
| **CCI^5^, N (rate)** |  |  |  |  |  |  |  |  |  |  |  |  |
| Very low | 305 (3.2) | 655 (2.5) | 45 (4.5) | 65 (3.6) | 415 (7.1) | 920 (5.5) | 85 (12.0) | 90  (8.0) | 1,540 (3.6) | 925 (8.3) | 440 (11.0) | 195 (12.1) |
| Low | 175 (5.6) | 415 (4.6) | 25 (7.0) | 55 (7.6) | 245 (13.3) | 445 (8.5) | 40 (17.4) | 50 (13.1) | 985 (7.3) | 490 (13.0) | 335 (23.9) | 165 (28.0) |
| Medium | 40 (10.7) | 105 (9.0) | SN | SN | 75 (24.4) | 140 (18.0) | 20 (50.0) | 20 (36.0) | 350 (14.5) | 175 (26.5) | 170 (57.0) | 75 (58.8) |
| High | 10 (16.3) | 20 (11.9) | SN | SN | 20 (37.0) | 30 (23.1) | SN | SN | 95 (23.1) | 55 (48.0) | 60 (95.2) | 15 (60.0) |
| Very high | SN | 10 (19.8) | SN | SN | SN | 10 (29.0) | SN | SN | 40 (36.9) | 15 (50.8) | 15 (103.4) | 10 (222.2) |
| **Smoking status, N (rate)** |  |  |  |  |  |  |  |  |  |  |  |  |
| Smoker | 90 (4.7) | 195 (3.4) | 10 (1.7) | 20 (4.8) | 155 (10.6) | 295 (6.5) | 25 (14.3) | 30 (10.2) | 495 (5.1) | 295 (9.2) | 120 (13.1) | 70 (16.6) |
| Ex-smoker | 245 (4.5) | 550 (3.6) | 40 (4.4) | 55 (4.8) | 325 (10.1) | 660 (7.5) | 65 (16.2) | 80 (12.5) | 1,460 (5.9) | 765 (11.8) | 540 (21.6) | 245 (23.8) |
| Never smoked | 195 (3.5) | 460 (3.1) | 30 (4.9) | 55 (5.2) | 275 (8.2) | 585 (6.3) | 60 (14.7) | 60  (9.6) | 1,045 (4.3) | 600 (10.1) | 345 (14.9) | 150 (16.6) |
| Unknown | SN | SN | - | - | SN | SN | SN | SN | 10 (1.9) | SN | SN | - |
| **IMD^6^, N (rate)** |  |  |  |  |  |  |  |  |  |  |  |  |
| 1 (most deprived) | 120 (4.8) | 235 (3.6) | 10 (43.5) | 20 (4.3) | 180 (9.5) | 415 (7.2) | 30 (13.3) | 55 (13.8) | 690 (5.6) | 400 (11.0) | 225 (19.8) | 100 (20.4) |
| 2 | 100 (4.0) | 230 (3.3) | 15 (5.9) | 25 (5.0) | 150 (9.1) | 330 (6.9) | 35 (18.6) | 40 (12.2) | 580 (5.1) | 350 (10.9) | 175 (16.7) | 95 (21.2) |
| 3 | 105 (3.9) | 280 (3.6) | 20 (7.5) | 35 (6.4) | 135 (8.4) | 275 (6.2) | 40 (20.4) | 35 (12.0) | 635 (5.2) | 320 (10.0) | 190 (16.0) | 85 (17.3) |
| 4 | 100 (3.8) | 225 (3.0) | 15 (4.9) | 25 (4.5) | 150 (10.2) | 250 (6.2) | 20 (10.7) | 20  (7.0) | 550 (4.8) | 300 (10.3) | 230 (19.7) | 85 (17.9) |
| 5 (most affluent) | 100 (4.1) | 215 (3.1) | 20 (7.7) | 30 (5.8) | 130 (9.8) | 240 (7.0) | 25 (14.1) | 20  (8.3) | 505 (4.6) | 270 (10.6) | 175 (15.4) | 85 (20.1) |
| Unknown | 10 (4.6) | 20 (3.4) | SN | SN | 15 (11.1) | 35 (9.2) | SN | SN | 50 (5.4) | 20 (7.9) | 20 (21.9) | 10 (26.0) |
| **Season, N (rate)** |  |  |  |  |  |  |  |  |  |  |  |  |
| Spring | 150 (4.2) | 305 (3.1) | 20 (363.6) | 35 (6.7) | 180 (9.0) | 350 (6.2) | 30 (12.4) | 35  (9.1) | 720 (5.1) | 355 (9.5) | 235 (17.9) | 85 (14.7) |
| Summer | 130 (5.0) | 240 (3.6) | 15 (5.3) | 30 (4.1) | 195 (9.4) | 410 (7.3) | 40 (17.1) | 55 (14.3) | 850 (5.2) | 470 (11.0) | 265 (16.9) | 115 (21.9) |
| Autumn | 105 (3.8) | 255 (3.4) | 20 (9.5) | 30 (461.5) | 175 (10.1) | 375 (7.7) | 35 (16.3) | 40 (12.1) | 725 (5.5) | 415 (11.6) | 290 (20.8) | 120 (923.1) |
| Winter | 150 (3.7) | 410 (3.3) | 25 (6.0) | 35 (7.4) | 210 (9.3) | 410 (6.1) | 45 (15.0) | 35  (7.5) | 720 (4.7) | 420 (10.1) | 225 (15.1) | 140 (21.7) |
| **Region, N (rate)** |  |  |  |  |  |  |  |  |  |  |  |  |
| London | 20 (3.3) | 40 (3.1) | SN | SN | 30 (8.1) | 40  (4.6) | SN | SN | 125 (5.2) | 45 (7.4) | 35 (16.5) | 10 (14.5) |
| North East | 35 (5.0) | 45 (2.8) | 10 (18.3) | SN | 50 (10.5) | 80 (7.0) | 15 (28.6) | 10 (12.3) | 160 (5.1) | 100 (12.3) | 60 (19.1) | 25 (19.2) |
| North West | 60 (3.7) | 135 (3.5) | 10 (13.4) | 15 (4.6) | 80 (8.7) | 170 (6.8) | 10 (8.1) | 15  (8.1) | 335 (5.1) | 155 (9.6) | 120 (17.3) | 50 (19.2) |
| East | 115 (3.9) | 285 (2.9) | 20 (6.2) | 40 (5.7) | 160 (7.5) | 320 (5.3) | 30 (11.8) | 25  (6.0) | 520 (3.9) | 320 (8.0) | 145 (11.6) | 65 (11.3) |
| West Midlands | 25 (5.4) | 55 (4.3) | SN | SN | 35 (9.9) | 70 (6.8) | SN | SN | 110 (5.1) | 65 (11.1) | 25 (14.8) | 10 (13.9) |
| Yorkshire and The Humber | 90 (4.1) | 215 (3.7) | 15 (31.6) | 15 (3.4) | 130 (9.5) | 280 (6.9) | 30 (16.5) | 35 (12.8) | 525 (5.3) | 300 (11.8) | 180 (18.0) | 85 (22.1) |
| South East | 30 (3.5) | 75 (3.3) | SN | SN | 45 (10.3) | 80 (7.0) | 10 (19.0) | 10 (13.6) | 190 (4.9) | 95 (9.6) | 60 (14.7) | 30 (18.9) |
| East Midlands | 95 (4.5) | 245 (3.6) | 10 (3.0) | 25 (4.9) | 165 (12.3) | 355 (8.2) | 30 (17.0) | 45 (14.0) | 655 (6.0) | 385 (12.6) | 250 (23.1) | 125 (25.7) |
| South West | 65 (4.5) | 120 (3.3) | SN | 10 (4.5) | 70 (10.0) | 150 (8.1) | 15 (22.2) | 15 (15.3) | 390 (5.7) | 195 (12.5) | 135 (21.2) | 65 (28.2) |
| **Flu vaccination, N (rate)** |  |  |  |  |  |  |  |  |  |  |  |  |
| Yes | 235 (6.3) | 555 (5.1) | 40 (9.2) | 65 (7.5) | 360 (14.8) | 630 (10.0) | 80 (25.9) | 85 (18.2) | 1,530 (7.8) | 765 (15.6) | 590 (27.5) | 275 (32.7) |
| No | 300 (3.2) | 655 (2.6) | 40 (4.0) | 60 (3.4) | 400 (7.1) | 920 (5.5) | 75 (11.0) | 85  (7.7) | 1,485 (3.8) | 895 (8.2) | 425 (11.7) | 185 (12.1) |
| **Period** |  |  |  |  |  |  |  |  |  |  |  |  |
| Pre-pandemic | 230 (4.5) | 545 (3.8) | 25 (5.5) | 60 (7.5) | 320 (10.8) | 665 (8.0) | 70 (18.2) | 80 (13.1) | 1,270 (5.7) | 690 (12.6) | 465 (20.8) | 200 (29.5) |
| During pandemic | 155 (4.2) | 285 (2.8) | 25 (9.1) | 25 (2.8) | 205 (8.5) | 415 (5.7) | 35 (11.0) | 50  (9.7) | 785 (4.4) | 435 (8.5) | 235 (13.3) | 140 (22.7) |
| After 2^nd^ lockdown | 150 (3.6) | 375 (3.1) | 30 (6.7) | 40 (4.8) | 230 (8.4) | 465 (6.3) | 45 (15.6) | 40  (9.0) | 955 (5.0) | 535 (10.3) | 310 (17.5) | 125 (16.3) |
| ^1^ ABs, antibiotics prescribed or not.  ^2^ Rate is the number of cases per 1000 patients with common infection, calculated by dividing the count of infection-related hospital admission cases (numerator) by the count of infection diagnosis (denominator) and then multiplied by 1000.  ^3^ SN, small numbers, equal or less than 5.  ^4^ BMI, Body Mass Index recorded in the last 5 years.  ^5^ CCI, Charlson Comorbidities Index, measured from 17 weighted conditions, including myocardial infarction, congestive heart failure, peripheral vascular disease, cerebrovascular disease, dementia, chronic pulmonary disease, Connective tissue disease, ulcer disease, mild liver disease, diabetes, hemiplegia, moderate or severe renal disease, diabetes with complications, any malignancy (including leukaemia and lymphoma), moderate or severe liver disease, metastatic solid tumour, and AIDS.  ^6^ IMD, Multiple Deprivation Index, quintile measured from patient-level address. | | | | | | | | | | | | |

**S8 Table. Counts and rates of hospital admission of patients with URTI infections, including upper respiratory tract infection (URTI), cough, cold with cough, and sore throat. These infections include incident infections with no prescribed antibiotics, incident infections with prescribed antibiotics, prevalent infections with no prescribed antibiotics, and prevalent infections with prescribed antibiotics.**

|  | **URTI** | | | | **Cough** | | | | **Cold with cough** | | | | **Sore throat** | | | |
| --- | --- | --- | --- | --- | --- | --- | --- | --- | --- | --- | --- | --- | --- | --- | --- | --- |
|  | **Incident** | | **Prevalent** | | **Incident** | | **Prevalent** | | **Incident** | | **Prevalent** | | **Incident** | | **Prevalent** | |
|  | **No** **ABs^1^** | **With ABs** | **No ABs** | **With ABs** | **No** **ABs** | **With ABs** | **No ABs** | **With ABs** | **No** **ABs** | **With ABs** | **No ABs** | **With ABs** | **No** **ABs** | **With ABs** | **No ABs** | **With ABs** |
| **Total, N cases** | 2,600 | 5,880 | 205 | 345 | 10,145 | 10,605 | 1,230 | 1,165 | 24,055 | 36,010 | 5,305 | 6,725 | 3,240 | 5,840 | 885 | 650 |
| **Age, N (rate^2^)** |  |  |  |  |  |  |  |  |  |  |  |  |  |  |  |  |
| 18-24 | 140 (6.1) | 180 (7.4) | 10 (15.6) | SN^3^ | 195 (5.5) | 175 (6.6) | 15  (5.9) | 10  (7.8) | 385 (8.0) | 525 (7.9) | 65 (14.0) | 55 (12.8) | 845 (16.4) | 1,760 (13.4) | 345 (45.6) | 290 (31.7) |
| 25-34 | 230 (5.9) | 325 (6.3) | 10  (7.6) | 15 (9.1) | 380 (5.6) | 480 (8.0) | 50  (8.5) | 50 (14.5) | 795 (8.7) | 1,380 (9.0) | 175 (15.5) | 210 (17.0) | 750 (11.5) | 1,615 (10.1) | 265 (32.8) | 175 (18.6) |
| 35-44 | 245 (6.9) | 370 (6.4) | SN | 25 (11.8) | 470 (5.8) | 555 (7.7) | 55  (6.9) | 50 (10.5) | 985 (9.5) | 1,640 (8.8) | 190 (12.6) | 250 (14.0) | 455 (9.4) | 820 (8.2) | 110 (22.6) | 65 (12.3) |
| 45-54 | 255 (7.1) | 560 (8.0) | 20 (11.4) | 30 (10.9) | 850 (6.3) | 995 (9.7) | 140 (10.4) | 125 (16.3) | 1,740 (10.8) | 2,815 (10.6) | 420 (17.3) | 520 (18.0) | 335 (8.5) | 555 (8.7) | 70 (18.7) | 40 (13.3) |
| 55-64 | 305 (9.5) | 795 (10.7) | 20 (11.7) | 40 (12.4) | 1,440 (7.4) | 1,470 (11.6) | 185 (10.2) | 160 (15.7) | 2,930 (13.2) | 4,480 (14.0) | 640 (20.4) | 875 (22.8) | 290 (9.5) | 385 (9.5) | 45 (17.2) | 25 (13.3) |
| 65-74 | 470 (18.9) | 1,095 (16.5) | 45 (33.7) | 75 (23.7) | 2,455 (10.4) | 2,355 (18.0) | 295 (15.3) | 295 (26.0) | 5,320 (20.1) | 7,385 (22.2) | 1,180 (34.4) | 1,510 (33.9) | 245 (11.4) | 310 (13.5) | 25 (12.9) | 30 (29.1) |
| 75+ | 955 (42.2) | 2,555 (41.3) | 95 (64.0) | 160 (49.0) | 4,360 (22.6) | 4,575 (38.7) | 495 (31.0) | 475 (44.5) | 11,905 (48.1) | 17,780 (49.7) | 2,630 (68.8) | 3,305 (62.2) | 325 (24.4) | 390 (30.9) | 30 (26.7) | 20 (37.4) |
| **Sex, N (rate)** |  |  |  |  |  |  |  |  |  |  |  |  |  |  |  |  |
| Male | 1,070 (14.4) | 2,485 (16.6) | 70 (22.4) | 150 (25.6) | 5,005 (11.6) | 4,720 (18.6) | 560 (15.9) | 525 (27.4) | 11,740 (22.7) | 15,935 (23.8) | 2,460 (37.1) | 3,010 (38.8) | 1,425 (15.4) | 2,185 (13.6) | 350 (34.0) | 215 (25.3) |
| Female | 1,530 (11.0) | 3,395 (13.2) | 135 (20.4) | 195 (17.8) | 5,140 (10.0) | 5,885 (15.4) | 670 (13.9) | 640 (21.2) | 12,315 (19.8) | 20,075 (19.8) | 2,845 (30.6) | 3,715 (30.5) | 1,815 (10.2) | 3,655 (9.9) | 535 (27.2) | 435 (20.0) |
| **BMI^4^, N (rate)** |  |  |  |  |  |  |  |  |  |  |  |  |  |  |  |  |
| Underweight | 75 (21.1) | 195 (28.8) | 10 (3.9) | 10 (37.0) | 375 (17.1) | 325 (26.9) | 30 (20.8) | 35 (36.1) | 825 (30.8) | 1,165 (36.6) | 175 (54.0) | 225 (53.9) | 65 (13.9) | 110 (13.2) | 25 (47.2) | 10 (18.3) |
| Healthy weight | 625 (12.8) | 1,355 (16.0) | 45 (11.6) | 90 (25.3) | 2,570 (11.1) | 2,515 (18.6) | 275 (14.7) | 270 (25.7) | 6,060 (21.8) | 8,715 (24.5) | 1,320 (36.2) | 1,690 (39.3) | 675 (10.4) | 1,270 (10.8) | 190 (26.4) | 125 (18.4) |
| Overweight | 675 (12.9) | 1,575 (14.8) | 55 (18.7) | 90 (20.1) | 2,700 (9.8) | 2,760 (16.1) | 320 (13.3) | 300 (21.8) | 6,345 (19.6) | 9,570 (21.2) | 1,445 (32.2) | 1,800 (32.7) | 640 (10.4) | 1,145 (10.3) | 155 (23.6) | 115 (18.6) |
| Obese | 720 (12.9) | 1,790 (14.4) | 45 (21.8) | 100 (17.5) | 3,155 (11.3) | 3,370 (16.6) | 420 (16.2) | 410 (24.4) | 7,070 (21.4) | 10,710 (20.1) | 1,570 (32.1) | 2,075 (31.1) | 780 (12.5) | 1,415 (10.8) | 185 (27.4) | 160 (21.0) |
| Unknown | 505 (9.6) | 965 (11.6) | 45 (236.8) | 55 (19.9) | 1,345 (9.8) | 1,630 (14.2) | 180 (13.7) | 150 (20.5) | 3,750 (20.8) | 5,845 (18.6) | 795 (30.8) | 940 (30.7) | 1,080 (14.1) | 1,900 (11.6) | 330 (37.0) | 235 (25.8) |
| **Ethnicity, N (rate)** |  |  |  |  |  |  |  |  |  |  |  |  |  |  |  |  |
| White | 1,380 (11.7) | 3,085 (14.5) | 105 (36.3) | 175 (19.1) | 5,890 (10.2) | 5,990 (16.4) | 705 (13.4) | 690 (22.9) | 13,805 (20.2) | 20,390 (21.0) | 3,090 (31.4) | 3,950 (32.7) | 1,730 (12.1) | 2,980 (10.9) | 465 (28.1) | 320 (19.8) |
| Non-White | 185 (8.1) | 495 (9.6) | 15 (8.5) | 20 (10.4) | 505 (8.8) | 585 (11.3) | 65 (11.0) | 50 (15.0) | 1,110 (14.6) | 1,760 (14.7) | 245 (21.4) | 245 (21.3) | 325 (9.7) | 490 (9.1) | 80 (27.4) | 50 (18.5) |
| Unknown | 1,030 (14.1) | 2,295 (16.1) | 85 (84.2) | 150 (26.3) | 3,750 (12.0) | 4,030 (18.4) | 460 (18.7) | 425 (26.7) | 9,140 (24.1) | 13,855 (23.5) | 1,965 (39.6) | 2,525 (37.6) | 1,180 (12.5) | 2,370 (11.6) | 345 (32.9) | 275 (24.1) |
| **CCI^5^, N (rate)** |  |  |  |  |  |  |  |  |  |  |  |  |  |  |  |  |
| Very low | 1,160 (7.8) | 2,385 (9.8) | 85 (13.98) | 135 (15.0) | 3,915 (7.6) | 4,050 (11.8) | 435 (9.4) | 365 (15.4) | 9,140 (14.6) | 13,290 (14.8) | 1,740 (20.9) | 2,170 (23.5) | 2,280 (11.0) | 4,240 (10.1) | 665 (28.8) | 470 (20.0) |
| Low | 970 (18.3) | 2,395 (18.3) | 90 (31.1) | 140 (23.2) | 4,140 (12.2) | 4,375 (18.8) | 535 (18.6) | 515 (26.3) | 9,730 (24.3) | 15,005 (24.3) | 2,290 (39.9) | 2,895 (35.7) | 740 (13.6) | 1,295 (12.9) | 200 (33.5) | 145 (24.0) |
| Medium | 320 (35.5) | 800 (31.6) | 20 (33.9) | 55 (39.1) | 1,550 (21.3) | 1,635 (33.1) | 200 (30.1) | 205 (41.8) | 3,810 (42.2) | 5,750 (42.8) | 955 (65.1) | 1,210 (58.3) | 160 (21.7) | 230 (21.8) | 10 (14.4) | 20 (35.4) |
| High | 115 (67.8) | 220 (48.0) | 10 (87.0) | 10 (40.8) | 415 (32.1) | 395 (44.1) | 45 (36.0) | 60 (62.2) | 1,010 (60.1) | 1,470 (59.0) | 230 (76.0) | 330 (78.1) | 40 (29.3) | 50 (26.0) | 10 (62.5) | SN |
| Very high | 30 (60.6) | 75 (55.1) | - | SN | 125 (37.2) | 145 (56.8) | 15 (40.5) | 20 (76.9) | 360 (74.3) | 495 (66.8) | 85 (87.6) | 115 (93.9) | 20 (44.0) | 25 (42.7) | SN | SN |
| **Smoking status, N (rate)** |  |  |  |  |  |  |  |  |  |  |  |  |  |  |  |  |
| Smoker | 390 (11.6) | 750 (10.2) | 20  (4.9) | 45 (16.2) | 1,820 (8.2) | 1,650 (12.2) | 150 (10.7) | 145 (15.0) | 3,725 (14.6) | 4,840 (14.2) | 660 (24.8) | 845 (22.7) | 735 (16.5) | 1,295 (12.8) | 200 (37.5) | 130 (23.4) |
| Ex-smoker | 1,290 (15.4) | 3,180 (18.5) | 120 (20.6) | 200 (26.0) | 5,875 (12.4) | 5,975 (20.1) | 740 (18.1) | 720 (29.4) | 13,840 (24.6) | 20,505 (25.9) | 3,195 (40.1) | 4,050 (39.8) | 1,135 (11.9) | 2,060 (11.5) | 305 (29.3) | 210 (20.8) |
| Never smoked | 900 (9.6) | 1,925 (12.1) | 65 (15.2) | 100 (15.9) | 2,440 (9.9) | 2,955 (14.6) | 335 (11.9) | 300 (19.8) | 6,450 (20.3) | 10,585 (19.5) | 1,435 (27.3) | 1,820 (30.3) | 1,280 (10.2) | 2,325 (9.7) | 355 (26.0) | 290 (20.7) |
| Unknown | 10 (5.2) | 20 (10.2) | - | - | 10  (3.9) | 20 (10.9) | SN | SN | 40 (10.2) | 75 (14.5) | 10 (31.7) | 10 (35.1) | 90 (19.1) | 155 (12.8) | 25 (45.5) | 20 (31.2) |
| **IMD^6^, N (rate)** |  |  |  |  |  |  |  |  |  |  |  |  |  |  |  |  |
| 1 (most deprived) | 595 (12.9) | 1,510 (14.7) | 40 (19.4) | 80 (18.0) | 2,655 (11.2) | 2,660 (16.5) | 290 (15.7) | 275 (22.7) | 5,915 (20.8) | 8,320 (20.1) | 1,265 (35.0) | 1,570 (32.4) | 770 (11.9) | 1,345 (10.7) | 215 (33.3) | 135 (20.1) |
| 2 | 515 (12.1) | 1,220 (13.8) | 45 (23.0) | 85 (23.1) | 2,115 (11.1) | 2,230 (16.3) | 255 (16.1) | 245 (24.0) | 4,865 (21.1) | 7,255 (20.9) | 1,050 (33.8) | 1,330 (32.9) | 710 (12.9) | 1,165 (10.5) | 165 (27.5) | 130 (20.8) |
| 3 | 500 (11.3) | 1,155 (14.4) | 40 (20.4) | 65 (19.4) | 2,090 (10.9) | 2,105 (16.5) | 245 (13.9) | 255 (24.4) | 4,890 (21.1) | 7,370 (21.9) | 1,070 (32.1) | 1,420 (35.1) | 655 (12.2) | 1,210 (11.4) | 180 (29.6) | 135 (21.7) |
| 4 | 525 (12.9) | 1,035 (15.0) | 40 (21.9) | 70 (25.8) | 1,780 (10.7) | 1,940 (17.8) | 255 (16.4) | 215 (25.6) | 4,415 (21.9) | 6,930 (23.0) | 1,035 (34.9) | 1,255 (34.7) | 555 (11.5) | 1,090 (11.4) | 145 (25.9) | 110 (19.6) |
| 5 (most affluent) | 425 (11.6) | 860 (14.5) | 40 (22.8) | 40 (17.4) | 1,370 (9.6) | 1,505 (16.3) | 165 (11.5) | 160 (21.7) | 3,575 (20.7) | 5,545 (21.6) | 780 (29.5) | 1,035 (34.0) | 490 (11.1) | 930 (11.1) | 155 (29.2) | 125 (25.1) |
| Unknown | 35 (11.2) | 95 (14.0) | SN | - | 135 (8.8) | 165 (16.4) | 15 (11.3) | 20 (24.1) | 395 (21.3) | 585 (21.1) | 100 (38.9) | 120 (34.7) | 60 (13.3) | 100 (11.9) | 20 (36.4) | 15 (30.6) |
| **Season, N (rate)** |  |  |  |  |  |  |  |  |  |  |  |  |  |  |  |  |
| Spring | 615 (12.2) | 1,375 (13.9) | 45 (18.1) | 70 (16.5) | 2,610 (10.3) | 2,665 (16.0) | 325 (13.5) | 315 (22.9) | 5,945 (19.6) | 8,620 (20.8) | 1,320 (29.2) | 1,600 (30.8) | 890 (12.0) | 1,525 (10.4) | 250 (28.5) | 155 (17.3) |
| Summer | 430 (14.1) | 1,050 (15.1) | 45 (34.6) | 60 (23.8) | 2,355 (10.6) | 2,020 (17.0) | 260 (14.0) | 210 (24.0) | 5,545 (21.5) | 6,900 (22.7) | 1,065 (33.8) | 1,200 (35.4) | 850 (13.9) | 1,420 (11.3) | 235 (32.5) | 160 (22.8) |
| Autumn | 560 (12.3) | 1,265 (14.4) | 45 (23.7) | 75 (23.5) | 2,160 (11.1) | 2,285 (16.5) | 250 (15.5) | 225 (22.9) | 5,035 (21.6) | 7,865 (21.4) | 1,040 (34.2) | 1,395 (35.1) | 580 (10.9) | 1,190 (11.7) | 165 (28.7) | 120 (22.5) |
| Winter | 990 (11.4) | 2,185 (14.6) | 70 (17.3) | 140 (20.5) | 3,025 (11.0) | 3,635 (17.1) | 395 (16.1) | 415 (24.3) | 7,525 (21.9) | 12,620 (21.2) | 1,880 (36.1) | 2,535 (34.3) | 915 (11.2) | 1,705 (10.9) | 240 (29.2) | 215 (23.9) |
| **Region, N (rate)** |  |  |  |  |  |  |  |  |  |  |  |  |  |  |  |  |
| London | 105 (8.0) | 270 (9.1) | 10 (17.2) | 15 (16.9) | 280 (9.3) | 380 (12.6) | 35 (10.8) | 20 (12.1) | 655 (14.7) | 965 (16.2) | 160 (19.8) | 130 (20.0) | 160 (10.5) | 260 (9.7) | 50 (33.4) | 105 (8.0) |
| North East | 130 (13.2) | 245 (14.9) | SN | 10 (15.5) | 770 (11.6) | 590 (16.4) | 85 (16.3) | 70 (24.1) | 1,695 (21.9) | 1,950 (21.1) | 365 (35.8) | 360 (31.6) | 185 (12.5) | 275 (10.4) | 45 (29.5) | 130 (13.2) |
| North West | 280 (11.5) | 575 (15.5) | 15 (15.0) | 40 (24.8) | 1,095 (9.2) | 1,215 (17.2) | 145 (13.3) | 135 (21.3) | 2,315 (16.8) | 4,100 (21.5) | 570 (28.0) | 810 (31.5) | 345 (11.8) | 565 (10.7) | 80 (24.9) | 280 (11.5) |
| East | 560 (12.0) | 1,455 (13.6) | 40 (20.5) | 80 (20.3) | 2,355 (11.6) | 2,480 (15.8) | 285 (16.3) | 255 (21.9) | 5,460 (22.2) | 8,035 (20.5) | 1,160 (35.0) | 1,455 (32.9) | 670 (10.6) | 1,370 (10.1) | 200 (29.3) | 560 (12.0) |
| West Midlands | 140 (14.3) | 350 (14.4) | 25 (47.6) | 20 (18.7) | 440 (13.1) | 535 (16.8) | 50 (14.9) | 60 (28.2) | 1,075 (25.5) | 1,735 (21.5) | 235 (37.5) | 285 (34.2) | 185 (13.6) | 310 (11.6) | 45 (34.4) | 140 (14.3) |
| Yorkshire and The Humber | 410 (12.6) | 1,145 (14.6) | 55 (24.1) | 60 (17.2) | 1,610 (10.4) | 1,685 (16.9) | 195 (13.7) | 180 (22.7) | 3,900 (20.9) | 6,230 (20.9) | 955 (34.6) | 1,215 (33.6) | 520 (12.3) | 900 (10.3) | 140 (27.5) | 410 (12.6) |
| South East | 195 (13.7) | 320 (16.5) | 10 (17.4) | 20 (21.2) | 565 (9.7) | 665 (17.0) | 90 (17.1) | 85 (26.0) | 1,500 (21.3) | 2,100 (22.0) | 320 (33.9) | 405 (36.6) | 210 (13.0) | 375 (13.2) | 60 (30.3) | 195 (13.7) |
| East Midlands | 500 (12.5) | 1,075 (16.1) | 35 (20.6) | 70 (20.8) | 2,020 (12.2) | 2,015 (18.0) | 230 (15.3) | 240 (27.2) | 4,810 (24.4) | 7,090 (22.6) | 1,065 (37.0) | 1,430 (36.6) | 585 (12.1) | 1,090 (11.2) | 155 (28.7) | 500 (12.5) |
| South West | 275 (12.0) | 440 (16.7) | 10 (13.7) | 20 (24.0) | 1,015 (8.9) | 1,045 (17.1) | 110 (12.9) | 120 (25.5) | 2,640 (19.3) | 3,805 (23.6) | 470 (30.6) | 635 (37.4) | 380 (14.0) | 695 (14.1) | 105 (33.5) | 275 (12.0) |
| **Flu vaccination, N (rate)** |  |  |  |  |  |  |  |  |  |  |  |  |  |  |  |  |
| Yes | 1,415 (21.2) | 3,610 (21.7) | 120 (31.0) | 215 (26.5) | 6,625 (12.9) | 6,955 (22.5) | 820 (19.4) | 800 (29.6) | 15,690 (26.3) | 23,860 (28.9) | 3,575 (42.9) | 4,675 (41.4) | 805 (13.2) | 1,290 (13.7) | 130 (21.3) | 125 (23.2) |
| No | 1,180 (8.1) | 2,270 (9.5) | 85 (14.5) | 130 (15.0) | 3,520 (8.1) | 3,650 (11.1) | 405 (9.9) | 365 (16.3) | 8,365 (15.4) | 12,150 (14.1) | 1,725 (22.7) | 2,050 (23.7) | 2,430 (11.6) | 4,545 (10.4) | 755 (31.6) | 520 (20.9) |
| **Period** |  |  |  |  |  |  |  |  |  |  |  |  |  |  |  |  |
| Pre-pandemic | 1,455 (11.9) | 3,105 (16.2) | 110 (21.0) | 205 (24.3) | 3,770 (11.3) | 4,755 (19.1) | 555 (17.8) | 520 (25.9) | 9,515 (22.9) | 18,085 (24.0) | 2,545 (38.2) | 3,600 (37.9) | 1,420 (11.9) | 2,795 (12.6) | 385 (31.4) | 335 (26.1) |
| During pandemic | 540 (11.8) | 1,260 (14.8) | 50 (21.3) | 75 (18.9) | 2,770 (10.0) | 2,715 (17.3) | 325 (12.8) | 330 (23.8) | 6,345 (19.3) | 8,295 (21.8) | 1,375 (29.4) | 1,635 (31.8) | 785 (10.8) | 1,440 (10.5) | 215 (23.6) | 165 (19.0) |
| After 2^nd^ lockdown | 605 (13.3) | 1,510 (11.7) | 45 (21.1) | 65 (14.9) | 3,605 (10.7) | 3,135 (13.5) | 350 (13.1) | 315 (20.4) | 8,190 (20.8) | 9,625 (17.5) | 1,380 (30.1) | 1,490 (28.1) | 1,035 (13.3) | 1,600 (9.3) | 285 (33.1) | 150 (17.2) |
| ^1^ ABs, antibiotics prescribed or not.  ^2^ Rate is the number of cases per 1000 patients with common infection, calculated by dividing the count of infection-related hospital admission cases (numerator) by the count of infection diagnosis (denominator) and then multiplied by 1000.  ^3^ SN, small numbers, equal or less than 5.  ^4^ BMI, Body Mass Index recorded in the last 5 years.  ^5^ CCI, Charlson Comorbidities Index, measured from 17 weighted conditions, including myocardial infarction, congestive heart failure, peripheral vascular disease, cerebrovascular disease, dementia, chronic pulmonary disease, Connective tissue disease, ulcer disease, mild liver disease, diabetes, hemiplegia, moderate or severe renal disease, diabetes with complications, any malignancy (including leukaemia and lymphoma), moderate or severe liver disease, metastatic solid tumour, and AIDS.  ^6^ IMD, Multiple Deprivation Index, quintile measured from patient-level address. | | | | | | | | | | | | | | | | |
