# Supplementary material for: Evaluation of the impact of COVID-19 pandemic on hospital admission related to common infections: Risk prediction models to tackle antimicrobial resistance in primary care
Source: PLoS One. 2024 Dec 31;19(12):e0311515. doi: 10.1371/journal.pone.0311515 (PMC11687718; doi:10.1371/journal.pone.0311515)
Supplement: S3 Appendix — (DOCX) [file pone.0311515.s003.docx]

# Cox models with overall data

## Performance

All Cox models converged, except the models for prevalent sinusitis with no antibiotics, prevalent sinusitis with antibiotics, and prevalent otitis externa with antibiotics. Extreme values were calculated in hazard ratios (HRs) of some models, i.e., models for incident and prevalent otitis media with antibiotics and models for prevalent URTI with and without antibiotics. C-statistics of the converged models without extreme HRs using development and validation datasets were close; therefore, they were validated.

**S9 Table. C-statistics of Cox models for hospital admissions related to common infections, including lower respiratory tract infection, upper respiratory tract infection, urinary tract infection (UTI), sinusitis, otitis media, and otitis externa, using overall data (from 1 January 2019 to 31 August 2022).**

|  | | | **C-statistics** | |
| --- | --- | --- | --- | --- |
|  |  |  | **Development dataset** | **Validation dataset** |
| LRTI | Incident | No ABs^1^ | 0.67 | 0.67 |
|  |  | With ABs | 0.72 | 0.72 |
|  | Prevalent | No ABs | 0.67 | 0.64 |
|  |  | With ABs | 0.68 | 0.69 |
| URTI | Incident | No ABs | 0.71 | 0.71 |
|  |  | With ABs | 0.71 | 0.71 |
|  | Prevalent | No ABs | 0.71 | 0.70 |
|  |  | With ABs | 0.69 | 0.69 |
| UTI | Incident | No ABs | 0.73 | 0.73 |
|  |  | With ABs | 0.75 | 0.75 |
|  | Prevalent | No ABs | 0.69 | 0.70 |
|  |  | With ABs | 0.69 | 0.69 |
| Sinusitis | Incident | No ABs | 0.71 | 0.70 |
|  |  | With ABs | 0.69 | 0.68 |
|  | Prevalent | No ABs | - | - |
|  |  | With ABs | - | - |
| Otitis media | Incident | No ABs | 0.69 | 0.69 |
|  |  | With ABs | - | - |
|  | Prevalent | No ABs | 0.73 | 0.66 |
|  |  | With ABs | - | - |
| Otitis externa | Incident | No ABs | 0.71 | 0.70 |
|  |  | With ABs | 0.67 | 0.63 |
|  | Prevalent | No ABs | 0.75 | 0.75 |
|  |  | With ABs | - | - |
| URTI components | |  |  |  |
| Specific URTI | Incident | No ABs | 0.76 | 0.74 |
|  |  | With ABs | 0.73 | 0.73 |
|  | Prevalent | No ABs | - | - |
|  |  | With ABs | - | - |
| Cough | Incident | No ABs | 0.71 | 0.71 |
|  |  | With ABs | 0.70 | 0.71 |
|  | Prevalent | No ABs | 0.73 | 0.70 |
|  |  | With ABs | 0.70 | 0.67 |
| Cold with cough | Incident | No ABs | 0.72 | 0.72 |
|  |  | With ABs | 0.72 | 0.72 |
|  | Prevalent | No ABs | 0.72 | 0.72 |
|  |  | With ABs | 0.69 | 0.69 |
| Sore throat | Incident | No ABs | 0.65 | 0.63 |
|  |  | With ABs | 0.62 | 0.61 |
|  | Prevalent | No ABs | 0.65 | 0.60 |
|  |  | With ABs | 0.65 | 0.62 |
| ^1^ABs, antibiotics prescribed or not. | | | | |

## Hazard ratios

**S10 Table. Adjusted hazard ratios of Cox models for hospital admissions related to common infections, including lower respiratory tract infection (LRTI), upper respiratory tract infections (URTI), and urinary tract infection (UTI), using overall data (from 1 January 2019 to 31 August 2022).**

|  | **LRTI, adjusted HR^1^ (95% CI^2^)** | | | **URTI, adjusted HR^1^ (95% CI)** | | | **UTI, adjusted HR^1^ (95% CI)** | | |
| --- | --- | --- | --- | --- | --- | --- | --- | --- | --- |
|  | **Incident** | **Prevalent** | | **Incident** | **Prevalent** | | **Incident** | **Prevalent** | |
|  | **With ABs^1^** | **No ABs** | **With ABs** | **With ABs** | **No ABs** | **With ABs** | **With ABs** | **No ABs** | **With ABs** |
| **Sex** |  |  |  |  |  |  |  |  |  |
| Male | 1.20  (1.17-1.23) | 1.23  (1.15-1.33) | 1.18  (1.11-1.26) | 1.20  (1.18-1.23) | 1.21  (1.15-1.28) | 1.25  (1.19-1.31) | 2.20  (2.15-2.25) | 1.59  (1.49-1.7) | 1.85  (1.76-1.95) |
| **Age** |  |  |  |  |  |  |  |  |  |
| 25-34 | 1.12  (0.99-1.26) | 0.74  (0.51-1.07) | 1.13  (0.79-1.61) | 0.86  (0.81-0.91) | 0.68  (0.59-0.79) | 0.76  (0.64-0.89) | 1.04  (0.95-1.14) | 1.18  (0.9-1.56) | 0.96  (0.78-1.2) |
| 35-44 | 1.06  (0.94-1.19) | 0.73  (0.51-1.04) | 1.01  (0.72-1.42) | 0.76  (0.72-0.81) | 0.4  (0.34-0.48) | 0.58  (0.49-0.69) | 1.06  (0.97-1.16) | 1.09  (0.82-1.45) | 1.01  (0.81-1.24) |
| 45-54 | 1.32  (1.18-1.47) | 0.81  (0.58-1.14) | 1.03  (0.74-1.43) | 0.89  (0.84-0.94) | 0.51  (0.44-0.59) | 0.69  (0.6-0.81) | 1.30  (1.19-1.41) | 1.41  (1.09-1.83) | 1.05  (0.86-1.28) |
| 55-64 | 1.64  (1.47-1.82) | 1.00  (0.72-1.38) | 1.4  (1.01-1.94) | 1.06  (1.01-1.12) | 0.51  (0.44-0.58) | 0.77  (0.67-0.89) | 1.73  (1.6-1.88) | 1.75  (1.37-2.24) | 1.23  (1.02-1.48) |
| 65-74 | 2.49  (2.24-2.76) | 1.60  (1.16-2.20) | 1.71  (1.24-2.37) | 1.56  (1.48-1.64) | 0.76  (0.67-0.87) | 1.08  (0.93-1.24) | 2.57  (2.37-2.77) | 2.45  (1.93-3.11) | 1.67  (1.39-2.0) |
| 75+ | 5.09  (4.59-5.65) | 2.20  (1.61-3.02) | 2.91  (2.12-4.01) | 3.22  (3.06-3.39) | 1.39  (1.22-1.58) | 1.78  (1.55-2.05) | 5.23  (4.84-5.65) | 3.99  (3.16-5.04) | 2.94  (2.45-3.51) |
| **BMI^2^** |  |  |  |  |  |  |  |  |  |
| Underweight | 1.45  (1.36-1.56) | 1.34  (1.11-1.61) | 1.37  (1.16-1.62) | 1.42  (1.34-1.5) | 1.37  (1.17-1.6) | 1.39  (1.21-1.6) | 1.43  (1.34-1.54) | 1.26  (1.02-1.56) | 1.16  (0.98-1.38) |
| Overweight | 0.83  (0.8-0.86) | 0.94  (0.85-1.03) | 0.82  (0.75-0.89) | 0.86  (0.83-0.88) | 0.88  (0.82-0.95) | 0.81  (0.76-0.87) | 0.92  (0.9-0.95) | 0.99  (0.91-1.08) | 0.98  (0.92-1.04) |
| Obese | 0.88  (0.85-0.91) | 0.89  (0.81-0.98) | 0.88  (0.81-0.95) | 0.92  (0.89-0.94) | 0.94  (0.87-1.01) | 0.85  (0.80-0.91) | 1.08  (1.05-1.11) | 1.25  (1.15-1.37) | 1.07  (1.00-1.14) |
| Unknown | 1.03  (0.99-1.07) | 1.05  (0.94-1.18) | 1.01  (0.92-1.12) | 1.09  (1.06-1.12) | 1.24  (1.14-1.35) | 1.02  (0.94-1.11) | 1.11  (1.07-1.15) | 1.23  (1.11-1.35) | 1.14  (1.06-1.23) |
| **Ethnicity** |  |  |  |  |  |  |  |  |  |
| White | 1.09  (1.03-1.16) | 1.00  (0.83-1.21) | 1.23  (1.05-1.45) | 1.15  (1.1-1.2) | 1.05  (0.92-1.19) | 1.30  (1.14-1.48) | 1.08  (1.02-1.15) | 0.95  (0.8-1.13) | 0.95  (0.83-1.09) |
| Unknown | 1.20  (1.13-1.27) | 1.14  (0.94-1.38) | 1.32  (1.12-1.56) | 1.24  (1.19-1.30) | 1.30  (1.14-1.48) | 1.45  (1.27-1.66) | 1.20  (1.13-1.27) | 1.12  (0.95-1.33) | 1.06  (0.93-1.22) |
| **CCI^3^** |  |  |  |  |  |  |  |  |  |
| Low | 1.22  (1.19-1.26) | 1.21  (1.11-1.32) | 1.18  (1.1-1.27) | 1.22  (1.19-1.25) | 1.43  (1.34-1.52) | 1.19  (1.12-1.26) | 1.37  (1.34-1.41) | 1.35  (1.26-1.46) | 1.38  (1.3-1.45) |
| Medium | 1.62  (1.56-1.68) | 1.50  (1.35-1.68) | 1.58  (1.44-1.73) | 1.66  (1.61-1.71) | 1.91  (1.76-2.08) | 1.63  (1.51-1.76) | 1.67  (1.62-1.73) | 1.68  (1.53-1.85) | 1.65  (1.54-1.78) |
| High | 2.05  (1.92-2.18) | 1.69  (1.42-2.02) | 1.84  (1.58-2.14) | 2.05  (1.94-2.16) | 2.0  (1.73-2.31) | 2.03  (1.80-2.30) | 2.00  (1.89-2.11) | 1.82  (1.57-2.11) | 2.07  (1.85-2.33) |
| Very high | 2.46  (2.21-2.73) | 2.08  (1.59-2.71) | 2.88  (2.33-3.56) | 2.58  (2.37-2.81) | 2.34  (1.85-2.95) | 2.80  (2.31-3.39) | 2.32  (2.11-2.56) | 1.93  (1.51-2.48) | 2.12  (1.74-2.59) |
| **Smoking** |  |  |  |  |  |  |  |  |  |
| Smoker | 0.88  (0.84-0.91) | 0.97  (0.86-1.10) | 0.86  (0.78-0.94) | 0.89  (0.87-0.92) | 0.88  (0.81-0.95) | 0.80  (0.74-0.86) | 1.21  (1.17-1.26) | 1.24  (1.11-1.39) | 1.19  (1.09-1.29) |
| Never smoked | 0.98  (0.96-1.01) | 0.87  (0.80-0.94) | 0.95  (0.89-1.02) | 0.96  (0.94-0.99) | 0.90  (0.85-0.96) | 0.96  (0.91-1.02) | 0.94  (0.92-0.96) | 0.95  (0.89-1.02) | 0.95  (0.90-1.00) |
| Unknown | 1.10  (0.85-1.43) | 0.95  (0.45-2.02) | 1.46  (0.76-2.83) | 0.99  (0.85-1.14) | 1.23  (0.86-1.76) | 1.27  (0.86-1.88) | 1.15  (0.92-1.43) | 0.57  (0.24-1.38) | 0.46  (0.21-1.03) |
| **IMD^4^** |  |  |  |  |  |  |  |  |  |
| 1 (most deprived) | 1.05  (1.02-1.10) | 1.05  (0.94-1.17) | 1.05  (0.96-1.15) | 1.01  (0.98-1.04) | 1.03  (0.95-1.12) | 1.01  (0.94-1.09) | 1.08  (1.05-1.12) | 1.06  (0.96-1.17) | 1.01  (0.94-1.09) |
| 3 | 0.98  (0.94-1.01) | 0.98  (0.88-1.10) | 0.99  (0.9-1.08) | 0.97  (0.94-1.00) | 0.89  (0.82-0.96) | 0.97  (0.90-1.04) | 0.94  (0.91-0.97) | 0.88  (0.8-0.96) | 0.87  (0.81-0.93) |
| 4 | 0.99  (0.95-1.03) | 0.95  (0.85-1.06) | 0.96  (0.88-1.06) | 1.00  (0.97-1.03) | 0.98  (0.90-1.06) | 0.95  (0.88-1.03) | 0.88  (0.85-0.91) | 0.79  (0.71-0.87) | 0.84  (0.78-0.9) |
| 5 (most affluent) | 0.93  (0.90-0.97) | 0.91  (0.81-1.03) | 0.92  (0.83-1.01) | 0.93  (0.9-0.96) | 0.83  (0.76-0.91) | 0.94  (0.87-1.02) | 0.82  (0.79-0.85) | 0.73  (0.66-0.80) | 0.75  (0.69-0.80) |
| Unknown | 0.98  (0.89-1.08) | 1.19  (0.91-1.57) | 1.07  (0.85-1.33) | 0.97  (0.9-1.05) | 1.14  (0.93-1.39) | 1.00  (0.83-1.21) | 0.98  (0.89-1.06) | 1.02  (0.79-1.31) | 0.92  (0.77-1.11) |
| **Season** |  |  |  |  |  |  |  |  |  |
| Spring | 0.94  (0.90-0.97) | 0.88  (0.79-0.98) | 0.88  (0.81-0.96) | 0.94  (0.91-0.96) | 0.94  (0.87-1.02) | 0.86  (0.80-0.93) | 0.99  (0.96-1.02) | 0.98  (0.9-1.07) | 0.92  (0.86-0.98) |
| Summer | 1.03  (0.99-1.07) | 0.99  (0.89-1.11) | 0.89  (0.81-0.98) | 1.0  (0.97-1.03) | 1.03  (0.95-1.11) | 0.95  (0.88-1.03) | 1.01  (0.98-1.04) | 1.02  (0.93-1.11) | 0.96  (0.9-1.03) |
| Winter | 0.96  (0.93-0.99) | 0.97  (0.88-1.07) | 0.91  (0.84-0.98) | 0.98  (0.95-1.0) | 1.09  (1.01-1.17) | 0.95  (0.89-1.02) | 0.98  (0.95-1.01) | 0.96  (0.88-1.05) | 0.91  (0.85-0.97) |
| **Region** |  |  |  |  |  |  |  |  |  |
| London | 0.95  (0.87-1.03) | 1.05  (0.82-1.33) | 0.99  (0.77-1.27) | 0.93  (0.88-0.98) | 0.86  (0.73-1.01) | 0.81  (0.67-0.97) | 1.04  (0.97-1.11) | 1.11  (0.93-1.33) | 0.96  (0.8-1.15) |
| North East | 0.96  (0.91-1.02) | 1.03  (0.88-1.21) | 0.86  (0.74-0.99) | 1.02  (0.98-1.07) | 1.00 (0.88-1.12) | 0.98  (0.87-1.11) | 1.08  (1.02-1.13) | 1.01  (0.87-1.17) | 1.05  (0.94-1.19) |
| North West | 0.96  (0.92-1.00) | 0.88  (0.77-1.00) | 0.93  (0.84-1.03) | 1.03  (0.99-1.06) | 0.79  (0.72-0.87) | 0.98  (0.90-1.07) | 0.97  (0.93-1.01) | 0.78  (0.70-0.88) | 0.97  (0.89-1.05) |
| West Midlands | 1.05  (0.99-1.12) | 1.05  (0.87-1.27) | 0.92  (0.79-1.08) | 1.08  (1.03-1.13) | 1.10  (0.96-1.26) | 1.10  (0.96-1.25) | 1.01  (0.95-1.07) | 1.21  (1.03-1.42) | 1.12  (0.99-1.26) |
| Yorkshire and The Humber | 0.96  (0.93-1.00) | 0.94  (0.84-1.05) | 0.96  (0.88-1.05) | 1.03  (1.00-1.06) | 0.96  (0.88-1.04) | 1.0  (0.93-1.09) | 1.05  (1.02-1.09) | 0.95  (0.86-1.05) | 1.08  (1.01-1.16) |
| South East | 0.98  (0.93-1.04) | 0.91  (0.77-1.08) | 0.93  (0.81-1.08) | 1.01  (0.96-1.05) | 0.96  (0.86-1.08) | 1.03  (0.92-1.15) | 0.93  (0.88-0.97) | 0.80  (0.69-0.93) | 0.95  (0.85-1.06) |
| East Midlands | 1.07  (1.03-1.11) | 1.06  (0.95-1.19) | 1.03  (0.94-1.12) | 1.10  (1.07-1.14) | 1.07  (0.99-1.16) | 1.12  (1.04-1.20) | 0.99  (0.96-1.02) | 1.03  (0.94-1.13) | 1.01  (0.95-1.09) |
| South West | 1.05  (1.01-1.1) | 1.01  (0.89-1.16) | 1.08  (0.96-1.21) | 1.07  (1.03-1.11) | 0.88  (0.80-0.98) | 1.12  (1.02-1.23) | 0.91  (0.87-0.94) | 0.88  (0.79-0.99) | 0.90  (0.82-0.98) |
| **Flu vaccination** |  |  |  |  |  |  |  |  |  |
| Yes | 0.97  (0.94-1.00) | 0.98  (0.90-1.07) | 0.95  (0.88-1.01) | 0.98  (0.96-1.00) | 0.91  (0.85-0.97) | 0.98  (0.92-1.04) | 0.96  (0.93-0.98) | 0.95  (0.88-1.02) | 1.00  (0.95-1.06) |
| **Count of antibiotic prescription in the one year before** | 1.10  (1.09-1.10) | 1.05  (1.04-1.06) | 1.09  (1.07-1.10) | 1.12  (1.12-1.13) | 1.14  (1.13-1.15) | 1.11  (1.10-1.12) | 1.05  (1.04-1.05) | 1.03  (1.02-1.04) | 1.04  (1.03-1.04) |
| ^1^ HR, hazard ratio.  ^2^ CI, confidence interval.  ^3^ ABs, antibiotics prescribed or not.  ^4^ BMI, Body Mass Index recorded in the last 5 years.  ^5^ CCI, Charlson Comorbidities Index, measured from 17 weighted conditions, including myocardial infarction, congestive heart failure, peripheral vascular disease, cerebrovascular disease, dementia, chronic pulmonary disease, Connective tissue disease, ulcer disease, mild liver disease, diabetes, hemiplegia, moderate or severe renal disease, diabetes with complications, any malignancy (including leukaemia and lymphoma), moderate or severe liver disease, metastatic solid tumour, and AIDS.  ^6^ IMD, Multiple Deprivation Index, quintile measured from patient-level address.  Reference group for variable sex is female, for age is 18-25, for BMI is healthy weight, for ethnicity is non-white, for CCI is very low, for smoking status is ex-smoker, for IMD is 2, for season is autumn, for region is east, for flu vaccination is no. | | | | | | | | | |

**S11 Table. Adjusted hazard ratios of Cox models for hospital admissions related to other common infections, including sinusitis, otitis media, and otitis externa, using overall data (from 1 January 2019 to 31 August 2022).**

|  | **Sinusitis, adjusted HR^1^**  **(95% CI^2^)** | | **Otitis media, adjusted HR (95% CI)** | | **Otitis externa, adjusted HR**  **(95% CI)** | | |
| --- | --- | --- | --- | --- | --- | --- | --- |
|  | **Incident** | | **Incident** | **Prevalent** | **Incident** | | **Prevalent** |
|  | **No ABs^3^** | **With ABs** | **No ABs** | **No ABs** | **No ABs** | **With ABs** | **No ABs** |
| **Sex** |  |  |  |  |  |  |  |
| Male | 1.40 (1.14-1.72) | 1.28  (1.10-1.48) | 1.29 (1.08-1.54) | 0.96 (0.64-1.43) | 1.18 (1.08-1.28) | 1.13 (1.0-1.27) | 1.40 (1.20-1.63) |
| **Age** |  |  |  |  |  |  |  |
| 25-34 | 0.94  (0.60-1.49) | 0.79  (0.57-1.08) | 1.24 (0.83-1.86) | 1.01 (0.40-2.51) | 0.92 (0.76-1.13) | 1.03 (0.79-1.34) | 0.87 (0.57-1.32) |
| 35-44 | 0.79  (0.50-1.27) | 0.59  (0.43-0.82) | 1.20 (0.79-1.82) | 1.48 (0.61-3.56) | 0.79 (0.64-0.97) | 0.82 (0.63-1.08) | 1.13 (0.75-1.7) |
| 45-54 | 0.80  (0.50-1.27) | 0.53  (0.38-0.73) | 1.14 (0.76-1.73) | 0.93 (0.36-2.41) | 0.79 (0.65-0.97) | 0.82 (0.63-1.07) | 0.81 (0.53-1.22) |
| 55-64 | 0.88  (0.55-1.40) | 0.68  (0.49-0.94) | 1.33 (0.89-2.01) | 0.54 (0.18-1.60) | 0.71 (0.58-0.88) | 0.76 (0.58-1.00) | 0.84 (0.55-1.27) |
| 65-74 | 0.89  (0.54-1.48) | 0.80 (0.57-1.12) | 1.52 (1-2.32) | 1.42 (0.53-3.82) | 1.00 (0.81-1.23) | 0.98 (0.74-1.31) | 0.97 (0.64-1.48) |
| 75+ | 2.55  (1.55-4.18) | 1.72  (1.22-2.43) | 2.71 (1.79-4.1) | 4.00 (1.57-10.18) | 2.13 (1.74-2.61) | 2.25 (1.71-2.96) | 3.07 (2.07-4.56) |
| **BMI**^4^ |  |  |  |  |  |  |  |
| Underweight | 1.47  (0.71-3.04) | 1.12  (0.65-1.93) | 1.66 (0.90-3.10) | 3.03 (0.88-10.38) | 1.30 (0.92-1.84) | 0.77 (0.43-1.38) | 0.64 (0.26-1.56) |
| Overweight | 0.80  (0.59-1.07) | 0.92  (0.76-1.11) | 1.18 (0.92-1.52) | 0.76 (0.41-1.39) | 1.01 (0.89-1.14) | 0.93 (0.79-1.10) | 1.21 (0.98-1.49) |
| Obese | 1.14  (0.87-1.51) | 1.14  (0.95-1.37) | 1.10 (0.85-1.4) | 1.26 (0.73-2.20) | 1.22 (1.08-1.38) | 1.07 (0.91-1.25) | 1.23 (0.99-1.52) |
| Unknown | 1.21  (0.89-1.62) | 1.06  (0.86-1.30) | 1.27 (0.97-1.65) | 1.43 (0.79-2.59) | 1.13 (0.98-1.29) | 0.81 (0.67-0.97) | 0.91 (0.69-1.18) |
| **Ethnicity** |  |  |  |  |  |  |  |
| White | 0.71  (0.49-1.03) | 0.84  (0.64-1.11) | 1.18 (0.83-1.68) | 0.40 (0.22-0.73) | 1.01 (0.85-1.21) | 1.02 (0.80-1.30) | 0.97 (0.70-1.34) |
| Unknown | 0.84  (0.57-1.24) | 0.87  (0.66-1.16) | 1.12 (0.78-1.62) | 0.47 (0.25-0.89) | 0.99 (0.82-1.19) | 1.10 (0.86-1.41) | 1.06 (0.76-1.48) |
| **CCI**^5^ |  |  |  |  |  |  |  |
| Low | 1.26  (1.00-1.60) | 1.38  (1.19-1.62) | 1.41 (1.15-1.72) | 1.26 (0.79-2.01) | 1.48 (1.34-1.64) | 1.27 (1.11-1.45) | 1.43 (1.19-1.71) |
| Medium | 1.84  (1.23-2.76) | 2.21  (1.71-2.85) | 1.98 (1.45-2.72) | 1.96 (0.94-4.10) | 2.25 (1.94-2.61) | 2.12 (1.74-2.59) | 2.68 (2.13-3.38) |
| High | 2.45  (1.19-5.05) | 2.67  (1.58-4.51) | 2.67 (1.54-4.66) | 1.78 (0.42-7.61) | 3.48 (2.73-4.42) | 2.49 (1.73-3.58) | 3.81 (2.7-5.38) |
| Very high | 1.47  (0.21-10.54) | 5.49  (2.82-10.68) | 0.67 (0.09-4.81) | 5.35 (1.23-23.10) | 6.13 (4.31-8.71) | 3.48 (1.91-6.36) | 3.83 (1.96-7.48) |
| **Smoking** |  |  |  |  |  |  |  |
| Smoker | 1.38  (1.04-1.83) | 1.18  (0.97-1.43) | 1.27 (0.99-1.62) | 1.50 (0.86-2.64) | 1.16 (1.02-1.31) | 0.99 (0.84-1.17) | 1.02 (0.80-1.29) |
| Never smoked | 0.93  (0.75-1.17) | 1.03  (0.89-1.19) | 1.02 (0.84-1.23) | 1.04 (0.66-1.63) | 0.89 (0.81-0.99) | 1.01 (0.89-1.15) | 0.95 (0.80-1.12) |
| Unknown | 1.47  (0.45-4.77) | 1.40  (0.56-3.47) | 1.11 (0.40-3.1) | 2.05 (0.26-16.12) | 0.45 (0.20-1.02) | 0.60 (0.22-1.64) | 0.79 (0.19-3.27) |
| **IMD**^6^ |  |  |  |  |  |  |  |
| 1 (most deprived) | 1.23  (0.90-1.68) | 1.03  (0.83-1.27) | 1.06 (0.82-1.38) | 0.64 (0.36-1.14) | 1.00 (0.88-1.14) | 0.95 (0.80-1.12) | 1.25 (0.99-1.58) |
| 3 | 1.05  (0.76-1.45) | 1.12  (0.91-1.37) | 0.95 (0.72-1.25) | 1.18 (0.69-2.01) | 1.02 (0.89-1.16) | 0.95 (0.80-1.14) | 0.96 (0.75-1.22) |
| 4 | 0.97  (0.70-1.36) | 0.91  (0.73-1.13) | 1.16 (0.89-1.51) | 0.44 (0.21-0.90) | 0.94 (0.82-1.08) | 0.89 (0.75-1.07) | 1.09 (0.86-1.38) |
| 5 (most affluent) | 1.06  (0.76-1.48) | 1.03  (0.83-1.28) | 1.16 (0.88-1.52) | 0.70 (0.37-1.32) | 0.91 (0.79-1.05) | 0.89 (0.73-1.07) | 0.89 (0.69-1.14) |
| Unknown | 1.43  (0.71-2.88) | 1.02  (0.61-1.71) | 1.06 (0.53-2.1) | 0.30 (0.04-2.29) | 0.94 (0.66-1.34) | 0.83 (0.52-1.33) | 1.01 (0.53-1.93) |
| **Season** |  |  |  |  |  |  |  |
| Spring | 1.26  (0.93-1.71) | 0.90 (0.74-1.09) | 0.88 (0.69-1.13) | 0.83 (0.46-1.52) | 0.91 (0.81-1.02) | 0.82 (0.70-0.97) | 0.72 (0.59-0.88) |
| Summer | 1.45  (1.07-1.98) | 0.94  (0.77-1.15) | 0.92 (0.73-1.16) | 1.19 (0.68-2.08) | 0.95 (0.85-1.07) | 0.91 (0.78-1.06) | 0.77 (0.64-0.93) |
| Winter | 1.22  (0.91-1.64) | 0.98  (0.82-1.17) | 0.90 (0.71-1.14) | 1.11 (0.65-1.90) | 0.83 (0.74-0.94) | 0.84 (0.72-0.98) | 0.65 (0.53-0.8) |
| **Region** |  |  |  |  |  |  |  |
| London | 1.11  (0.66-1.86) | 1.11  (0.74-1.66) | 1.20 (0.74-1.93) | 0.95 (0.34-2.69) | 1.45 (1.15-1.82) | 1.05 (0.74-1.5) | 1.45 (0.94-2.25) |
| North East | 1.35  (0.87-2.08) | 1.01  (0.70-1.45) | 1.40 (0.97-2.03) | 2.04 (0.89-4.71) | 1.27 (1.03-1.56) | 1.60 (1.23-2.07) | 1.53 (1.08-2.18) |
| North West | 0.94  (0.65-1.36) | 1.29  (1.02-1.64) | 1.21 (0.89-1.65) | 0.88 (0.39-2.03) | 1.29 (1.10-1.52) | 1.12 (0.89-1.41) | 1.21 (0.91-1.62) |
| West Midlands | 1.19  (0.70-2.00) | 1.55  (1.11-2.16) | 1.29 (0.83-2.03) | 1.30 (0.48-3.53) | 1.22 (0.96-1.57) | 1.38 (1.00-1.90) | 1.29 (0.79-2.09) |
| Yorkshire and The Humber | 1.06  (0.77-1.46) | 1.37  (1.11-1.68) | 1.22 (0.92-1.62) | 1.81 (0.99-3.29) | 1.33 (1.16-1.54) | 1.59 (1.32-1.91) | 1.48 (1.15-1.91) |
| South East | 0.81  (0.50-1.31) | 1.17  (0.87-1.57) | 1.27 (0.85-1.90) | 0.89 (0.30-2.64) | 1.22 (1.00-1.48) | 1.18 (0.90-1.55) | 1.30 (0.93-1.82) |
| East Midlands | 1.12  (0.81-1.53) | 1.25  (1.03-1.53) | 1.69 (1.32-2.18) | 1.59 (0.86-2.92) | 1.54 (1.34-1.76) | 1.61 (1.36-1.92) | 1.89 (1.50-2.40) |
| South West | 1.03  (0.71-1.49) | 1.15  (0.9-1.48) | 1.31 (0.94-1.82) | 1.98 (0.92-4.26) | 1.36 (1.17-1.59) | 1.72 (1.40-2.11) | 1.45 (1.10-1.92) |
| **Flu vaccination** |  |  |  |  |  |  |  |
| Yes | 1.26  (0.97-1.62) | 1.14  (0.97-1.34) | 1.14 (0.91-1.40) | 1.39 (0.83-2.32) | 1.05 (0.95-1.17) | 1.12 (0.97-1.29) | 1.03 (0.85-1.25) |
| **Count of antibiotic prescription in the one year before** | 1.26  (1.21-1.32) | 1.22  (1.19-1.26) | 1.23 (1.17-1.28) | 1.06 (0.96-1.19) | 1.29 (1.26-1.32) | 1.14 (1.10-1.18) | 1.21 (1.16-1.26) |
| ^1^ HR, hazard ratio.  ^2^ CI, confidence interval.  ^3^ ABs, antibiotics prescribed or not.  ^4^ BMI, Body Mass Index recorded in the last 5 years.  ^5^ CCI, Charlson Comorbidities Index, measured from 17 weighted conditions, including myocardial infarction, congestive heart failure, peripheral vascular disease, cerebrovascular disease, dementia, chronic pulmonary disease, Connective tissue disease, ulcer disease, mild liver disease, diabetes, hemiplegia, moderate or severe renal disease, diabetes with complications, any malignancy (including leukaemia and lymphoma), moderate or severe liver disease, metastatic solid tumour, and AIDS.  ^6^ IMD, Multiple Deprivation Index, quintile measured from patient-level address.  Reference group for variable sex is female, for age is 18-25, for BMI is healthy weight, for ethnicity is non-white, for CCI is very low, for smoking status is ex-smoker, for IMD is 2, for season is autumn, for region is east, for flu vaccination is no. | | | | | | | |

**S12 Table. Adjusted hazard ratios of Cox models for hospital admissions related to the components of upper respiratory tract infections (URTI), including specific URTI, cough, cold with cough, and sore throat, using overall data (from 1 January 2019 to 31 August 2022).**

|  | **URTI, adjusted HR^1^**  **(95% CI^2^)** | | **Cough, adjusted HR (95% CI)** | | | | **Cold with cough, adjusted HR (95% CI)** | | | | **Sore throat, adjusted HR (95% CI)** | | | |
| --- | --- | --- | --- | --- | --- | --- | --- | --- | --- | --- | --- | --- | --- | --- |
|  | **Incident** | | **Incident** | | **Prevalent** | | **Incident** | | **Prevalent** | | **Incident** | | **Prevalent** | |
|  | **With ABs^3^** | **No ABs** | **With ABs** | **No ABs** | **With ABs** | **No ABs** | **With ABs** | **No ABs** | **With ABs** | **No ABs** | **With ABs** | **No ABs** | **With ABs** | **No ABs** |
| **Sex** |  |  |  |  |  |  |  |  |  |  |  |  |  |  |
| Male | 1.25  (1.14-1.38) | 1.20  (1.13-1.28) | 1.18  (1.13-1.24) | 1.17  (1.12-1.23) | 1.22  (1.07-1.4) | 1.27  (1.11-1.45) | 1.15  (1.12-1.19) | 1.17  (1.14-1.19) | 1.22  (1.14-1.3) | 1.24  (1.17-1.31) | 1.56  (1.43-1.7) | 1.44  (1.34-1.53) | 1.40  (1.18-1.66) | 1.30  (1.05-1.59) |
| **Age** |  |  |  |  |  |  |  |  |  |  |  |  |  |  |
| 25-34 | 1.03  (0.8-1.32) | 0.86  (0.69-1.07) | 1.08  (0.88-1.32) | 1.22  (1.00-1.49) | 1.50  (0.76-2.96) | 1.21  (0.63-2.31) | 1.24  (1.08-1.44) | 1.23  (1.09-1.39) | 1.26  (0.89-1.78) | 1.43  (1.01-2.03) | 0.71  (0.63-0.8) | 0.73  (0.68-0.8) | 0.70  (0.58-0.85) | 0.59  (0.47-0.74) |
| 35-44 | 1.12  (0.87-1.43) | 0.89  (0.72-1.10) | 1.08  (0.88-1.31) | 1.12  (0.92-1.37) | 0.98  (0.5-1.95) | 0.88  (0.46-1.69) | 1.39  (1.21-1.6) | 1.18  (1.05-1.32) | 1.05  (0.75-1.48) | 1.17  (0.83-1.65) | 0.6  (0.52-0.69) | 0.64  (0.58-0.71) | 0.53  (0.41-0.67) | 0.42  (0.30-0.57) |
| 45-54 | 1.13  (0.88-1.45) | 1.08  (0.88-1.32) | 1.28  (1.06-1.54) | 1.38  (1.14-1.66) | 1.63  (0.87-3.07) | 1.20  (0.66-2.21) | 1.53  (1.34-1.75) | 1.39  (1.24-1.55) | 1.50  (1.09-2.07) | 1.45  (1.04-2.01) | 0.50  (0.43-0.59) | 0.62  (0.56-0.7) | 0.38  (0.28-0.52) | 0.39  (0.26-0.58) |
| 55-64 | 1.50  (1.18-1.91) | 1.35  (1.11-1.65) | 1.42  (1.19-1.7) | 1.54  (1.28-1.85) | 1.47  (0.79-2.75) | 0.94  (0.51-1.73) | 1.92  (1.69-2.18) | 1.70  (1.53-1.90) | 1.61  (1.18-2.21) | 1.68  (1.22-2.32) | 0.57  (0.48-0.67) | 0.66  (0.58-0.76) | 0.41  (0.28-0.59) | 0.44  (0.28-0.71) |
| 65-74 | 2.51  (1.98-3.19) | 1.83  (1.51-2.23) | 1.96  (1.64-2.34) | 2.09  (1.75-2.51) | 1.92  (1.03-3.59) | 1.65  (0.91-2.99) | 2.88  (2.54-3.27) | 2.52  (2.26-2.80) | 2.39  (1.74-3.27) | 2.18  (1.58-3.00) | 0.65  (0.54-0.78) | 0.82  (0.70-0.96) | 0.29  (0.17-0.49) | 0.95  (0.60-1.5) |
| 75+ | 4.77  (3.79-6.02) | 4.04  (3.34-4.90) | 3.81  (3.19-4.54) | 4.17  (3.49-5.00) | 3.47  (1.86-6.44) | 2.30  (1.27-4.16) | 6.17  (5.45-6.99) | 5.07  (4.56-5.63) | 4.22  (3.09-5.76) | 3.64  (2.65-5.01) | 1.26  (1.05-1.51) | 1.79  (1.54-2.07) | 0.65  (0.4-1.07) | 0.89  (0.50-1.58) |
| **BMI^4^** |  |  |  |  |  |  |  |  |  |  |  |  |  |  |
| Underweight | 1.30  (0.97-1.75) | 1.62  (1.36-1.93) | 1.53  (1.35-1.73) | 1.36  (1.19-1.56) | 1.45  (0.95-2.19) | 1.34  (0.89-2.02) | 1.40  (1.29-1.52) | 1.39  (1.29-1.49) | 1.44  (1.21-1.73) | 1.26  (1.07-1.49) | 1.31  (0.98-1.74) | 1.09  (0.87-1.37) | 1.44  (0.88-2.36) | 0.80  (0.39-1.65) |
| Overweight | 0.92  (0.81-1.05) | 0.86  (0.79-0.94) | 0.84  (0.79-0.89) | 0.83  (0.78-0.89) | 0.84  (0.70-1.01) | 0.77  (0.64-0.94) | 0.85  (0.82-0.89) | 0.85  (0.82-0.88) | 0.83  (0.76-0.91) | 0.80  (0.74-0.86) | 1.01  (0.89-1.14) | 0.91  (0.83-1.00) | 1.00  (0.78-1.28) | 0.88  (0.66-1.18) |
| Obese | 0.92  (0.81-1.05) | 0.91  (0.84-0.99) | 0.95  (0.90-1.01) | 0.91  (0.86-0.97) | 1.07  (0.90-1.28) | 0.92  (0.77-1.10) | 0.97  (0.93-1.01) | 0.89  (0.86-0.92) | 0.94  (0.86-1.02) | 0.84  (0.78-0.9) | 1.20  (1.06-1.35) | 0.97  (0.89-1.07) | 1.25  (0.99-1.58) | 1.06  (0.81-1.39) |
| Unknown | 1.08  (0.93-1.24) | 0.98  (0.89-1.09) | 1.28  (1.18-1.38) | 1.08  (1.01-1.17) | 1.24  (0.98-1.55) | 1.08  (0.85-1.36) | 1.40  (1.33-1.47) | 1.06  (1.02-1.11) | 1.22  (1.10-1.36) | 0.97  (0.88-1.06) | 1.25  (1.11-1.4) | 0.99  (0.91-1.08) | 1.16  (0.93-1.44) | 1.05  (0.81-1.36) |
| **Ethnicity** |  |  |  |  |  |  |  |  |  |  |  |  |  |  |
| White | 1.04  (0.86-1.25) | 1.17  (1.04-1.32) | 0.97  (0.86-1.08) | 1.14  (1.02-1.26) | 1.18  (0.84-1.65) | 1.14  (0.82-1.59) | 1.04  (0.97-1.12) | 1.13  (1.07-1.20) | 1.03  (0.88-1.20) | 1.23  (1.06-1.44) | 1.17  (1.01-1.36) | 1.11  (0.99-1.25) | 0.95  (0.71-1.26) | 0.92  (0.64-1.33) |
| Unknown | 1.23  (1.01-1.48) | 1.24  (1.10-1.40) | 1.12  (1.0-1.26) | 1.27  (1.14-1.42) | 1.56  (1.10-2.21) | 1.29  (0.92-1.82) | 1.19  (1.1-1.28) | 1.23  (1.16-1.31) | 1.22  (1.04-1.44) | 1.37  (1.17-1.6) | 1.12  (0.96-1.3) | 1.14  (1.01-1.28) | 1.03  (0.77-1.38) | 1.03  (0.71-1.49) |
| **CCI^5^** |  |  |  |  |  |  |  |  |  |  |  |  |  |  |
| Low | 1.57  (1.41-1.75) | 1.26  (1.17-1.35) | 1.32  (1.25-1.39) | 1.22  (1.15-1.28) | 1.47  (1.26-1.72) | 1.38  (1.17-1.62) | 1.33  (1.29-1.38) | 1.23  (1.20-1.27) | 1.4  (1.29-1.51) | 1.25  (1.17-1.34) | 1.22  (1.10-1.35) | 1.17  (1.09-1.27) | 1.43  (1.17-1.73) | 1.19  (0.95-1.50) |
| Medium | 2.09  (1.79-2.45) | 1.63  (1.47-1.8) | 1.84  (1.71-1.98) | 1.61  (1.5-1.73) | 1.80  (1.46-2.23) | 1.77  (1.44-2.19) | 1.80  (1.71-1.88) | 1.66  (1.60-1.73) | 1.90  (1.73-2.1) | 1.67  (1.53-1.82) | 1.65  (1.35-2.03) | 1.69  (1.44-2.00) | 0.83  (0.4-1.7) | 2.12  (1.27-3.55) |
| High | 3.43  (2.7-4.34) | 2.25  (1.91-2.64) | 2.55  (2.26-2.88) | 2.14  (1.90-2.42) | 1.99  (1.39-2.85) | 2.35  (1.69-3.26) | 2.37  (2.20-2.56) | 2.11  (1.98-2.25) | 1.99  (1.69-2.35) | 2.17  (1.89-2.49) | 2.22  (1.55-3.18) | 2.03  (1.48-2.78) | 1.66  (0.61-4.52) | 1.39  (0.44-4.45) |
| Very high | 4.23  (2.89-6.20) | 2.82  (2.15-3.70) | 3.05  (2.48-3.75) | 2.8  (2.31-3.39) | 2.21  (1.21-4.06) | 2.81  (1.63-4.84) | 2.98  (2.64-3.38) | 2.56  (2.31-2.84) | 2.31  (1.78-3.00) | 2.66  (2.14-3.31) | 3.58  (2.18-5.89) | 3.01  (1.89-4.81) | 3.08  (0.76-12.5) | 1.5  (0.21-10.83) |
| **Smoking** |  |  |  |  |  |  |  |  |  |  |  |  |  |  |
| Smoker | 1.24  (1.08-1.43) | 0.84  (0.76-0.93) | 0.88  (0.82-0.94) | 0.91  (0.85-0.97) | 0.75  (0.61-0.93) | 0.64  (0.52-0.80) | 0.85  (0.81-0.88) | 0.87  (0.83-0.90) | 0.92  (0.84-1.02) | 0.83  (0.76-0.91) | 1.37  (1.22-1.53) | 1.15  (1.06-1.25) | 1.13  (0.92-1.40) | 1.02  (0.79-1.32) |
| Never smoked | 0.89  (0.81-0.99) | 0.90  (0.84-0.96) | 1.16  (1.09-1.23) | 0.99  (0.94-1.04) | 0.93  (0.79-1.08) | 1.00  (0.85-1.17) | 1.19  (1.15-1.24) | 1.01  (0.98-1.04) | 0.94  (0.87-1.02) | 1.00  (0.94-1.07) | 0.92  (0.84-1.02) | 0.90  (0.84-0.96) | 0.82  (0.68-0.98) | 0.87  (0.7-1.07) |
| Unknown | 0.57  (0.25-1.28) | 1.07  (0.63-1.83) | 0.56  (0.27-1.19) | 1.40  (0.89-2.22) | 1.05  (0.14-7.57) | 1.30  (0.18-9.42) | 0.85  (0.59-1.23) | 1.08  (0.83-1.41) | 1.23  (0.58-2.60) | 1.60  (0.86-3.00) | 1.24  (0.96-1.60) | 0.84  (0.68-1.03) | 0.99  (0.62-1.59) | 0.90  (0.52-1.58) |
| **IMD^6^** |  |  |  |  |  |  |  |  |  |  |  |  |  |  |
| 1 (most deprived) | 1.08  (0.94-1.24) | 1.15  (1.05-1.25) | 1.07  (1.00-1.14) | 1.03  (0.97-1.10) | 1.11  (0.90-1.35) | 0.96  (0.78-1.17) | 1.09  (1.04-1.14) | 1.03  (0.99-1.06) | 1.09  (0.99-1.20) | 1.02  (0.93-1.11) | 0.90  (0.79-1.01) | 0.98  (0.89-1.07) | 1.17  (0.93-1.49) | 0.85  (0.64-1.13) |
| 3 | 0.88  (0.76-1.02) | 1.00  (0.91-1.10) | 0.93  (0.87-1.00) | 0.95  (0.88-1.01) | 0.9  (0.73-1.10) | 0.95  (0.77-1.16) | 0.97  (0.92-1.01) | 0.97  (0.93-1.01) | 0.89  (0.80-0.98) | 0.99  (0.91-1.08) | 1.00  (0.88-1.13) | 1.04  (0.95-1.15) | 1.08  (0.85-1.38) | 1.03  (0.78-1.35) |
| 4 | 1.02  (0.88-1.17) | 1.00  (0.91-1.11) | 0.90  (0.84-0.97) | 1.04  (0.97-1.11) | 1.13  (0.92-1.38) | 1.00  (0.81-1.23) | 0.97  (0.92-1.01) | 1.00  (0.96-1.04) | 0.98  (0.88-1.08) | 0.97  (0.88-1.06) | 0.92  (0.81-1.05) | 1.01  (0.92-1.12) | 0.95  (0.73-1.23) | 0.92  (0.69-1.23) |
| 5 (most affluent) | 0.90  (0.78-1.05) | 0.96  (0.87-1.07) | 0.84  (0.77-0.91) | 0.91  (0.84-0.98) | 0.75  (0.60-0.95) | 0.84  (0.67-1.06) | 0.89  (0.84-0.93) | 0.92  (0.88-0.96) | 0.87  (0.78-0.97) | 0.93  (0.85-1.03) | 0.92  (0.80-1.05) | 1.05  (0.95-1.16) | 1.14  (0.88-1.47) | 1.10  (0.82-1.47) |
| Unknown | 0.79  (0.51-1.23) | 0.90  (0.7-1.16) | 0.80  (0.66-0.98) | 0.92  (0.76-1.11) | 0.92  (0.52-1.62) | 0.97  (0.58-1.62) | 1.06  (0.94-1.19) | 0.96  (0.86-1.06) | 1.21  (0.95-1.53) | 1.05  (0.85-1.3) | 1.20  (0.90-1.60) | 1.04  (0.81-1.32) | 0.99  (0.53-1.84) | 1.34  (0.72-2.5) |
| **Season** |  |  |  |  |  |  |  |  |  |  |  |  |  |  |
| Spring | 0.95  (0.83-1.09) | 0.95  (0.87-1.04) | 0.92  (0.86-0.98) | 0.97  (0.91-1.03) | 0.85  (0.70-1.03) | 1.03  (0.85-1.25) | 0.93  (0.89-0.97) | 0.95  (0.91-0.98) | 0.88  (0.80-0.97) | 0.88  (0.81-0.95) | 1.14  (1.01-1.29) | 0.90  (0.83-0.99) | 1.10  (0.87-1.38) | 0.90  (0.68-1.19) |
| Summer | 1.07  (0.93-1.24) | 1.06  (0.96-1.16) | 0.90  (0.84-0.96) | 0.97  (0.9-1.04) | 0.87  (0.71-1.06) | 0.95  (0.77-1.19) | 0.97  (0.93-1.01) | 1.02  (0.98-1.06) | 0.97  (0.88-1.07) | 0.94  (0.86-1.03) | 1.30  (1.15-1.47) | 1.00  (0.92-1.10) | 1.26  (1.00-1.6) | 1.09  (0.83-1.44) |
| Winter | 0.9  (0.80-1.01) | 1.00  (0.92-1.08) | 1.01  (0.95-1.07) | 1.02  (0.96-1.09) | 1.00  (0.84-1.20) | 1.06  (0.88-1.28) | 1.06  (1.01-1.10) | 0.99  (0.96-1.03) | 1.02  (0.94-1.12) | 0.96  (0.89-1.03) | 1.05  (0.93-1.18) | 0.96  (0.88-1.04) | 1.16  (0.92-1.46) | 1.21  (0.93-1.58) |
| **Region** |  |  |  |  |  |  |  |  |  |  |  |  |  |  |
| London | 0.79  (0.62-1.02) | 0.76  (0.65-0.89) | 1.02  (0.88-1.19) | 0.97  (0.85-1.11) | 0.98  (0.64-1.49) | 0.60  (0.34-1.04) | 0.95  (0.86-1.05) | 0.98  (0.90-1.06) | 0.9  (0.74-1.09) | 0.73  (0.59-0.92) | 1.08  (0.88-1.33) | 0.99  (0.85-1.17) | 1.29  (0.90-1.85) | 1.20  (0.74-1.95) |
| North East | 1.09  (0.88-1.36) | 0.86  (0.73-1.02) | 0.99  (0.90-1.09) | 0.99  (0.89-1.10) | 1.04  (0.78-1.38) | 1.20  (0.89-1.62) | 1.03  (0.97-1.10) | 0.97  (0.91-1.03) | 1.02  (0.88-1.17) | 0.91  (0.79-1.04) | 1.19  (0.98-1.44) | 1.06  (0.91-1.23) | 1.06  (0.73-1.53) | 1.20  (0.77-1.89) |
| North West | 0.87  (0.74-1.03) | 0.97  (0.86-1.09) | 0.78  (0.72-0.85) | 0.99  (0.92-1.08) | 0.81  (0.64-1.03) | 0.96  (0.75-1.22) | 0.75  (0.71-0.80) | 1.01  (0.96-1.05) | 0.81  (0.72-0.91) | 0.94  (0.85-1.04) | 1.08  (0.93-1.26) | 1.02  (0.91-1.14) | 0.85  (0.62-1.15) | 1.29  (0.94-1.77) |
| West Midlands | 1.22  (0.98-1.52) | 0.97  (0.85-1.12) | 1.17  (1.03-1.31) | 1.11  (0.99-1.23) | 0.90  (0.62-1.29) | 1.14  (0.80-1.63) | 1.20  (1.11-1.29) | 1.08  (1.02-1.15) | 1.07  (0.90-1.26) | 1.10  (0.95-1.27) | 1.36  (1.12-1.65) | 1.19  (1.03-1.37) | 1.09  (0.74-1.61) | 1.20  (0.77-1.88) |
| Yorkshire and The Humber | 0.98  (0.84-1.14) | 0.98  (0.90-1.08) | 0.91  (0.85-0.98) | 1.02  (0.95-1.09) | 0.90  (0.73-1.11) | 1.10  (0.88-1.37) | 0.96  (0.91-1.01) | 1.00  (0.97-1.04) | 0.97  (0.87-1.07) | 0.95  (0.87-1.04) | 1.23  (1.07-1.40) | 1.05  (0.95-1.16) | 0.98  (0.76-1.26) | 1.00  (0.74-1.36) |
| South East | 0.97  (0.80-1.18) | 1.08  (0.94-1.25) | 0.76  (0.69-0.85) | 0.93  (0.84-1.03) | 0.95  (0.72-1.26) | 1.25  (0.95-1.64) | 0.92  (0.86-0.99) | 0.97  (0.92-1.03) | 0.87  (0.76-1.01) | 1.03  (0.91-1.17) | 1.15  (0.96-1.38) | 1.30  (1.14-1.48) | 1.02  (0.73-1.42) | 1.49  (1.04-2.14) |
| East Midlands | 1.08  (0.94-1.24) | 1.10  (1.00-1.20) | 1.06  (0.99-1.13) | 1.10  (1.02-1.17) | 1.01  (0.82-1.23) | 1.21  (0.98-1.49) | 1.09  (1.04-1.14) | 1.09  (1.05-1.13) | 1.07  (0.97-1.18) | 1.11  (1.02-1.21) | 1.14  (1.00-1.3) | 1.12  (1.02-1.23) | 1.01  (0.80-1.29) | 0.94  (0.70-1.25) |
| South West | 0.79  (0.67-0.95) | 1.02  (0.90-1.16) | 0.73  (0.67-0.79) | 0.95  (0.88-1.04) | 0.77  (0.59-1.00) | 1.22  (0.95-1.56) | 0.83  (0.79-0.88) | 1.05  (1.0-1.09) | 0.86  (0.76-0.97) | 1.07  (0.96-1.2) | 1.4  (1.21-1.61) | 1.37  (1.23-1.52) | 1.15  (0.87-1.50) | 1.49  (1.09-2.04) |
| **Flu vaccination** |  |  |  |  |  |  |  |  |  |  |  |  |  |  |
| Yes | 0.98  (0.87-1.09) | 1.00  (0.93-1.08) | 0.81  (0.77-0.85) | 1.05  (0.99-1.11) | 1.09  (0.92-1.28) | 1.03  (0.87-1.21) | 0.81  (0.78-0.84) | 0.97  (0.94-1.00) | 0.95  (0.88-1.02) | 0.99  (0.92-1.06) | 0.92  (0.82-1.04) | 1.04  (0.95-1.13) | 0.74  (0.57-0.96) | 0.97  (0.74-1.28) |
| **Count of antibiotic prescription in the one year before** | 1.20  (1.17-1.22) | 1.16  (1.14-1.18) | 1.15  (1.14-1.16) | 1.11  (1.10-1.12) | 1.17  (1.14-1.20) | 1.13  (1.10-1.16) | 1.12  (1.11-1.12) | 1.10  (1.10-1.11) | 1.11  (1.10-1.12) | 1.09  (1.08-1.11) | 1.18  (1.15-1.21) | 1.16  (1.14-1.19) | 1.06  (1.01-1.12) | 1.16  (1.09-1.24) |
| ^1^ HR, hazard ratio.  ^2^ CI, confidence interval.  ^3^ ABs, antibiotics prescribed or not.  ^4^ BMI, Body Mass Index recorded in the last 5 years.  ^5^ CCI, Charlson Comorbidities Index, measured from 17 weighted conditions, including myocardial infarction, congestive heart failure, peripheral vascular disease, cerebrovascular disease, dementia, chronic pulmonary disease, Connective tissue disease, ulcer disease, mild liver disease, diabetes, hemiplegia, moderate or severe renal disease, diabetes with complications, any malignancy (including leukaemia and lymphoma), moderate or severe liver disease, metastatic solid tumour, and AIDS.  ^6^ IMD, Multiple Deprivation Index, quintile measured from patient-level address.  Reference group for variable sex is female, for age is 18-25, for BMI is healthy weight, for ethnicity is non-white, for CCI is very low, for smoking status is ex-smoker, for IMD is 2, for season is autumn, for region is east, for flu vaccination is no. | | | | | | | | | | | | | | |

## Hazard ratios of antibiotics

**S13 Table. Crude hazard ratios of prescribed antibiotics as a predictor variable in Cox models for hospital admissions related to incident and prevalent other common infections, including sinusitis, otitis media, and otitis externa.**

|  | **Sinusitis, crude HR^1^**  **(95% CI^2^)** | | **Otitis media, crude HR (95% CI)** | | **Otitis externa, crude HR (95% CI)** | |
| --- | --- | --- | --- | --- | --- | --- |
|  | **Incident** | **Prevalent** | **Incident** | **Prevalent** | **Incident** | **Prevalent** |
| **Antibiotic exposure** |  |  |  |  |  |  |
| No exposure | reference | reference | reference | reference | reference | reference |
| Exposed | 0.68  (0.60-0.77) | 0.69  (0.49-0.97) | 0.65  (0.58-0.72) | 0.68  (0.52-0.89) | 1.73  (1.61-1.86) | 1.00  (0.87-1.13) |
| **Antibiotic type^3^** |  |  |  |  |  |  |
| Most prescribed | reference | reference | reference | reference | reference | reference |
| Second most prescribed | 1.08  (0.91-1.28) | 1.12  (0.60-2.10) | 1.38  (3.20-5.09) | 1.58  (2.52-14.83) | 1.53  (1.32-1.78) | 1.22  (0.89-1.68) |
| Others | 1.73  (1.47-2.03) | 1.73  (0.97-3.09) | 1.85  (5.18-7.92) | 2.31  (4.48-35.28) | 0.82  (0.72-0.92) | 1.28  (0.98-1.68) |
| None | 1.45  (1.23-1.70) | 1.35  (0.74-2.44) | 1.85  (5.06-8.33) | 2.02  (3.62-24.04) | 1.69  (1.46-1.95) | 1.46  (1.09-1.97) |
| **Stratified by sex category** |  |  |  |  |  |  |
| Male | 0.66  (0.53-0.81) | 0.74  (0.41-1.36) | 0.61  (0.52-0.72) | - | 1.60  (1.43-1.80) | 0.96  (0.80-1.15) |
| Female | 0.67  (0.58-0.78) | 0.83  (0.55-1.25) | 0.70  (0.61-0.80) | 0.58  (0.41-0.82) | 1.77  (1.61-1.95) | 0.96  (0.79-1.16) |
| **Stratified by age category** |  |  |  |  |  |  |
| 18-24 | 0.69  (0.43-1.10) | 1.99  (0.04-107.40) | 1.01  (0.66-1.54) | - | 1.68  (1.28-2.21) | 1.06  (0.60-1.89) |
| 25-34 | 0.65  (0.47-0.89) | 0.35  (0.13-0.93) | 0.59  (0.46-0.77) | 0.94  (0.47-1.85) | 1.77  (1.46-2.14) | 1.08  (0.69-1.68) |
| 35-44 | 0.54  (0.40-0.73) | 0.79  (0.35-1.78) | - | - | 1.75  (1.40-2.18) | 0.67  (0.45-1.00) |
| 45-54 | 0.60  (0.44-0.82) | 0.62  (0.29-1.33) | 0.73  (0.54-0.97) | 0.42  (0.18-0.96) | 1.53  (1.25-1.85) | 0.68  (0.44-1.04) |
| 55-64 | 0.55  (0.41-0.73) | - | 0.55  (0.41-0.73) | - | 1.84  (1.50-2.25) | 1.23  (0.85-1.79) |
| 65-74 | 0.84  (0.61-1.16) | - | 0.68  (0.52-0.89) | - | 1.65  (1.37-1.98) | 1.23  (0.85-1.79) |
| 75+ | 0.68  (0.51-0.91) | 0.64  (0.27-1.49) | 0.74  (0.59-0.93) | 0.47  (0.26-0.85) | 1.62  (1.40-1.87) | 0.97  (0.78-1.19) |
| **Stratified by time** |  |  |  |  |  |  |
| Pre-pandemic | 0.68  (0.57-0.82) | - | 0.60  (0.51-0.70) | 0.73  (0.50-1.06) | 1.85  (1.65-2.06) | 0.89  (0.73-1.08) |
| Beginning and during pandemic | 0.46  (0.35-0.62) | - | 0.60  (0.47-0.76) | - | 1.46  (1.25-1.72) | 1.14  (0.85-1.52) |
| After 2^nd^ lockdown | 0.66  (0.57-0.76) | - | 0.60  (0.53-0.68) | 0.72  (0.53-0.99) | 1.69  (1.54-1.84) | 1.04  (0.89-1.21) |
| ^1^ HR, hazard ratio.  ^2^ CI, confidence interval.  ^3^ The most prescribed and the second most prescribed type of antibiotic are respectively amoxicillin and doxycycline for sinusitis, amoxicillin and clarithromycin for otitis media, and amoxicillin and flucloxacillin for otitis externa. | | | | | | |

**S14 Table. Crude hazard ratios of prescribed antibiotics as a predictor variable in Cox models for hospital admissions related to incident and prevalent upper respiratory tract infections (URTI), including specific URTI, cough, cold with cough, and otitis externa.**

|  | **Specific URTI,**  **crude HR^1^**  **(95% CI^2^)** | | **Cough, crude HR**  **(95% CI)** | | **Cold with cough, crude HR (95% CI)** | | **Sore throat, crude HR (95% CI)** | |
| --- | --- | --- | --- | --- | --- | --- | --- | --- |
|  | **Incident** | **Prevalent** | **Incident** | **Prevalent** | **Incident** | **Prevalent** | **Incident** | **Prevalent** |
| **Antibiotic exposure** |  |  |  |  |  |  |  |  |
| No exposure | reference | reference | reference | reference | reference | reference | reference | reference |
| Exposed | 0.79  (0.75-0.84) | 0.68  (0.55-0.84) | 1.35  (1.31-1.39) | 1.18  (1.07-1.30) | 0.90  (0.89-0.92) | 0.81  (0.78-0.85) | 0.79  (0.75-0.83) | 0.63  (0.56-0.72) |
| **Antibiotic type^3^** |  |  |  |  |  |  |  |  |
| Most prescribed | reference | reference | reference | reference | reference | reference | reference | reference |
| Second most prescribed | 0.98  (0.91-1.06) | 0.70  (0.51-0.96) | 1.00  (0.95-1.06) | 0.93  (0.79-1.11) | 0.98  (0.95-1.01) | 0.94  (0.87-1.01) | 0.86  (0.79-0.94) | 0.75  (0.61-0.93) |
| Others | 1.37  (1.29-1.46) | 1.39  (1.06-1.81) | 0.80  (0.77-0.84) | 0.91  (0.80-1.05) | 1.20  (1.17-1.22) | 1.34  (1.26-1.43) | 1.15  (1.05-1.26) | 1.39  (1.16-1.68) |
| None | 1.55  (1.44-1.66) | 1.18  (0.89-1.58) | 1.47  (1.39-1.55) | 1.36  (1.15-1.60) | 1.57  (1.52-1.61) | 1.38  (1.29-1.48) | 1.03  (0.93-1.15) | 1.09  (0.85-1.39) |
| **Stratified by sex category** |  |  |  |  |  |  |  |  |
| Male | 0.76  (0.70-0.83) | 0.66  (0.47-0.93) | 1.39  (1.32-1.46) | 1.27  (1.10-1.47) | 0.93  (0.91-0.96) | 0.81  (0.76-0.86) | 0.82  (0.75-0.89) | 0.69  (0.56-0.85) |
| Female | 0.84  (0.78-0.90) | 0.59  (0.45-0.76) | 1.34  (1.28-1.40) | 1.18  (1.04-1.35) | 0.87  (0.84-0.89) | 0.82  (0.77-0.87) | 0.82  (0.76-0.88) | 0.64  (0.55-0.74) |
| **Stratified by age category** |  |  |  |  |  |  |  |  |
| 18-24 | 0.90  (0.69-1.18) | 0.55  (0.20-1.47) | 0.98  (0.77-1.26) | 0.83  (0.33-2.08) | 0.74  (0.63-0.86) | 0.72  (0.47-1.09) | 0.68  (0.61-0.75) | 0.60  (0.49-0.73) |
| 25-34 | 0.85  (0.69-1.05) | 0.55  (0.20-1.47) | 1.06  (0.90-1.25) | 1.19  (0.73-1.95) | 0.73  (0.66-0.81) | 0.82  (0.64-1.05) | 0.74  (0.67-0.83) | 0.45  (0.35-0.56) |
| 35-44 | 0.70  (0.57-0.86) | 1.74  (0.59-5.10) | 1.03  (0.89-1.19) | 0.83  (0.51-1.35) | 0.71  (0.64-0.78) | 0.82  (0.65-1.04) | 0.81  (0.70-0.93) | 0.64  (0.43-0.94) |
| 45-54 | 0.81  (0.68-0.97) | 0.55  (0.27-1.12) | 1.25  (1.12-1.39) | 1.26  (0.94-1.68) | 0.79  (0.74-0.85) | 0.77  (0.66-0.90) | 0.93  (0.78-1.09) | 0.76  (0.47-1.21) |
| 55-64 | 0.86  (0.73-1.02) | 0.53  (0.26-1.06) | 1.25  (1.15-1.37) | - | 0.88  (0.83-0.93) | 0.87  (0.77-0.98) | 0.90  (0.74-1.08) | 0.83  (0.46-1.50) |
| 65-74 | 0.68  (0.59-0.77) | 0.51  (0.33-0.78) | 1.40  (1.31-1.49) | 1.40  (1.15-1.70) | 0.92  (0.88-0.96) | 0.78  (0.71-0.85) | 0.89  (0.73-1.08) | 1.90  (0.99-3.63) |
| 75+ | 0.86  (0.79-0.95) | 0.68  (0.50-0.93) | 1.41  (1.34-1.48) | 1.21  (1.04-1.41) | 0.90  (0.88-0.93) | 0.82  (0.77-0.87) | 1.09  (0.92-1.30) | 0.81  (0.40-1.63) |
| **Stratified by time** |  |  |  |  |  |  |  |  |
| Pre-pandemic | 0.91  (0.84-0.98) | 0.75  (0.57-0.99) | 1.41  (1.34-1.48) | 1.05  (0.91-1.22) | 0.88  (0.86-0.91) | 0.81  (0.76-0.86) | 0.93  (0.86-1.01) | 0.70  (0.59-0.85) |
| Beginning and during pandemic | 0.74  (0.62-0.88) | 0.64  (0.33-1.25) | 1.53  (1.41-1.65) | 1.49  (1.19-1.87) | 1.05  (1.00-1.10) | 0.96  (0.86-1.08) | 0.81  (0.71-0.92) | 0.64  (0.47-0.88) |
| After 2^nd^ lockdown | 0.89  (0.83-0.95) | 0.74  (0.58-0.93) | 1.46  (1.40-1.52) | 1.18  (1.05-1.32) | 0.94  (0.92-0.96) | 0.84  (0.80-0.88) | 0.90  (0.84-0.96) | 0.66  (0.57-0.76) |
| ^1^ HR, hazard ratio.  ^2^ CI, confidence interval.  ^3^ The most prescribed and the second most prescribed type of antibiotic are respectively amoxicillin and doxycycline for URTI, cough, and cold with cough, and phenoxymethylpenicillin and clarithromycin for sore throat. | | | | | | | | |
