# Supplementary material for: Evaluation of the impact of COVID-19 pandemic on hospital admission related to common infections: Risk prediction models to tackle antimicrobial resistance in primary care
Source: PLoS One. 2024 Dec 31;19(12):e0311515. doi: 10.1371/journal.pone.0311515 (PMC11687718; doi:10.1371/journal.pone.0311515)
Supplement: S4 Appendix — (DOCX) [file pone.0311515.s004.docx]

# Cox models with pre-pandemic data

Due to the fluctuations of counts of hospital admissions particularly related to UTI (shown in Table 2), we split data into four periods regarding COVID-19 status (introduced in the Methods section). S15 Table shows C-statistics of all converged Cox models without extreme HRs using development and validation splits of pre-pandemic data for hospital admission related to common infections. The models with pre-pandemic data showed strong association in age category 75+ and CCI category very high with infection-related complication. S16-S19 Tables present HRs of Cox models for infection-related hospital admissions using pre-pandemic data. The calibration plots of these Cox models are displayed in S1-S10 Figs. These figures indicate that models for LRTI, URTI, and UTI were well-calibrated, but those for other common infections were less well calibrated.

## Performance

**S15 Table. C-statistics of Cox models for hospital admissions related to common infections, including lower respiratory tract infection, upper respiratory tract infection, urinary tract infection (UTI), sinusitis, otitis media, and otitis externa, using pre-pandemic data (from 1 January 2019 to 31 December 2019).**

|  | | | **C-statistics** | |
| --- | --- | --- | --- | --- |
|  |  |  | **Development dataset** | **Validation dataset** |
| LRTI | Incident | No ABs^1^ | 0.68 | 0.67 |
|  |  | With ABs | 0.72 | 0.72 |
|  | Prevalent | No ABs | 0.67 | 0.67 |
|  |  | With ABs | 0.68 | 0.67 |
| URTI | Incident | No ABs | 0.73 | 0.72 |
|  |  | With ABs | 0.71 | 0.71 |
|  | Prevalent | No ABs | 0.71 | 0.72 |
|  |  | With ABs | 0.69 | 0.68 |
| UTI | Incident | No ABs | 0.73 | 0.73 |
|  |  | With ABs | 0.75 | 0.75 |
|  | Prevalent | No ABs | 0.70 | 0.71 |
|  |  | With ABs | 0.69 | 0.70 |
| Sinusitis | Incident | No ABs | 0.73 | 0.64 |
|  |  | With ABs | - | - |
|  | Prevalent | No ABs | - | - |
|  |  | With ABs | - | - |
| Otitis media | Incident | No ABs | 0.71 | 0.64 |
|  |  | With ABs | 0.67 | 0.68 |
|  | Prevalent | No ABs | - | - |
|  |  | With ABs | - | - |
| Otitis externa | Incident | No ABs | 0.71 | 0.70 |
|  |  | With ABs | - | - |
|  | Prevalent | No ABs | 0.76 | 0.72 |
|  |  | With ABs | - | - |
| URTI components | |  |  |  |
| Specific URTI | Incident | No ABs | 0.75 | 0.76 |
|  |  | With ABs | 0.72 | 0.73 |
|  | Prevalent | No ABs | - | - |
|  |  | With ABs | - | - |
| Cough | Incident | No ABs | 0.73 | 0.73 |
|  |  | With ABs | 0.71 | 0.71 |
|  | Prevalent | No ABs | 0.75 | 0.70 |
|  |  | With ABs | 0.70 | 0.68 |
| Cold with cough | Incident | No ABs | 0.74 | 0.74 |
|  |  | With ABs | 0.71 | 0.71 |
|  | Prevalent | No ABs | 0.72 | 0.71 |
|  |  | With ABs | 0.68 | 0.69 |
| Sore throat | Incident | No ABs | 0.64 | 0.63 |
|  |  | With ABs | 0.62 | 0.59 |
|  | Prevalent | No ABs | 0.67 | 0.61 |
|  |  | With ABs | 0.68 | 0.67 |
| ^1^ABs, antibiotics prescribed or not. | | | | |

## Hazard ratios

**S16 Table. Adjusted hazard ratios of Cox models for hospital admissions related to incident common infections, including lower respiratory tract infection (LRTI), upper respiratory tract infection (URTI), and urinary tract infection (UTI), using pre-pandemic data (from 1 January 2019 to 31 December 2019).**

|  | **LRTI, adjusted HR^1^ (95% CI^2^)** | **URTI, adjusted HR**  **(95% CI)** | **UTI, adjusted HR (95% CI)** |
| --- | --- | --- | --- |
| **Sex** |  |  |  |
| Male | 1.17 (1.10-1.23) | 1.14 (1.10-1.19) | 1.60 (1.51-1.71) |
| **Age** |  |  |  |
| 25-34 | 1.13 (0.86-1.48) | 0.98 (0.87-1.09) | 0.82 (0.65-1.04) |
| 35-44 | 1.31 (1.01-1.70) | 0.89 (0.79-1.00) | 0.99 (0.78-1.25) |
| 45-54 | 1.42 (1.10-1.82) | 0.93 (0.83-1.04) | 1.39 (1.12-1.72) |
| 55-64 | 1.97 (1.55-2.51) | 1.14 (1.02-1.26) | 1.72 (1.40-2.10) |
| 65-74 | 2.97 (2.34-3.77) | 1.66 (1.50-1.84) | 2.93 (2.42-3.54) |
| 75+ | 4.63 (3.67-5.86) | 3.63 (3.28-4.01) | 5.22 (4.35-6.27) |
| **BMI^3^** |  |  |  |
| Underweight | 1.16 (1.01-1.33) | 1.45 (1.32-1.60) | 1.26 (1.06-1.49) |
| Overweight | 0.89 (0.82-0.96) | 0.82 (0.78-0.87) | 0.90 (0.83-0.97) |
| Obese | 0.99 (0.92-1.06) | 0.91 (0.86-0.95) | 1.15 (1.06-1.25) |
| Unknown | 1.14 (1.05-1.24) | 1.27 (1.20-1.35) | 1.16 (1.06-1.27) |
| **Ethnicity** |  |  |  |
| White | 0.96 (0.84-1.09) | 0.91 (0.84-0.99) | 1.21 (1.03-1.41) |
| Unknown | 1.01 (0.88-1.15) | 1.05 (0.97-1.14) | 1.29 (1.09-1.51) |
| **CCI^4^** |  |  |  |
| Low | 1.26 (1.18-1.35) | 1.44 (1.38-1.50) | 1.35 (1.26-1.45) |
| Medium | 1.54 (1.42-1.67) | 2.05 (1.94-2.18) | 1.55 (1.41-1.70) |
| High | 1.65 (1.44-1.89) | 2.72 (2.48-2.98) | 2.00 (1.75-2.29) |
| Very high | 2.14 (1.78-2.58) | 3.47 (3.02-3.98) | 2.48 (2.05-3.00) |
| **Smoking status** |  |  |  |
| Smoker | 1.14 (1.05-1.24) | 0.99 (0.94-1.05) | 1.20 (1.08-1.33) |
| Never smoked | 0.95 (0.90-1.02) | 1.08 (1.03-1.13) | 1.00 (0.94-1.07) |
| Unknown | 1.03 (0.62-1.69) | 0.97 (0.72-1.3) | 0.89 (0.51-1.54) |
| **IMD^5^** |  |  |  |
| 1 (most deprived) | 1.13 (1.04-1.22) | 1.04 (0.99-1.10) | 1.08 (0.99-1.18) |
| 3 | 0.93 (0.86-1.01) | 0.86 (0.82-0.91) | 0.90 (0.82-0.98) |
| 4 | 0.92 (0.85-1.01) | 0.91 (0.86-0.96) | 0.93 (0.85-1.02) |
| 5 (most affluent) | 0.84 (0.77-0.92) | 0.80 (0.76-0.86) | 0.77 (0.70-0.85) |
| Unknown | 1.19 (0.97-1.47) | 0.96 (0.82-1.11) | 0.97 (0.76-1.23) |
| **Season** |  |  |  |
| Spring | 0.91 (0.84-0.98) | 0.99 (0.94-1.05) | 0.90 (0.83-0.98) |
| Summer | 1.03 (0.95-1.11) | 0.94 (0.89-1.00) | 0.96 (0.88-1.04) |
| Winter | 1.00 (0.93-1.08) | 1.09 (1.04-1.14) | 1.02 (0.94-1.10) |
| **Region** |  |  |  |
| London | 0.77 (0.64-0.91) | 0.91 (0.81-1.02) | 1.02 (0.86-1.21) |
| North East | 0.99 (0.88-1.12) | 0.88 (0.81-0.96) | 1.00 (0.88-1.15) |
| North West | 0.83 (0.75-0.92) | 0.78 (0.73-0.83) | 0.84 (0.75-0.94) |
| West Midlands | 0.92 (0.80-1.06) | 1.09 (0.99-1.19) | 1.01 (0.86-1.18) |
| Yorkshire and The Humber | 0.92 (0.84-1.00) | 0.95 (0.90-1.01) | 1.09 (1.00-1.20) |
| South East | 1.00 (0.89-1.13) | 0.91 (0.84-0.98) | 0.90 (0.79-1.02) |
| East Midlands | 1.11 (1.02-1.20) | 1.05 (1.00-1.11) | 1.07 (0.98-1.17) |
| South West | 0.86 (0.78-0.95) | 0.82 (0.76-0.87) | 0.77 (0.69-0.86) |
| **Flu vaccination** |  |  |  |
| Yes | 0.92 (0.87-0.98) | 0.81 (0.78-0.85) | 0.88 (0.83-0.94) |
| **Count of antibiotic prescription in the one year before** | 1.04 (1.03-1.05) | 1.15 (1.14-1.16) | 1.02 (1.01-1.03) |
| ^1^ HR, hazard ratio.  ^2^ CI, confidence interval.  ^3^ BMI, Body Mass Index recorded in the last 5 years.  ^4^ CCI, Charlson Comorbidities Index, measured from 17 weighted conditions, including myocardial infarction, congestive heart failure, peripheral vascular disease, cerebrovascular disease, dementia, chronic pulmonary disease, Connective tissue disease, ulcer disease, mild liver disease, diabetes, hemiplegia, moderate or severe renal disease, diabetes with complications, any malignancy (including leukaemia and lymphoma), moderate or severe liver disease, metastatic solid tumour, and AIDS.  ^5^ IMD, Multiple Deprivation Index, quintile measured from patient-level address.  Reference group for variable sex is female, for age is 18-25, for BMI is healthy weight, for ethnicity is non-white, for CCI is very low, for smoking status is ex-smoker, for IMD is 2, for season is autumn, for region is east, for flu vaccination is no. | | | |

**S17 Table. Adjusted hazard ratios of Cox models for hospital admissions related to common infections, including lower respiratory tract infection (LRTI), upper respiratory tract infection (URTI), urinary tract infection (UTI), sinusitis, otitis media, and otitis externa, using pre-pandemic data (from 1 January 2019 to 31 December 2019).**

|  | **LRTI, adjusted HR (95% CI)** | | | **URTI, adjusted HR (95% CI)** | | | **UTI, adjusted HR (95% CI)** | | |
| --- | --- | --- | --- | --- | --- | --- | --- | --- | --- |
|  | **Incident** | **Prevalent** | | **Incident** | **Prevalent** | | **Incident** | **Prevalent** | |
|  | **With ABs^1^** | **No ABs** | **With ABs** | **No ABs** | **With ABs^1^** | **No ABs** | **With ABs** | **No ABs** | **With ABs^1^** |
| **Sex** |  |  |  |  |  |  |  |  |  |
| Male | 1.12  (1.09-1.16) | 1.17  (1.06-1.3) | 1.21  (1.11-1.31) | 1.13  (1.1-1.17) | 1.14  (1.06-1.24) | 1.23  (1.15-1.32) | 2.15  (2.06-2.23) | 1.74  (1.56-1.94) | 1.95  (1.79-2.11) |
| **Age** |  |  |  |  |  |  |  |  |  |
| 25-34 | 1.06  (0.9-1.24) | 1.52  (0.81-2.86) | 0.81  (0.47-1.38) | 0.86  (0.79-0.93) | 0.63  (0.5-0.79) | 0.58  (0.45-0.73) | 1.04  (0.9-1.19) | 1.03  (0.68-1.56) | 1.16  (0.83-1.62) |
| 35-44 | 1.03  (0.88-1.2) | 1.23  (0.66-2.29) | 0.87  (0.53-1.43) | 0.75  (0.69-0.82) | 0.46  (0.36-0.58) | 0.4  (0.32-0.52) | 1.01  (0.88-1.17) | 0.89  (0.57-1.38) | 1.23  (0.88-1.71) |
| 45-54 | 1.12  (0.96-1.3) | 1.26  (0.69-2.31) | 1.14  (0.71-1.84) | 0.81  (0.75-0.88) | 0.53  (0.43-0.66) | 0.59  (0.48-0.73) | 1.16  (1.02-1.32) | 1.0  (0.67-1.5) | 1.13  (0.82-1.55) |
| 55-64 | 1.41  (1.22-1.63) | 1.57  (0.87-2.84) | 1.35  (0.85-2.16) | 0.99  (0.91-1.06) | 0.5  (0.41-0.62) | 0.64  (0.53-0.79) | 1.48  (1.31-1.68) | 1.12  (0.76-1.64) | 1.27  (0.94-1.72) |
| 65-74 | 1.91  (1.66-2.21) | 2.61  (1.46-4.68) | 1.77  (1.12-2.82) | 1.31  (1.22-1.42) | 0.77  (0.63-0.94) | 0.88  (0.73-1.07) | 2.13  (1.89-2.41) | 1.82  (1.27-2.61) | 1.69  (1.26-2.27) |
| 75+ | 3.96  (3.43-4.57) | 3.58  (2.01-6.39) | 2.83  (1.79-4.49) | 2.75  (2.56-2.96) | 1.32  (1.09-1.6) | 1.44  (1.19-1.73) | 4.41  (3.92-4.96) | 2.77  (1.95-3.94) | 3.04  (2.28-4.06) |
| **BMI^2^** |  |  |  |  |  |  |  |  |  |
| Underweight | 1.46  (1.34-1.6) | 1.24  (0.96-1.6) | 1.38  (1.12-1.69) | 1.48  (1.37-1.6) | 1.5  (1.22-1.85) | 1.37  (1.13-1.66) | 1.41  (1.26-1.58) | 1.21  (0.86-1.7) | 1.2  (0.93-1.55) |
| Overweight | 0.8  (0.77-0.84) | 0.93  (0.81-1.06) | 0.73  (0.66-0.82) | 0.82  (0.79-0.86) | 0.87  (0.78-0.97) | 0.8  (0.73-0.88) | 0.91  (0.87-0.96) | 0.99  (0.86-1.14) | 0.95  (0.85-1.05) |
| Obese | 0.85  (0.81-0.89) | 0.83  (0.72-0.95) | 0.83  (0.75-0.92) | 0.89  (0.86-0.93) | 0.91  (0.82-1.02) | 0.85  (0.78-0.94) | 1.03  (0.98-1.08) | 1.19  (1.04-1.37) | 0.97  (0.87-1.07) |
| Unknown | 1.03  (0.98-1.09) | 1.1  (0.93-1.29) | 1.01  (0.89-1.15) | 1.06  (1.02-1.11) | 1.31  (1.16-1.47) | 1.09  (0.97-1.22) | 1.08  (1.02-1.14) | 1.23  (1.04-1.45) | 1.08  (0.96-1.22) |
| **Ethnicity** |  |  |  |  |  |  |  |  |  |
| White | 1.02  (0.94-1.1) | 0.94  (0.73-1.22) | 1.06  (0.87-1.3) | 1.07  (1.01-1.14) | 1.06  (0.88-1.27) | 1.06  (0.89-1.25) | 1.02  (0.93-1.12) | 0.85  (0.65-1.1) | 0.87  (0.72-1.06) |
| Unknown | 1.13  (1.05-1.23) | 1.12  (0.86-1.46) | 1.24  (1.01-1.53) | 1.19  (1.12-1.27) | 1.22  (1.01-1.46) | 1.16  (0.98-1.38) | 1.14  (1.04-1.25) | 1.0  (0.77-1.31) | 0.96  (0.78-1.17) |
| **CCI^3^** |  |  |  |  |  |  |  |  |  |
| Low | 1.32  (1.27-1.37) | 1.18  (1.04-1.34) | 1.24  (1.13-1.37) | 1.3  (1.26-1.35) | 1.48  (1.35-1.63) | 1.31  (1.21-1.42) | 1.49  (1.43-1.56) | 1.41  (1.24-1.61) | 1.47  (1.34-1.61) |
| Medium | 1.73  (1.64-1.82) | 1.63  (1.39-1.9) | 1.67  (1.48-1.88) | 1.76  (1.68-1.84) | 2.0  (1.77-2.26) | 1.7  (1.53-1.89) | 1.81  (1.71-1.92) | 2.03  (1.75-2.37) | 1.74  (1.54-1.96) |
| High | 2.27  (2.09-2.47) | 1.78  (1.4-2.28) | 1.82  (1.49-2.21) | 2.1  (1.95-2.27) | 2.14  (1.75-2.6) | 2.14  (1.81-2.54) | 2.22  (2.03-2.43) | 1.9  (1.51-2.4) | 2.1  (1.76-2.52) |
| Very high | 2.66  (2.33-3.04) | 2.21  (1.59-3.07) | 2.74  (2.09-3.59) | 2.85  (2.55-3.19) | 2.44  (1.8-3.31) | 3.09  (2.42-3.93) | 2.77  (2.4-3.19) | 2.19  (1.55-3.12) | 2.62  (1.97-3.47) |
| **Smoking** |  |  |  |  |  |  |  |  |  |
| Smoker | 0.89  (0.84-0.94) | 0.98  (0.83-1.17) | 0.9  (0.79-1.03) | 0.93  (0.9-0.97) | 0.92  (0.82-1.05) | 0.85  (0.77-0.95) | 1.2  (1.12-1.27) | 1.09  (0.9-1.32) | 1.25  (1.09-1.43) |
| Never smoked | 0.98  (0.95-1.02) | 0.9  (0.8-1.01) | 1.0  (0.91-1.09) | 0.98  (0.95-1.02) | 0.92  (0.84-1.0) | 0.96  (0.88-1.04) | 0.99  (0.95-1.03) | 0.92  (0.82-1.03) | 0.99  (0.91-1.07) |
| Unknown | 0.93  (0.65-1.35) | 0.32  (0.04-2.28) | 1.1  (0.41-2.96) | 1.01  (0.83-1.23) | 1.16  (0.69-1.96) | 1.26  (0.76-2.09) | 0.92  (0.63-1.35) | 0.74  (0.28-2.0) | 0.64  (0.21-2.0) |
| **IMD^4^** |  |  |  |  |  |  |  |  |  |
| 1 (most deprived) | 1.05  (1.0-1.11) | 1.26  (1.08-1.47) | 1.13  (1.01-1.28) | 1.02  (0.98-1.07) | 1.17  (1.04-1.32) | 1.07  (0.96-1.19) | 1.08  (1.02-1.14) | 1.09  (0.92-1.28) | 1.03  (0.91-1.16) |
| 3 | 0.97  (0.92-1.03) | 0.88  (0.75-1.04) | 1.03  (0.91-1.16) | 0.98  (0.94-1.02) | 0.91  (0.8-1.02) | 1.0  (0.9-1.11) | 0.91  (0.86-0.96) | 0.89  (0.76-1.04) | 0.86  (0.77-0.97) |
| 4 | 0.97  (0.92-1.02) | 0.8  (0.68-0.95) | 0.99  (0.88-1.12) | 0.99  (0.95-1.04) | 0.97  (0.86-1.1) | 0.98  (0.88-1.09) | 0.86  (0.82-0.91) | 0.76  (0.64-0.89) | 0.77  (0.68-0.87) |
| 5 (most affluent) | 0.89  (0.84-0.94) | 0.9  (0.76-1.06) | 0.98  (0.86-1.12) | 0.92  (0.87-0.96) | 0.87  (0.77-1.0) | 0.97  (0.87-1.09) | 0.83  (0.78-0.88) | 0.69  (0.58-0.82) | 0.73  (0.65-0.83) |
| Unknown | 0.84  (0.72-0.97) | 1.23  (0.83-1.82) | 0.97  (0.69-1.35) | 0.95  (0.85-1.06) | 1.21  (0.9-1.61) | 1.07  (0.82-1.39) | 0.96  (0.83-1.11) | 1.02  (0.69-1.52) | 0.93  (0.69-1.26) |
| **Season** |  |  |  |  |  |  |  |  |  |
| Spring | 0.94  (0.9-0.99) | 0.94  (0.81-1.09) | 0.87  (0.78-0.98) | 0.97  (0.94-1.01) | 0.93  (0.83-1.04) | 0.88  (0.8-0.97) | 0.96  (0.91-1.01) | 1.02  (0.89-1.18) | 0.95  (0.86-1.06) |
| Summer | 0.98  (0.93-1.03) | 1.02  (0.87-1.19) | 0.85  (0.76-0.96) | 0.98  (0.94-1.02) | 0.91  (0.81-1.02) | 0.93  (0.83-1.03) | 0.98  (0.93-1.03) | 1.01  (0.87-1.17) | 0.94  (0.84-1.04) |
| Winter | 0.94  (0.9-0.98) | 0.91  (0.79-1.04) | 0.86  (0.78-0.95) | 0.95  (0.92-0.98) | 0.99  (0.9-1.1) | 0.94  (0.86-1.03) | 0.95  (0.9-0.99) | 1.02  (0.88-1.17) | 0.95  (0.85-1.05) |
| **Region** |  |  |  |  |  |  |  |  |  |
| London | 1.01  (0.9-1.12) | 1.06  (0.75-1.49) | 0.95  (0.69-1.32) | 0.89  (0.82-0.97) | 1.17  (0.91-1.49) | 0.78  (0.59-1.03) | 1.07  (0.95-1.19) | 0.96  (0.7-1.32) | 1.1  (0.83-1.45) |
| North East | 0.97  (0.9-1.06) | 0.99  (0.79-1.24) | 0.88  (0.73-1.06) | 0.97  (0.91-1.04) | 0.86  (0.72-1.03) | 0.95  (0.81-1.12) | 1.01  (0.92-1.11) | 0.96  (0.74-1.24) | 1.1  (0.91-1.33) |
| North West | 1.02  (0.96-1.08) | 0.88  (0.73-1.05) | 0.9  (0.78-1.03) | 1.06  (1.01-1.11) | 0.79  (0.69-0.91) | 0.93  (0.82-1.05) | 1.0  (0.94-1.07) | 0.85  (0.7-1.03) | 0.94  (0.82-1.08) |
| West Midlands | 1.11  (1.02-1.21) | 1.04  (0.81-1.34) | 0.94  (0.76-1.16) | 1.13  (1.06-1.21) | 1.14  (0.95-1.38) | 1.21  (1.03-1.44) | 0.96  (0.87-1.05) | 1.09  (0.83-1.43) | 1.02  (0.83-1.26) |
| Yorkshire and The Humber | 0.97  (0.92-1.02) | 0.89  (0.76-1.04) | 0.87  (0.77-0.99) | 1.03  (0.98-1.07) | 0.99  (0.87-1.11) | 0.95  (0.85-1.06) | 1.05  (0.99-1.11) | 0.95  (0.81-1.12) | 1.12  (1.0-1.26) |
| South East | 1.02  (0.95-1.1) | 0.87  (0.68-1.1) | 0.91  (0.76-1.1) | 0.99  (0.93-1.06) | 0.9  (0.76-1.07) | 1.07  (0.92-1.25) | 0.91  (0.84-0.98) | 0.67  (0.51-0.88) | 0.89  (0.74-1.07) |
| East Midlands | 1.08  (1.03-1.14) | 0.97  (0.83-1.14) | 1.07  (0.95-1.2) | 1.12  (1.07-1.17) | 1.08  (0.96-1.21) | 1.11  (1.0-1.23) | 0.95  (0.9-1.0) | 1.13  (0.97-1.32) | 0.99  (0.88-1.11) |
| South West | 1.02  (0.96-1.09) | 0.89  (0.73-1.08) | 0.97  (0.83-1.13) | 1.05  (1.0-1.11) | 0.83  (0.72-0.97) | 1.02  (0.9-1.16) | 0.84  (0.79-0.9) | 0.95  (0.79-1.15) | 0.92  (0.8-1.07) |
| **Flu vaccination** |  |  |  |  |  |  |  |  |  |
| Yes | 0.95  (0.91-0.99) | 0.92  (0.81-1.03) | 0.96  (0.87-1.05) | 0.96  (0.92-0.99) | 0.92  (0.84-1.01) | 0.97  (0.89-1.05) | 0.9  (0.86-0.94) | 0.9  (0.8-1.02) | 0.9  (0.82-0.99) |
| **Count of antibiotic prescription in the one year before** | 1.11  (1.1-1.11) | 1.05  (1.03-1.07) | 1.09  (1.07-1.1) | 1.13  (1.12-1.14) | 1.14  (1.13-1.16) | 1.11  (1.1-1.13) | 1.05  (1.05-1.06) | 1.03  (1.02-1.04) | 1.03  (1.02-1.04) |
| ^1^ HR, hazard ratio.  ^2^ CI, confidence interval.  ^3^ ABs, antibiotics prescribed or not.  ^4^ BMI, Body Mass Index recorded in the last 5 years.  ^5^ CCI, Charlson Comorbidities Index, measured from 17 weighted conditions, including myocardial infarction, congestive heart failure, peripheral vascular disease, cerebrovascular disease, dementia, chronic pulmonary disease, Connective tissue disease, ulcer disease, mild liver disease, diabetes, hemiplegia, moderate or severe renal disease, diabetes with complications, any malignancy (including leukaemia and lymphoma), moderate or severe liver disease, metastatic solid tumour, and AIDS.  ^6^ IMD, Multiple Deprivation Index, quintile measured from patient-level address.  Reference group for variable sex is female, for age is 18-25, for BMI is healthy weight, for ethnicity is non-white, for CCI is very low, for smoking status is ex-smoker, for IMD is 2, for season is autumn, for region is east, for flu vaccination is no. | | | | | | | | | |

**S18 Table. Adjusted hazard ratios of Cox models for hospital admissions related to other common infections, including sinusitis, otitis media, and otitis externa, using pre-pandemic data (from 1 January 2019 to 31 December 2019).**

|  | **Sinusitis, adjusted HR^1^ (95% CI^2^)** | **Otitis media, adjusted HR (95% CI)** | | **Otitis externa, adjusted HR (95% CI)** | |
| --- | --- | --- | --- | --- | --- |
|  | **Incident** | **Incident** | | **Incident** | **Prevalent** |
|  | **No ABs^3^** | **No ABs** | **With ABs** | **No ABs** | **No ABs** |
| **Sex** |  |  |  |  |  |
| Male | 1.41  (1.03-1.95) | 1.41  (1.08-1.83) | 1.17  (0.97-1.42) | 1.18  (1.04-1.35) | 1.36  (1.08-1.71) |
| **Age** |  |  |  |  |  |
| 25-34 | 0.75  (0.38-1.48) | 1.49  (0.80-2.79) | 1.06  (0.75-1.51) | 1.05  (0.75-1.47) | 0.59  (0.32-1.10) |
| 35-44 | 0.72  (0.36-1.42) | 1.26  (0.66-2.40) | 0.86  (0.59-1.24) | 1.04  (0.74-1.45) | 0.68  (0.37-1.24) |
| 45-54 | 0.59  (0.29-1.19) | 1.09  (0.57-2.10) | 0.76  (0.51-1.11) | 1.03  (0.74-1.43) | 0.65  (0.36-1.16) |
| 55-64 | 0.81  (0.41-1.60) | 1.40  (0.74-2.65) | 0.78  (0.53-1.16) | 0.85  (0.61-1.19) | 0.83  (0.47-1.47) |
| 65-74 | 0.41  (0.18-0.91) | 1.21  (0.62-2.38) | 1.02  (0.68-1.54) | 1.03  (0.73-1.45) | 0.78  (0.43-1.42) |
| 75+ | 1.74  (0.83-3.62) | 2.36  (1.23-4.52) | 1.99  (1.33-2.98) | 2.34  (1.69-3.25) | 2.88  (1.66-4.98) |
| **BMI**^2^ |  |  |  |  |  |
| Underweight | 1.51  (0.54-4.21) | 0.95  (0.30-3.08) | 0.88  (0.38-2.0) | 1.19  (0.72-1.99) | 0.47  (0.12-1.94) |
| Overweight | 0.71  (0.46-1.10) | 1.21  (0.83-1.75) | 0.81  (0.62-1.05) | 0.88  (0.73-1.06) | 0.97  (0.71-1.33) |
| Obese | 0.87  (0.57-1.34) | 1.26  (0.87-1.82) | 1.08  (0.84-1.38) | 1.12  (0.94-1.34) | 1.29  (0.95-1.75) |
| Unknown | 1.12  (0.71-1.77) | 1.21  (0.80-1.83) | 0.90  (0.68-1.19) | 0.99  (0.81-1.22) | 0.76  (0.51-1.15) |
| **Ethnicity** |  |  |  |  |  |
| White | 0.76  (0.43-1.34) | 0.87  (0.53-1.42) | 1.12  (0.80-1.58) | 1.00  (0.77-1.29) | 1.23  (0.72-2.12) |
| Unknown | 0.71  (0.39-1.30) | 0.78  (0.47-1.29) | 1.06  (0.74-1.51) | 1.09  (0.84-1.43) | 1.62  (0.94-2.82) |
| **CCI**^3^ |  |  |  |  |  |
| Low | 1.21  (0.84-1.74) | 1.55  (1.15-2.09) | 1.34  (1.09-1.66) | 1.56  (1.34-1.81) | 1.60  (1.23-2.08) |
| Medium | 2.03  (1.12-3.68) | 1.96  (1.23-3.14) | 2.01  (1.43-2.83) | 2.76  (2.24-3.40) | 2.58  (1.84-3.61) |
| High | 2.10  (0.64-6.86) | 2.32  (0.99-5.42) | 1.92  (0.94-3.94) | 3.50  (2.46-4.98) | 2.84  (1.64-4.91) |
| Very high | 2.50  (0.34-18.29) | 1.20  (0.17-8.74) | 4.50  (1.84-11.03) | 4.61  (2.58-8.24) | 1.51  (0.37-6.20) |
| **Smoking** |  |  |  |  |  |
| Smoker | 0.85  (0.53-1.36) | 1.42  (0.99-2.02) | 0.98  (0.76-1.28) | 1.05  (0.86-1.27) | 1.03  (0.73-1.45) |
| Never smoked | 0.72  (0.51-1.02) | 1.04  (0.77-1.39) | 1.12  (0.91-1.36) | 0.87  (0.76-1.01) | 0.86  (0.66-1.11) |
| Unknown | 1.72  (0.39-7.46) | 0.80  (0.11-6.06) | 0.27  (0.04-1.94) | 0.38  (0.09-1.53) | 0.71  (0.09-5.29) |
| **IMD**^4^ |  |  |  |  |  |
| 1 (most deprived) | 1.50  (0.91-2.47) | 0.84  (0.57-1.23) | 1.10  (0.84-1.42) | 1.16  (0.95-1.41) | 1.49  (1.07-2.08) |
| 3 | 1.18  (0.71-1.96) | 0.88  (0.60-1.31) | 1.06  (0.81-1.40) | 1.15  (0.94-1.40) | 0.86  (0.59-1.25) |
| 4 | 1.00  (0.59-1.71) | 0.9  (0.60-1.34) | 0.92  (0.68-1.24) | 1.04  (0.85-1.28) | 0.90  (0.63-1.29) |
| 5 (most affluent) | 1.26  (0.75-2.12) | 0.89  (0.59-1.34) | 1.02  (0.75-1.38) | 1.00  (0.80-1.24) | 0.93  (0.64-1.35) |
| Unknown | 0.80  (0.19-3.41) | 1.47  (0.63-3.44) | 1.13  (0.57-2.24) | 1.34  (0.83-2.16) | 1.03  (0.37-2.86) |
| **Season** |  |  |  |  |  |
| Spring | 1.71  (1.09-2.67) | 0.70  (0.49-0.99) | 0.78  (0.61-0.99) | 0.93  (0.78-1.1) | 0.73  (0.54-0.98) |
| Summer | 1.38  (0.84-2.27) | 0.77  (0.55-1.09) | 0.84  (0.66-1.07) | 0.92  (0.78-1.09) | 0.75  (0.56-0.99) |
| Winter | 1.40  (0.89-2.19) | 0.74  (0.53-1.04) | 0.76  (0.6-0.97) | 0.86  (0.72-1.02) | 0.75  (0.55-1.00) |
| **Region** |  |  |  |  |  |
| London | 1.23  (0.55-2.74) | 0.35  (0.12-0.98) | 1.06  (0.62-1.82) | 1.37  (0.98-1.92) | 2.12  (1.12-4.02) |
| North East | 1.38  (0.69-2.76) | 1.36  (0.80-2.31) | 1.21  (0.77-1.9) | 1.17  (0.86-1.58) | 2.21  (1.39-3.53) |
| North West | 1.01  (0.57-1.79) | 0.88  (0.55-1.4) | 1.40  (1.02-1.94) | 1.14  (0.90-1.45) | 1.47  (0.95-2.29) |
| West Midlands | 0.99  (0.41-2.41) | 1.12  (0.60-2.12) | 1.45  (0.94-2.26) | 1.19  (0.84-1.68) | 1.79  (0.95-3.35) |
| Yorkshire and The Humber | 1.42  (0.88-2.30) | 0.81  (0.53-1.24) | 1.33  (0.99-1.77) | 1.20  (0.97-1.47) | 1.53  (1.03-2.27) |
| South East | 1.25  (0.66-2.39) | 1.03  (0.56-1.87) | 0.99  (0.62-1.61) | 0.97  (0.71-1.31) | 1.32  (0.77-2.26) |
| East Midlands | 0.84  (0.48-1.46) | 1.33  (0.92-1.91) | 1.47  (1.12-1.93) | 1.20  (0.99-1.47) | 1.95  (1.35-2.83) |
| South West | 1.41  (0.82-2.40) | 1.01  (0.62-1.63) | 1.62  (1.17-2.25) | 1.32  (1.06-1.65) | 1.70  (1.12-2.58) |
| **Flu vaccination** |  |  |  |  |  |
| Yes | 1.42  (0.95-2.10) | 1.35  (0.98-1.87) | 1.09  (0.87-1.36) | 1.07  (0.91-1.26) | 0.70  (0.54-0.92) |
| **Count of antibiotic prescription in the one year before** | 1.33  (1.24-1.44) | 1.27  (1.19-1.36) | 1.22  (1.16-1.28) | 1.27  (1.22-1.32) | 1.23  (1.16-1.31) |
| ^1^ HR, hazard ratio.  ^2^ CI, confidence interval.  ^3^ ABs, antibiotics prescribed or not.  ^4^ BMI, Body Mass Index recorded in the last 5 years.  ^5^ CCI, Charlson Comorbidities Index, measured from 17 weighted conditions, including myocardial infarction, congestive heart failure, peripheral vascular disease, cerebrovascular disease, dementia, chronic pulmonary disease, Connective tissue disease, ulcer disease, mild liver disease, diabetes, hemiplegia, moderate or severe renal disease, diabetes with complications, any malignancy (including leukaemia and lymphoma), moderate or severe liver disease, metastatic solid tumour, and AIDS.  ^6^ IMD, Multiple Deprivation Index, quintile measured from patient-level address.  Reference group for variable sex is female, for age is 18-25, for BMI is healthy weight, for ethnicity is non-white, for CCI is very low, for smoking status is ex-smoker, for IMD is 2, for season is autumn, for region is east, for flu vaccination is no. | | | | | |

**S19 Table. Adjusted hazard ratios of Cox models for hospital admissions related to upper respiratory tract infection (URTI), cough, cold with cough, and sore throat, using pre-pandemic data (from 1 January 2019 to 31 December 2019).**

|  | **URTI, adjusted HR^1^ (95% CI^2^)** | | **Cough, adjusted HR (95% CI)** | | | | **Cough, adjusted HR (95% CI)** | | | | **Sore throat, adjusted HR (95% CI)** | | | |
| --- | --- | --- | --- | --- | --- | --- | --- | --- | --- | --- | --- | --- | --- | --- |
|  | **Incident** | | **Incident** | | **Prevalent** | | **Incident** | | **Prevalent** | | **Incident** | | **Prevalent** | |
|  | **No ABs^3^** | **With ABs** | **No ABs** | **With ABs** | **No ABs** | **With ABs** | **No ABs** | **With ABs** | **No ABs** | **With ABs** | **No ABs** | **With ABs** | **No ABs** | **With ABs** |
| **Sex** |  |  |  |  |  |  |  |  |  |  |  |  |  |  |
| Male | 1.30  (1.15-1.48) | 1.14  (1.05-1.24) | 1.12  (1.04-1.20) | 1.12  (1.05-1.20) | 1.13  (0.92-1.39) | 1.08  (0.88-1.34) | 1.12  (1.07-1.18) | 1.13  (1.09-1.17) | 1.17  (1.07-1.29) | 1.17  (1.08-1.26) | 1.50  (1.32-1.71) | 1.31  (1.19-1.44) | 1.33  (1.02-1.72) | 1.42  (1.08-1.88) |
| **Age** |  |  |  |  |  |  |  |  |  |  |  |  |  |  |
| 25-34 | 0.97  (0.7-1.34) | 0.89  (0.66-1.20) | 0.98  (0.73-1.34) | 1.13  (0.84-1.53) | 1.35  (0.53-3.43) | 2.18  (0.64-7.45) | 1.36  (1.09-1.68) | 1.12  (0.95-1.32) | 1.47  (0.87-2.49) | 0.85  (0.53-1.35) | 0.70  (0.59-0.84) | 0.75  (0.67-0.84) | 0.57  (0.42-0.78) | 0.6  (0.44-0.81) |
| 35-44 | 1.07  (0.78-1.49) | 0.95  (0.71-1.27) | 0.95  (0.70-1.28) | 1.06  (0.79-1.41) | 1.04  (0.42-2.61) | 1.10  (0.31-3.85) | 1.33  (1.07-1.64) | 1.05  (0.89-1.23) | 0.99  (0.58-1.69) | 0.69  (0.44-1.08) | 0.60  (0.49-0.74) | 0.63  (0.54-0.72) | 0.43  (0.29-0.65) | 0.29  (0.18-0.46) |
| 45-54 | 0.99  (0.71-1.37) | 1.02  (0.77-1.36) | 1.09  (0.83-1.45) | 1.19  (0.90-1.57) | 1.36  (0.57-3.24) | 1.74  (0.53-5.69) | 1.63  (1.33-2.00) | 1.17  (1.00-1.37) | 1.41  (0.86-2.31) | 1.04  (0.69-1.59) | 0.54  (0.43-0.68) | 0.62  (0.53-0.74) | 0.38  (0.23-0.61) | 0.47  (0.29-0.78) |
| 55-64 | 1.33  (0.97-1.83) | 1.30  (0.99-1.71) | 1.23  (0.94-1.61) | 1.36  (1.03-1.78) | 0.79  (0.33-1.90) | 1.16  (0.36-3.81) | 1.94  (1.59-2.36) | 1.45  (1.25-1.69) | 1.51  (0.93-2.45) | 1.12  (0.74-1.69) | 0.60  (0.47-0.77) | 0.60  (0.49-0.73) | 0.59  (0.35-0.97) | 0.33  (0.16-0.71) |
| 65-74 | 2.40  (1.75-3.29) | 1.63  (1.24-2.14) | 1.65  (1.26-2.15) | 1.81  (1.38-2.36) | 1.35  (0.57-3.17) | 1.97  (0.61-6.36) | 2.96  (2.44-3.60) | 1.99  (1.72-2.31) | 2.41  (1.49-3.90) | 1.44  (0.96-2.16) | 0.72  (0.55-0.95) | 0.66  (0.52-0.84) | 0.28  (0.12-0.67) | 0.66  (0.33-1.31) |
| 75+ | 4.52  (3.33-6.12) | 3.51  (2.69-4.57) | 3.38  (2.60-4.39) | 3.59  (2.76-4.68) | 2.18  (0.94-5.09) | 2.84  (0.89-9.11) | 6.48  (5.35-7.85) | 4.10  (3.55-4.74) | 3.96  (2.45-6.38) | 2.39  (1.60-3.59) | 1.10  (0.83-1.46) | 1.52  (1.23-1.89) | 0.33  (0.12-0.85) | 0.63  (0.27-1.47) |
| **BMI**^4^ |  |  |  |  |  |  |  |  |  |  |  |  |  |  |
| Underweight | 1.47  (1.02-2.11) | 1.79  (1.43-2.23) | 1.41  (1.15-1.72) | 1.41  (1.17-1.7) | 1.83  (1.06-3.17) | 1.59  (0.92-2.75) | 1.41  (1.24-1.59) | 1.44  (1.31-1.58) | 1.63  (1.28-2.06) | 1.37  (1.11-1.68) | 1.06  (0.68-1.66) | 1.17  (0.85-1.59) | 2.33  (1.23-4.44) | 1.15  (0.49-2.68) |
| Overweight | 0.94  (0.79-1.11) | 0.82  (0.73-0.92) | 0.82  (0.74-0.90) | 0.82  (0.75-0.9) | 0.84  (0.63-1.12) | 0.76  (0.57-1.01) | 0.84  (0.78-0.89) | 0.8  (0.77-0.84) | 0.83  (0.73-0.94) | 0.77  (0.7-0.86) | 0.84  (0.69-1.01) | 0.96  (0.84-1.10) | 1.26  (0.85-1.87) | 0.93  (0.62-1.38) |
| Obese | 0.91  (0.77-1.08) | 0.88  (0.79-0.99) | 0.86  (0.78-0.95) | 0.93  (0.85-1.01) | 0.96  (0.73-1.26) | 0.83  (0.63-1.09) | 0.9  (0.84-0.96) | 0.86  (0.82-0.9) | 0.91  (0.80-1.03) | 0.79  (0.71-0.87) | 1.10  (0.92-1.32) | 0.92  (0.81-1.05) | 1.19  (0.80-1.77) | 0.99  (0.68-1.44) |
| Unknown | 1.07  (0.89-1.29) | 1.05  (0.92-1.20) | 1.23  (1.08-1.40) | 1.07  (0.96-1.20) | 1.37  (0.98-1.92) | 1.28  (0.91-1.81) | 1.32  (1.22-1.42) | 1.06  (1.00-1.12) | 1.29  (1.11-1.49) | 0.97  (0.86-1.11) | 1.19  (1.00-1.41) | 0.92  (0.81-1.04) | 1.47  (1.03-2.10) | 1.10  (0.78-1.55) |
| **Ethnicity** |  |  |  |  |  |  |  |  |  |  |  |  |  |  |
| White | 1.02  (0.80-1.30) | 1.14  (0.97-1.35) | 0.89  (0.75-1.06) | 1.02  (0.88-1.18) | 1.04  (0.66-1.65) | 1.19  (0.71-2.00) | 0.98  (0.87-1.09) | 1.03  (0.95-1.11) | 1.04  (0.83-1.31) | 1.12  (0.92-1.38) | 1.09  (0.88-1.35) | 1.03  (0.87-1.22) | 0.9  (0.57-1.41) | 1.09  (0.64-1.86) |
| Unknown | 1.15  (0.89-1.48) | 1.22  (1.03-1.44) | 1.03  (0.86-1.23) | 1.17  (1.00-1.36) | 1.28  (0.80-2.05) | 1.24  (0.73-2.11) | 1.11  (0.99-1.25) | 1.13  (1.05-1.23) | 1.20  (0.95-1.51) | 1.25  (1.02-1.53) | 0.97  (0.78-1.21) | 1.13  (0.95-1.33) | 0.95  (0.60-1.52) | 1.34  (0.78-2.29) |
| **CCI**^5^ |  |  |  |  |  |  |  |  |  |  |  |  |  |  |
| Low | 1.59  (1.38-1.84) | 1.31  (1.19-1.45) | 1.50  (1.37-1.64) | 1.28  (1.19-1.39) | 1.71  (1.33-2.19) | 1.61  (1.25-2.08) | 1.42  (1.34-1.50) | 1.32  (1.26-1.37) | 1.48  (1.32-1.66) | 1.28  (1.16-1.41) | 1.40  (1.20-1.63) | 1.09  (0.97-1.22) | 1.52  (1.13-2.06) | 1.11  (0.80-1.54) |
| Medium | 2.01  (1.63-2.48) | 1.8  (1.57-2.06) | 2.15  (1.92-2.42) | 1.69  (1.52-1.88) | 2.60  (1.90-3.58) | 2.06  (1.48-2.86) | 1.92  (1.78-2.06) | 1.74  (1.65-1.84) | 2.09  (1.82-2.41) | 1.68  (1.5-1.89) | 1.89  (1.4-2.56) | 1.61  (1.27-2.06) | 0.87  (0.27-2.83) | 2.29  (1.14-4.59) |
| High | 3.53  (2.62-4.76) | 2.30  (1.85-2.86) | 3.06  (2.55-3.68) | 1.93  (1.61-2.32) | 2.49  (1.51-4.11) | 2.27  (1.36-3.80) | 2.46  (2.18-2.77) | 2.27  (2.08-2.47) | 2.13  (1.70-2.67) | 1.99  (1.64-2.40) | 1.21  (0.57-2.57) | 1.54  (0.93-2.55) | 3.58  (1.28-10.06) | 1.01  (0.14-7.42) |
| Very high | 3.20  (1.86-5.49) | 3.54  (2.57-4.88) | 4.01  (3.03-5.29) | 2.95  (2.28-3.81) | 1.27  (0.40-4.02) | 4.83  (2.32-10.04) | 3.41  (2.89-4.04) | 2.88  (2.52-3.28) | 3.03  (2.23-4.12) | 3.03  (2.32-3.95) | 3.97  (1.96-8.04) | 3.41  (1.87-6.22) | 4.78  (0.66-34.92) | 5.53  (1.29-23.58) |
| **Smoking** |  |  |  |  |  |  |  |  |  |  |  |  |  |  |
| Smoker | 1.16  (0.96-1.41) | 0.94  (0.82-1.07) | 0.93  (0.83-1.04) | 0.89  (0.81-0.99) | 0.69  (0.48-0.98) | 0.56  (0.39-0.80) | 0.95  (0.89-1.02) | 0.88  (0.84-0.93) | 0.87  (0.75-1.02) | 0.87  (0.77-0.99) | 1.47  (1.24-1.75) | 1.17  (1.04-1.32) | 1.06  (0.76-1.48) | 0.88  (0.62-1.26) |
| Never smoked | 0.88  (0.77-1.01) | 0.93  (0.85-1.03) | 1.17  (1.06-1.28) | 1.01  (0.94-1.09) | 0.95  (0.75-1.19) | 0.98  (0.77-1.24) | 1.11  (1.05-1.17) | 1.00  (0.96-1.04) | 0.94  (0.85-1.05) | 1.00  (0.91-1.09) | 0.95  (0.82-1.10) | 0.89  (0.80-0.98) | 0.79  (0.59-1.05) | 0.82  (0.61-1.09) |
| Unknown | 0.33  (0.08-1.32) | 0.52  (0.19-1.40) | 0.58  (0.19-1.83) | 1.14  (0.56-2.30) | 1.56  (0.21-11.32) | 1.84  (0.25-13.43) | 1.33  (0.86-2.06) | 1.07  (0.75-1.53) | 1.10  (0.41-2.95) | 0.72  (0.23-2.24) | 1.03  (0.67-1.56) | 0.84  (0.63-1.12) | 1.06  (0.55-2.03) | 0.66  (0.32-1.37) |
| **IMD**^6^ |  |  |  |  |  |  |  |  |  |  |  |  |  |  |
| 1 (most deprived) | 1.02  (0.84-1.24) | 1.04  (0.92-1.18) | 0.98  (0.88-1.09) | 1.09  (0.99-1.21) | 1.07  (0.79-1.45) | 0.95  (0.70-1.30) | 1.10  (1.02-1.18) | 1.05  (1.00-1.11) | 1.12  (0.98-1.29) | 1.09  (0.97-1.22) | 0.93  (0.77-1.12) | 1.08  (0.94-1.24) | 1.41  (0.96-2.07) | 1.08  (0.73-1.6) |
| 3 | 0.88  (0.72-1.06) | 0.95  (0.84-1.08) | 0.82  (0.73-0.92) | 0.96  (0.87-1.07) | 0.78  (0.57-1.07) | 0.86  (0.63-1.17) | 0.88  (0.81-0.94) | 0.99  (0.94-1.05) | 0.87  (0.76-1.01) | 1.00  (0.89-1.13) | 0.96  (0.79-1.16) | 1.11  (0.97-1.27) | 1.21  (0.82-1.78) | 1.05  (0.72-1.54) |
| 4 | 1.00  (0.83-1.2) | 0.96  (0.85-1.10) | 0.81  (0.72-0.91) | 1.00  (0.90-1.11) | 0.81  (0.58-1.11) | 0.94  (0.68-1.29) | 0.94  (0.87-1.02) | 1.00  (0.95-1.06) | 0.93  (0.81-1.08) | 0.93  (0.82-1.05) | 0.94  (0.77-1.15) | 1.06  (0.92-1.22) | 1.14  (0.76-1.71) | 0.91  (0.61-1.36) |
| 5 (most affluent) | 0.85  (0.7-1.05) | 0.92  (0.8-1.06) | 0.70  (0.62-0.80) | 0.88  (0.79-0.99) | 0.80  (0.57-1.11) | 0.75  (0.52-1.08) | 0.83  (0.77-0.90) | 0.89  (0.84-0.94) | 0.79  (0.68-0.93) | 1.01  (0.89-1.14) | 1.04  (0.85-1.28) | 1.11  (0.96-1.29) | 1.10  (0.73-1.67) | 1.19  (0.80-1.75) |
| Unknown | 1.13  (0.69-1.86) | 0.70  (0.48-1.03) | 0.77  (0.56-1.07) | 1.04  (0.79-1.35) | 0.89  (0.38-2.04) | 1.06  (0.51-2.21) | 1.15  (0.96-1.39) | 0.92  (0.79-1.06) | 1.04  (0.72-1.51) | 1.13  (0.84-1.51) | 1.39  (0.91-2.13) | 1.18  (0.83-1.67) | 1.16  (0.46-2.94) | 1.20  (0.48-3.03) |
| **Season** |  |  |  |  |  |  |  |  |  |  |  |  |  |  |
| Spring | 1.05  (0.88-1.24) | 0.88  (0.78-1.00) | 1.00  (0.90-1.11) | 1.00  (0.91-1.1) | 0.72  (0.54-0.96) | 0.90  (0.68-1.19) | 0.96  (0.9-1.03) | 0.95  (0.90-1.00) | 0.83  (0.73-0.95) | 0.93  (0.83-1.04) | 1.05  (0.88-1.25) | 0.97  (0.86-1.09) | 1.27  (0.91-1.78) | 0.78  (0.54-1.12) |
| Summer | 0.92  (0.75-1.13) | 0.95  (0.83-1.08) | 0.82  (0.73-0.92) | 0.91  (0.82-1.01) | 0.77  (0.57-1.04) | 0.80  (0.57-1.10) | 0.98  (0.91-1.05) | 0.94  (0.89-0.99) | 0.92  (0.8-1.06) | 0.91  (0.80-1.03) | 1.22  (1.02-1.46) | 0.92  (0.81-1.04) | 1.19  (0.83-1.7) | 1.05  (0.74-1.5) |
| Winter | 0.99  (0.85-1.15) | 1.02  (0.92-1.13) | 1.11  (1.01-1.22) | 0.97  (0.89-1.05) | 0.93  (0.72-1.20) | 0.81  (0.62-1.06) | 1.12  (1.05-1.19) | 0.93  (0.89-0.98) | 0.98  (0.87-1.10) | 0.97  (0.88-1.07) | 1.05  (0.89-1.25) | 0.86  (0.76-0.97) | 1.12  (0.79-1.58) | 1.06  (0.76-1.48) |
| **Region** |  |  |  |  |  |  |  |  |  |  |  |  |  |  |
| London | 0.86  (0.61-1.21) | 0.76  (0.61-0.94) | 0.90  (0.7-1.16) | 0.98  (0.80-1.19) | 1.40  (0.75-2.62) | 0.77  (0.33-1.79) | 0.96  (0.82-1.13) | 1.00  (0.9-1.12) | 1.14  (0.84-1.55) | 0.96  (0.70-1.31) | 1.14  (0.83-1.56) | 1.12  (0.89-1.39) | 1.12  (0.59-2.13) | 0.85  (0.38-1.88) |
| North East | 1.03  (0.75-1.42) | 1.00  (0.81-1.24) | 0.88  (0.75-1.03) | 0.97  (0.83-1.13) | 1.00  (0.64-1.56) | 1.10  (0.70-1.73) | 0.88  (0.79-0.98) | 0.95  (0.88-1.04) | 0.93  (0.76-1.14) | 1.05  (0.88-1.25) | 1.22  (0.92-1.63) | 1.03  (0.82-1.28) | 1.16  (0.67-2.00) | 0.92  (0.48-1.74) |
| North West | 0.84  (0.66-1.07) | 1.09  (0.93-1.27) | 0.74  (0.65-0.85) | 1.08  (0.96-1.22) | 0.82  (0.57-1.18) | 1.17  (0.82-1.65) | 0.78  (0.72-0.85) | 1.00  (0.94-1.07) | 0.82  (0.70-0.97) | 0.95  (0.83-1.09) | 1.07  (0.84-1.34) | 1.29  (1.10-1.51) | 0.96  (0.60-1.53) | 0.84  (0.52-1.36) |
| West Midlands | 1.21  (0.88-1.65) | 1.06  (0.88-1.28) | 0.97  (0.80-1.18) | 1.25  (1.07-1.46) | 1.05  (0.62-1.79) | 1.52  (0.92-2.52) | 1.10  (0.97-1.23) | 1.09  (1.00-1.18) | 1.13  (0.89-1.43) | 1.10  (0.90-1.34) | 1.21  (0.9-1.62) | 1.26  (1.02-1.56) | 1.00  (0.55-1.81) | 1.47  (0.88-2.48) |
| Yorkshire and The Humber | 1.25  (1.02-1.52) | 1.01  (0.89-1.15) | 0.84  (0.74-0.95) | 1.03  (0.92-1.15) | 1.05  (0.76-1.45) | 0.91  (0.64-1.29) | 0.93  (0.87-1.01) | 0.98  (0.92-1.03) | 0.98  (0.85-1.13) | 0.99  (0.87-1.11) | 1.19  (0.97-1.45) | 1.03  (0.89-1.19) | 0.90  (0.60-1.36) | 0.99  (0.66-1.47) |
| South East | 1.20  (0.94-1.54) | 1.05  (0.87-1.28) | 0.74  (0.63-0.88) | 0.86  (0.74-1.01) | 1.11  (0.73-1.67) | 0.88  (0.55-1.42) | 0.90  (0.81-0.99) | 0.93  (0.86-1.01) | 0.83  (0.68-1.03) | 1.00  (0.84-1.19) | 1.34  (1.04-1.74) | 1.39  (1.15-1.67) | 0.85  (0.49-1.47) | 1.46  (0.93-2.31) |
| East Midlands | 1.24  (1.03-1.49) | 1.05  (0.92-1.19) | 0.94  (0.84-1.06) | 1.22  (1.1-1.35) | 1.24  (0.91-1.68) | 1.25  (0.91-1.71) | 1.09  (1.01-1.17) | 1.09  (1.04-1.15) | 1.12  (0.97-1.28) | 1.12  (1.00-1.26) | 1.16  (0.95-1.40) | 1.03  (0.90-1.19) | 1.12  (0.78-1.62) | 0.86  (0.59-1.27) |
| South West | 0.95  (0.75-1.19) | 1.07  (0.91-1.27) | 0.68  (0.59-0.78) | 1.05  (0.93-1.18) | 0.65  (0.42-1.00) | 1.12  (0.78-1.62) | 0.8  (0.73-0.87) | 0.98  (0.92-1.05) | 0.82  (0.68-0.98) | 1.00  (0.86-1.16) | 1.23  (0.99-1.53) | 1.46  (1.25-1.69) | 1.25  (0.83-1.86) | 1.25  (0.82-1.90) |
| **Flu vaccination** |  |  |  |  |  |  |  |  |  |  |  |  |  |  |
| Yes | 0.84  (0.73-0.98) | 1.00  (0.91-1.11) | 0.87  (0.79-0.95) | 0.98  (0.90-1.06) | 1.00  (0.78-1.28) | 1.05  (0.82-1.35) | 0.81  (0.76-0.85) | 0.92  (0.88-0.96) | 0.89  (0.80-0.99) | 0.99  (0.90-1.08) | 0.87  (0.73-1.04) | 1.10  (0.97-1.26) | 0.80  (0.54-1.21) | 1.22  (0.84-1.77) |
| **Count of antibiotic prescription in the one year before** | 1.20  (1.17-1.23) | 1.17  (1.15-1.19) | 1.18  (1.16-1.19) | 1.14  (1.12-1.15) | 1.20  (1.16-1.25) | 1.13  (1.09-1.18) | 1.12  (1.12-1.13) | 1.11  (1.11-1.12) | 1.11  (1.09-1.13) | 1.09  (1.07-1.1) | 1.19  (1.14-1.24) | 1.17  (1.14-1.21) | 1.02  (0.94-1.11) | 1.20  (1.10-1.31) |
| ^1^ HR, hazard ratio.  ^2^ CI, confidence interval.  ^3^ ABs, antibiotics prescribed or not.  ^4^ BMI, Body Mass Index recorded in the last 5 years.  ^5^ CCI, Charlson Comorbidities Index, measured from 17 weighted conditions, including myocardial infarction, congestive heart failure, peripheral vascular disease, cerebrovascular disease, dementia, chronic pulmonary disease, Connective tissue disease, ulcer disease, mild liver disease, diabetes, hemiplegia, moderate or severe renal disease, diabetes with complications, any malignancy (including leukaemia and lymphoma), moderate or severe liver disease, metastatic solid tumour, and AIDS.  ^6^ IMD, Multiple Deprivation Index, quintile measured from patient-level address.  Reference group for variable sex is female, for age is 18-25, for BMI is healthy weight, for ethnicity is non-white, for CCI is very low, for smoking status is ex-smoker, for IMD is 2, for season is autumn, for region is east, for flu vaccination is no. | | | | | | | | | | | | | | |

## Calibration plots

| A  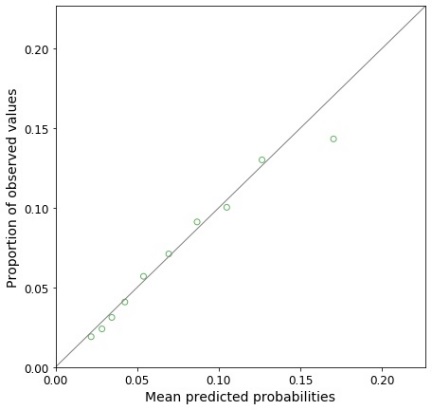 | B  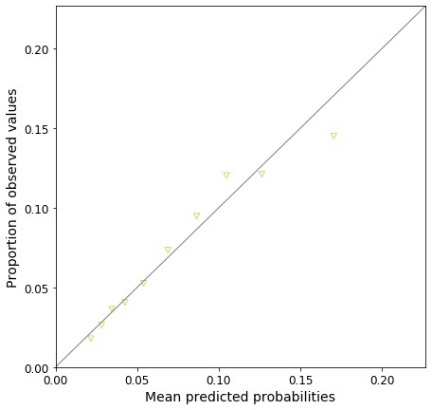 |
| --- | --- |
| C  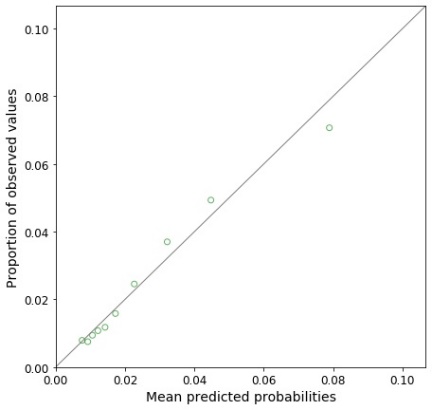 | D  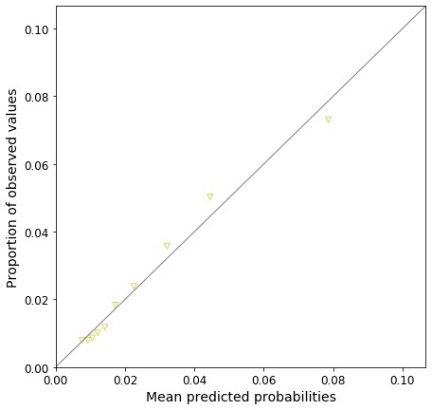 |
| E  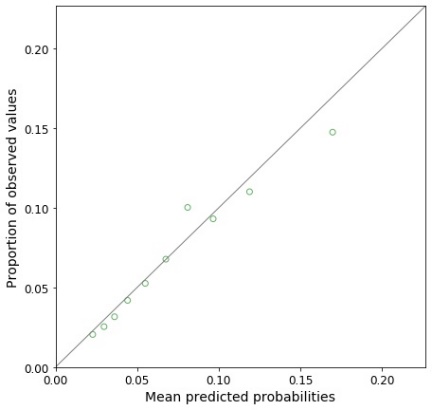 | F  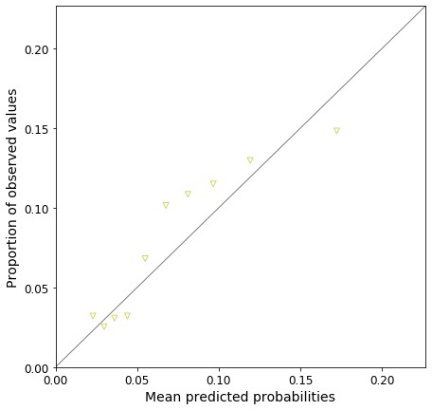 |
| G  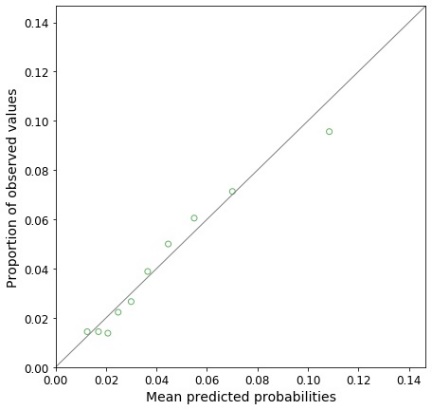 | H  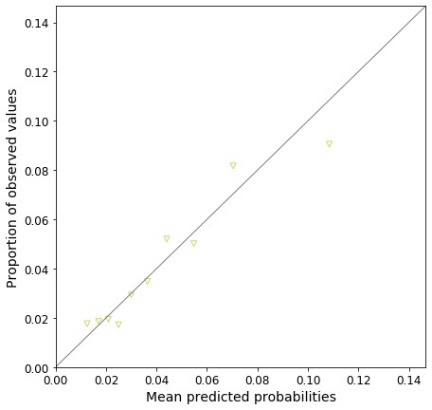 |
| **S1 Fig. Calibration plots of Cox models for infection-related hospital admission following a lower respiratory tract infection (LRTI), developed and validated with pre-pandemic data (from 1 January 2019 to 31 December 2019): (A) incident LRTI with no antibiotics using development dataset, (B) incident LRTI with no antibiotics using validation dataset, (C) incident LRTI with antibiotics using development dataset, (D) incident LRTI with antibiotics using validation dataset, (E) prevalent LRTI with no antibiotics using development dataset, (F) prevalent LRTI with no antibiotics using validation dataset, (G) prevalent LRTI with antibiotics using development dataset, (H) prevalent LRTI with antibiotics using validation dataset.** | |

| A  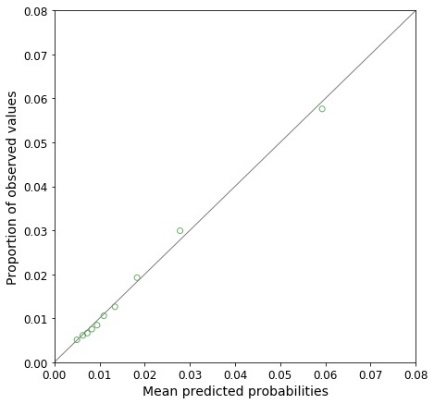 | B  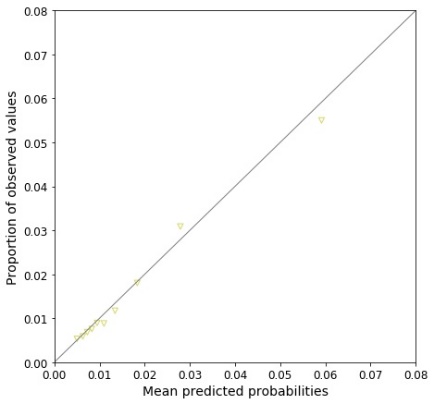 |
| --- | --- |
| C  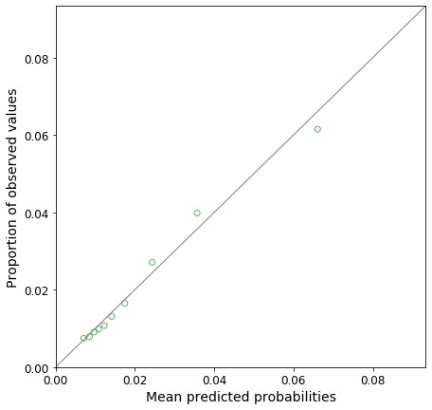 | D  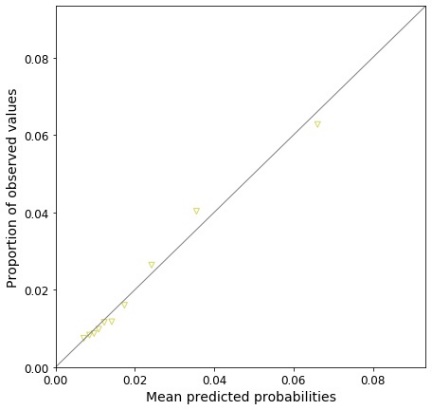 |
| E  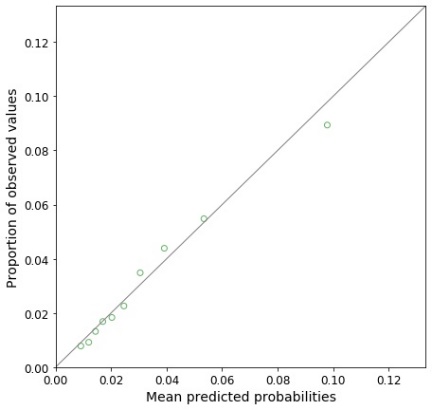 | F  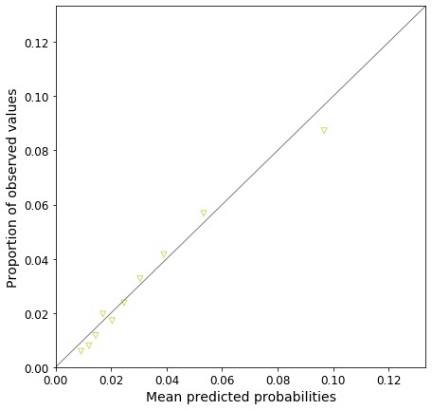 |
| G  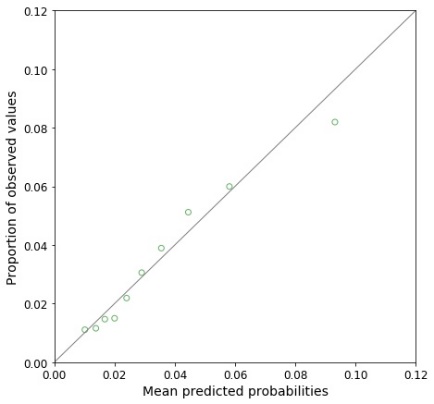 | H  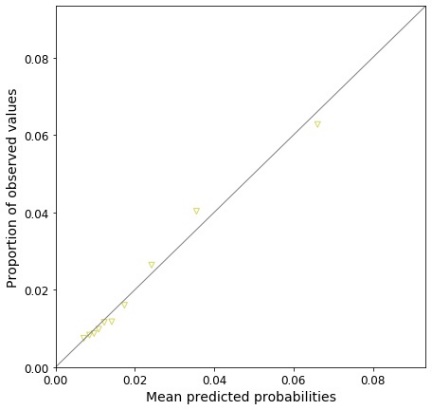 |
| **S2 Fig. Calibration plots of Cox models for infection-related hospital admission following upper respiratory tract infection (URTI; including URTI, cough, cold with cough, and sore throat), developed and validated with pre-pandemic data (from 1 January 2019 to 31 December 2019): (A) incident URTI with no antibiotics using development dataset, (B) incident URTI with no antibiotics using validation dataset, (C) incident URTI with antibiotics using development dataset, (D) incident URTI with antibiotics using validation dataset, (E) prevalent URTI with no antibiotics using development dataset, (F) prevalent URTI with no antibiotics using validation dataset, (G) prevalent URTI with antibiotics using development dataset, (H) prevalent URTI with antibiotics using validation dataset.** | |

| A  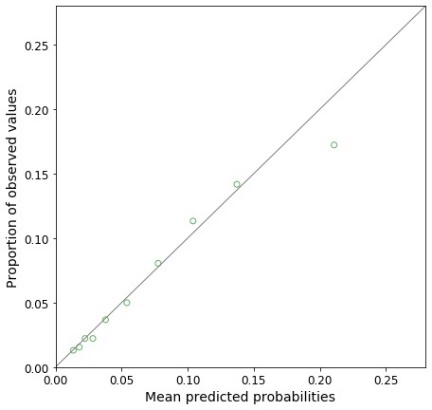 | B  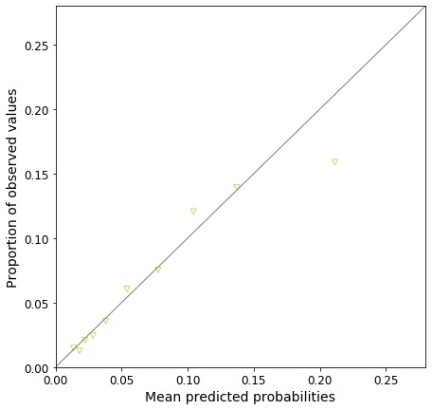 |
| --- | --- |
| C  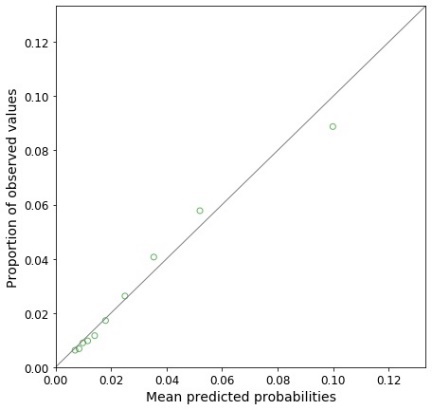 | D  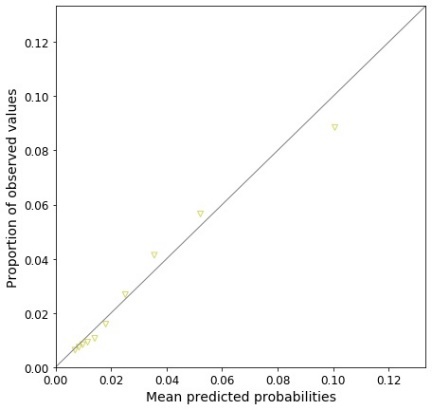 |
| E  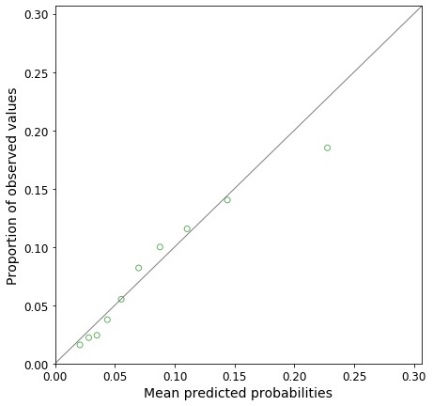 | F  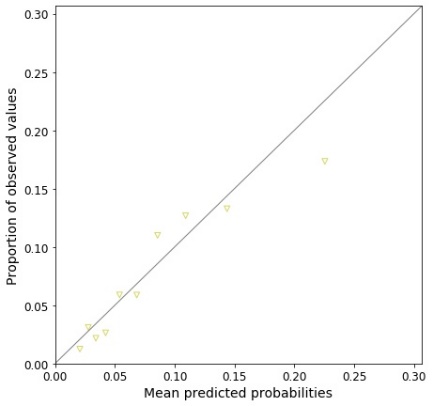 |
| G  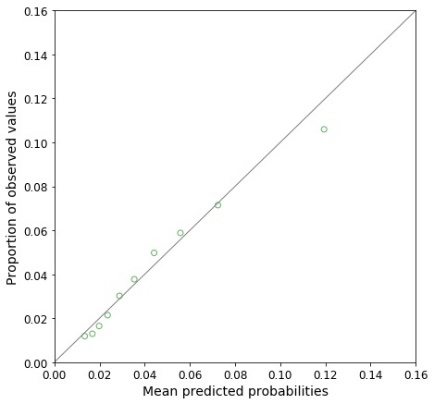 | H  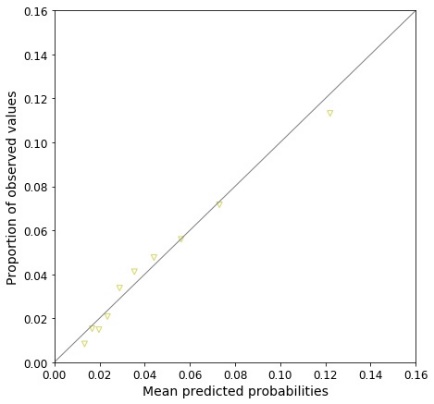 |
| **S3 Fig. Calibration plots of Cox models for infection-related hospital admission following a urinary tract infection (UTI) , developed and validated with pre-pandemic data (from 1 January 2019 to 31 December 2019): (A) incident UTI with no antibiotics using development dataset, (B) incident UTI with no antibiotics using validation dataset, (C) incident UTI with antibiotics using development dataset, (D) incident UTI with antibiotics using validation dataset, (E) prevalent UTI with no antibiotics using development dataset, (F) prevalent UTI with no antibiotics using validation dataset, (G) prevalent UTI with antibiotics using development dataset, (H) prevalent UTI with antibiotics using validation dataset.** | |

| A  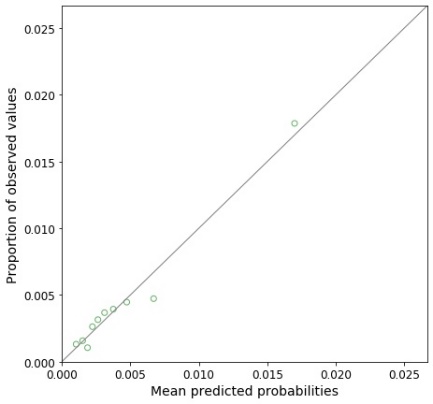 | B  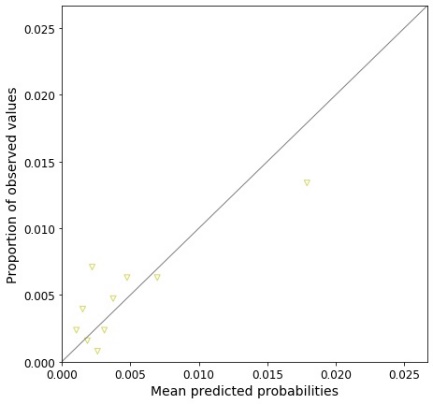 |
| --- | --- |
| **S4 Fig. Calibration plots of Cox models for infection-related hospital admission following a sinusitis, developed and validated with pre-pandemic data (from 1 January 2019 to 31 December 2019): (A) incident sinusitis with no antibiotics using development dataset, (B) incident sinusitis with no antibiotics using validation dataset.** | |

| A  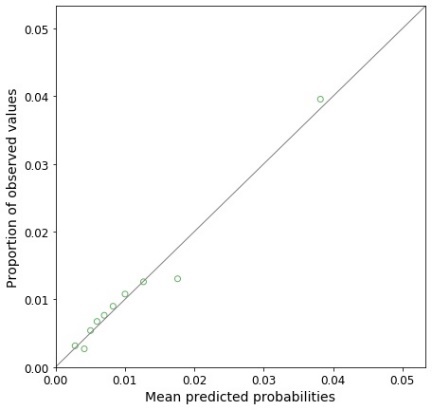 | B  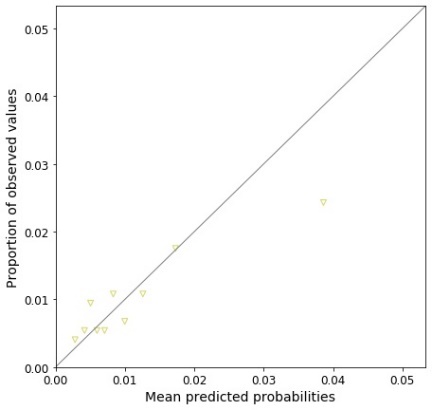 |
| --- | --- |
| C  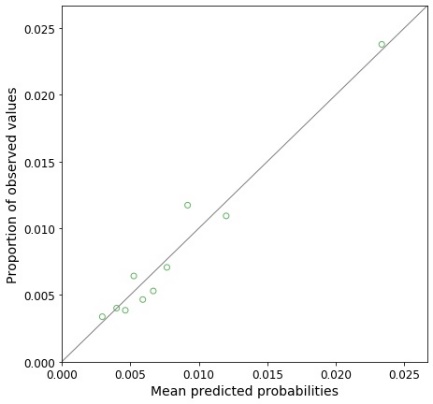 | D  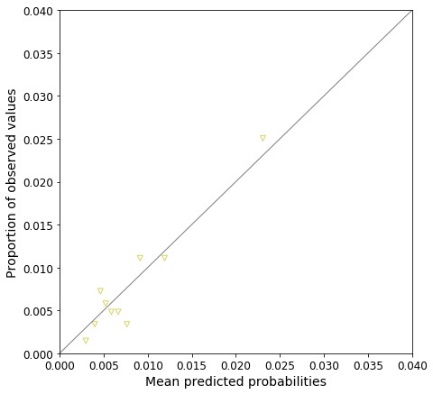 |
| **S5 Fig. Calibration plots of Cox models for infection-related hospital admission following an otitis media, developed and validated with pre-pandemic data (from 1 January 2019 to 31 December 2019): (A) incident otitis media with no antibiotics using development dataset, (B) incident otitis media with no antibiotics using validation dataset, (C) incident otitis media with antibiotics using development dataset, (D) incident otitis media with antibiotics using validation dataset.** | |

| A  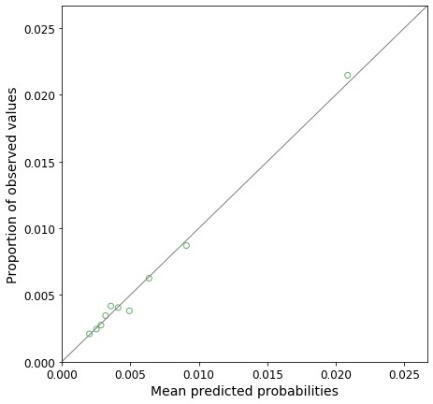 | B  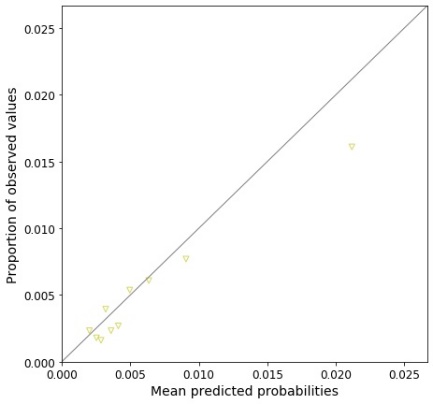 |
| --- | --- |
| C  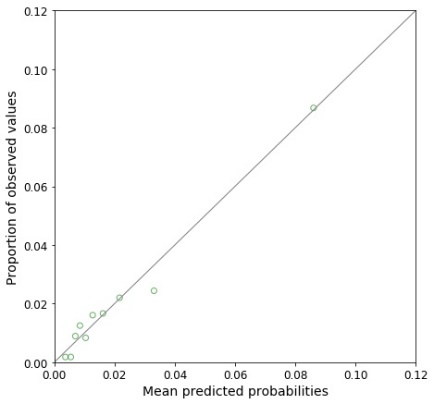 | D  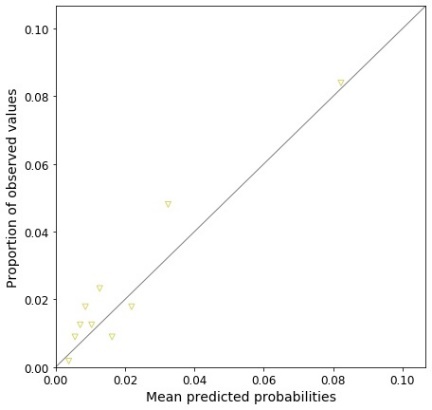 |
| **S6 Fig. Calibration plots of Cox models for infection-related hospital admission following an otitis externa, developed and validated with pre-pandemic data (from 1 January 2019 to 31 December 2019): (A) incident otitis externa with no antibiotics using development dataset, (B) incident otitis externa with no antibiotics using validation dataset, (C) prevalent otitis externa with no antibiotics using development dataset, (D) prevalent otitis externa with no antibiotics using validation dataset.** | |

| A  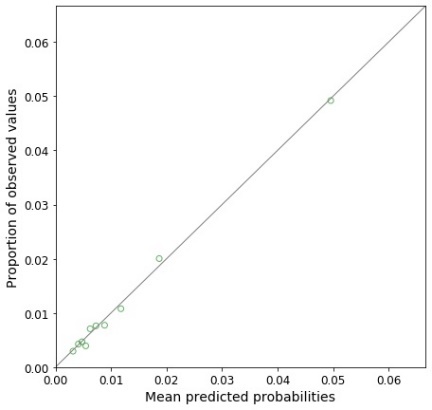 | B  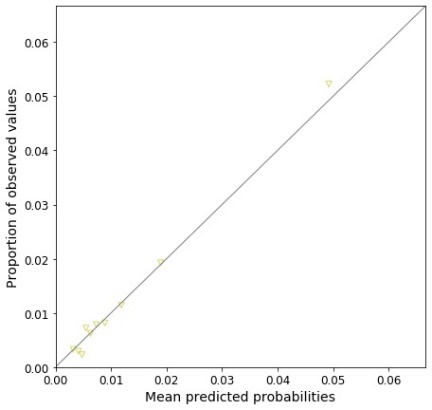 |
| --- | --- |
| C  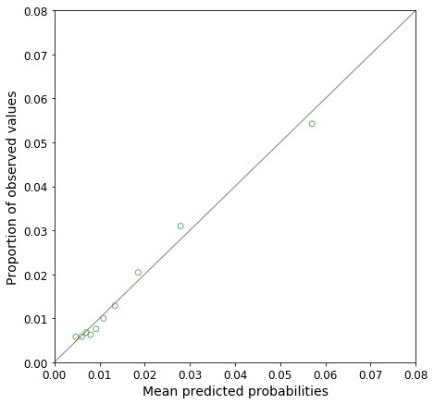 | D  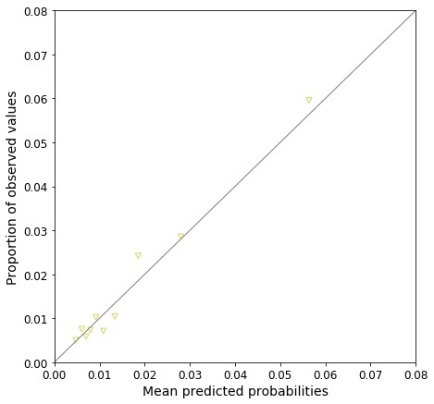 |
| **S7 Fig. Calibration plots of Cox models for infection-related hospital admission following an upper respiratory tract infection (URTI), developed and validated with pre-pandemic data (from 1 January 2019 to 31 December 2019): (A) incident URTI with no antibiotics using development dataset, (B) incident URTI with no antibiotics using validation dataset, (C) incident URTI with antibiotics using development dataset, (D) incident URTI with antibiotics using validation dataset.** | |

| A  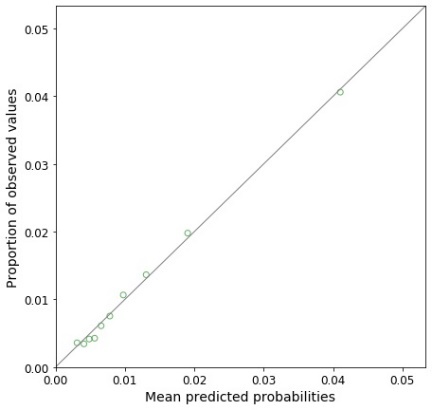 | B  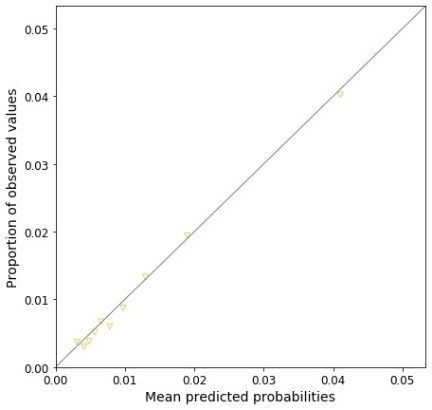 |
| --- | --- |
| C  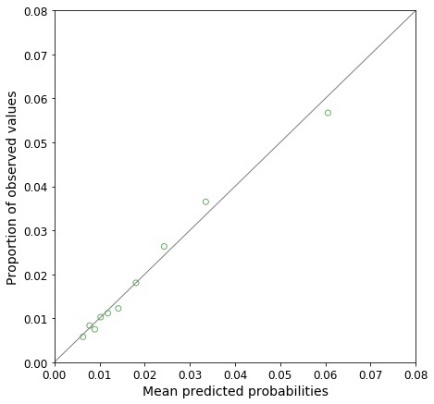 | D  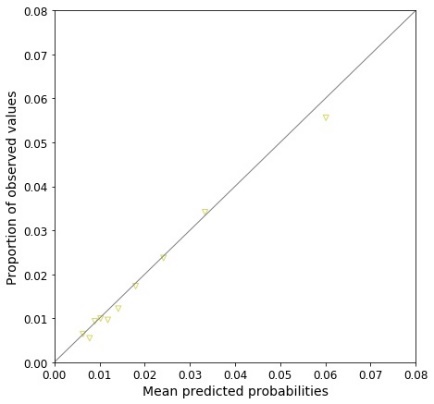 |
| E  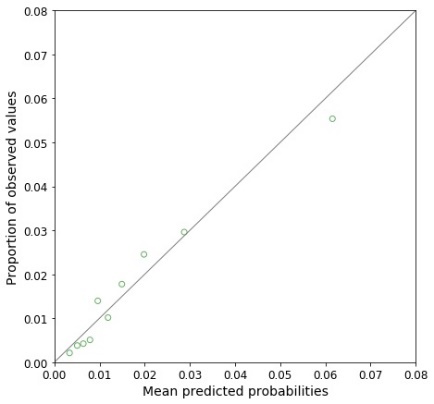 | F  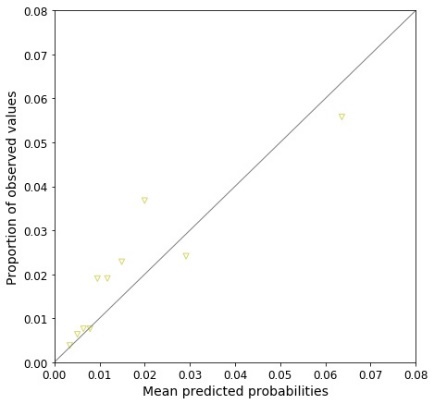 |
| G  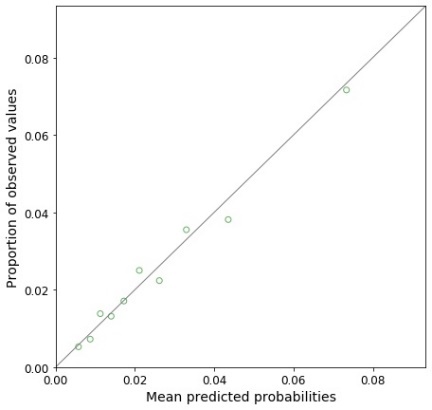 | H  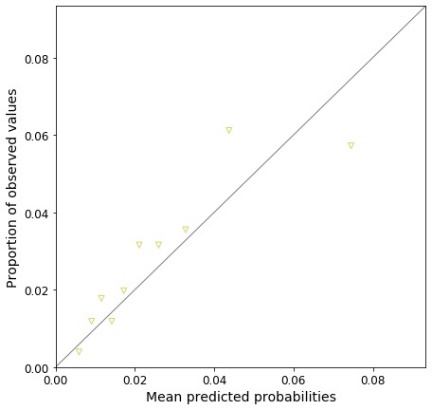 |
| **S8 Fig. Calibration plots of Cox models for infection-related hospital admission following a cough, developed and validated with pre-pandemic data (from 1 January 2019 to 31 December 2019): (A) incident cough with no antibiotics using development dataset, (B) incident cough with no antibiotics using validation dataset, (C) incident cough with antibiotics using development dataset, (D) incident cough with antibiotics using validation dataset, (E) prevalent cough with no antibiotics using development dataset, (F) prevalent cough with no antibiotics using validation dataset, (G) prevalent cough with antibiotics using development dataset, (H) prevalent cough with antibiotics using validation dataset.** | |

| A  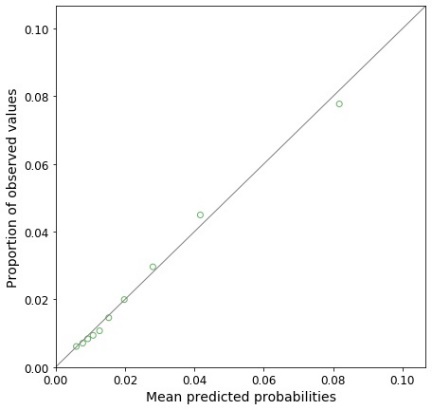 | B  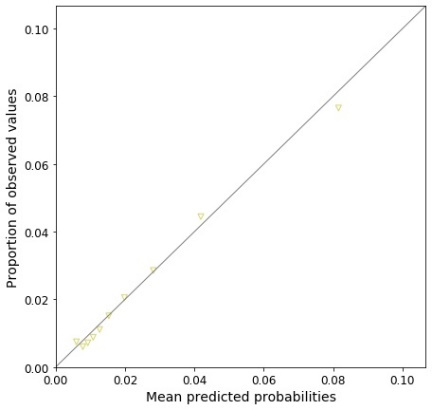 |
| --- | --- |
| C  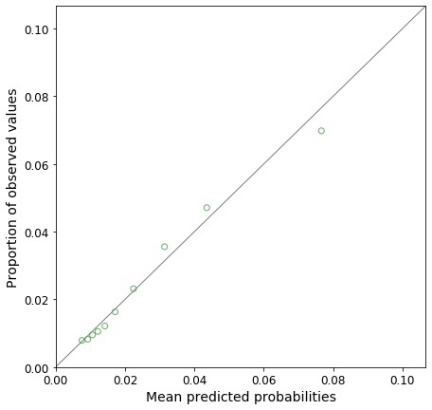 | D  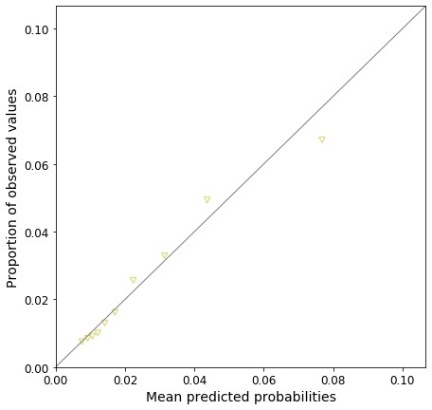 |
| E  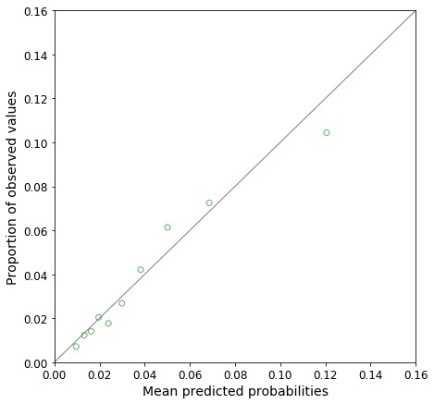 | F  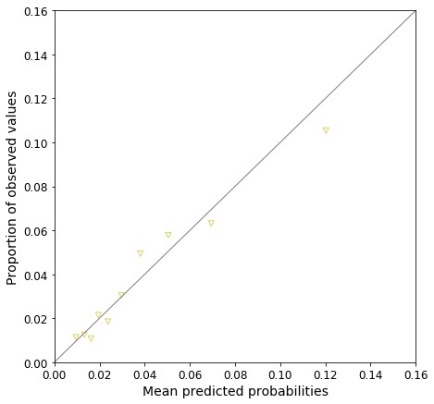 |
| G  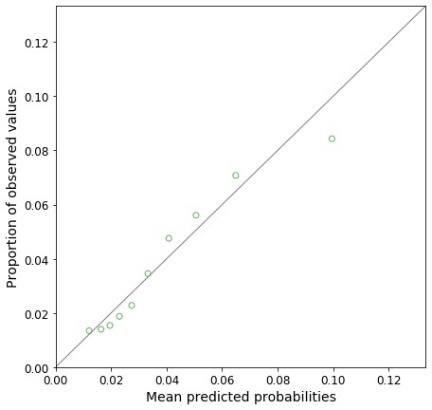 | H  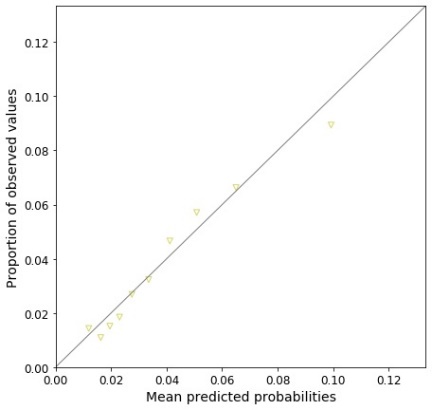 |
| **S9 Fig. Calibration plots of Cox models for infection-related hospital admission following a cold with cough, developed and validated with pre-pandemic data (from 1 January 2019 to 31 December 2019): (A) incident cold with cough without antibiotics using development dataset, (B) incident cold with cough without antibiotics using validation dataset, (C) incident cold with cough with antibiotics using development dataset, (D) incident cold with cough with antibiotics using validation dataset, (E) prevalent cold with cough without antibiotics using development dataset, (F) prevalent cold with cough without antibiotics using validation dataset, (G) prevalent cold with cough with antibiotics using development dataset, (H) prevalent cold with cough with antibiotics using validation dataset.** | |
| A  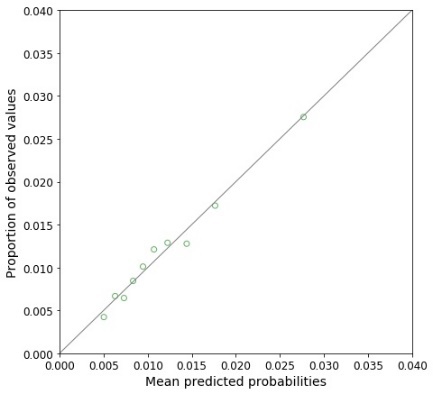 | B  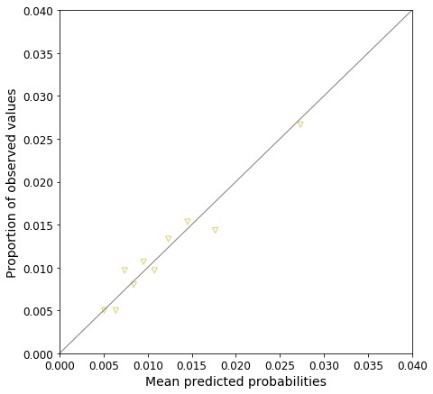 |
| C  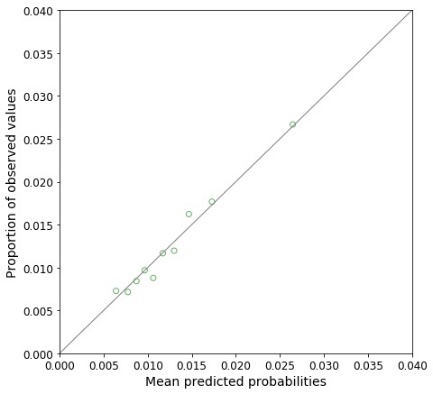 | D  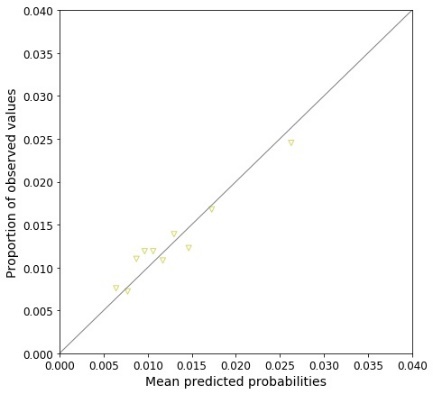 |
| E  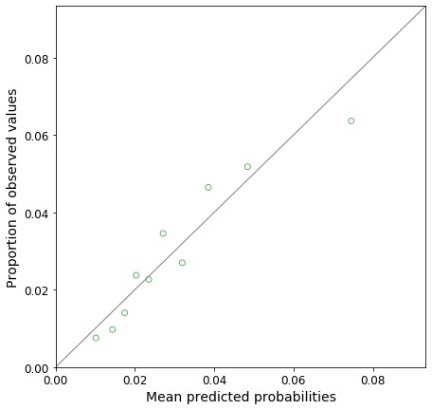 | F  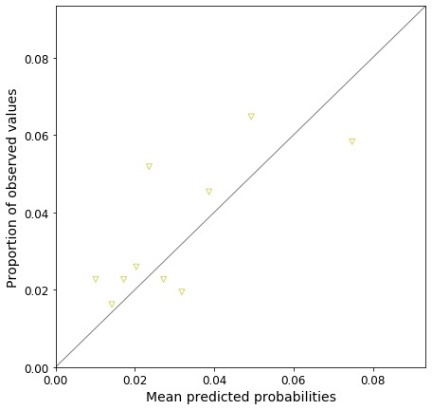 |
| G  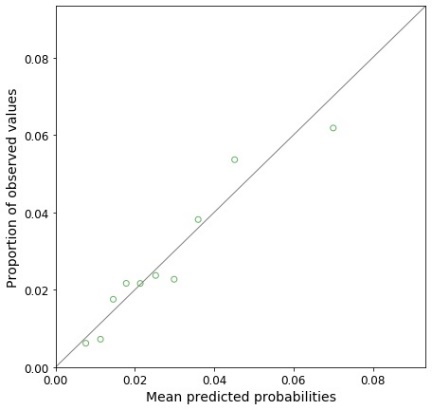 | H  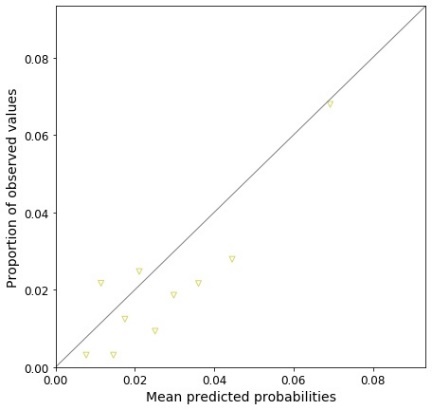 |
| **S10 Fig. Calibration plots of Cox models for infection-related hospital admission following a sore throat, developed and validated with pre-pandemic data (from 1 January 2019 to 31 December 2019): (A) incident sore throat with no antibiotics using development dataset, (B) incident sore throat with no antibiotics using validation dataset, (C) incident sore throat with antibiotics using development dataset, (D) incident sore throat with antibiotics using validation dataset, (E) prevalent sore throat with no antibiotics using development dataset, (F) prevalent sore throat with no antibiotics using validation dataset, (G) prevalent sore throat with antibiotics using development dataset, (H) prevalent sore throat with antibiotics using validation dataset.** | |
